# Single-nuclei UPR profiling by flow cytometry reveals bortezomib resistance mechanisms in multiple myeloma

**DOI:** 10.1038/s44321-026-00469-7

**Published:** 2026-06-29

**Authors:** Julien P Gigan, Paulina Garcia-Gonzalez, Angelo Pilotti, Lou Galliot, Alexandre Reynaud, Yoan Ghaffar, Felipe Flores-Santibáñez, Sara Ghafoori, Eve Seillier, Rosario Lavignolle-Heguy, Daniela Barros, Caroline Bret, Sharon Fichaux, Alexis Combes, Alessandro Bani, Rejane Rua, Evelina Gatti, Béatrice Nal-Rogier, Stéphane Rocchi, Jérôme Moreaux, Philippe Pierre, Rafael J Argüello

**Affiliations:** 1 Aix Marseille Université, CNRS, INSERM, CIML, Marseille, France; 2 Institute for Research in Biomedicine (iBiMED), Department of Medical Sciences, University of Aveiro, Aveiro, Portugal; 3 Department of Pathology, University of California San Francisco, San Francisco, CA USA; 4 Department of Medicine Division of Gastroenterology, University of California San Francisco, San Francisco, CA USA; 5 UCSF CoLab, University of California San Francisco, San Francisco, CA USA; 6 UCSF ImmunoX Initiative, University of California San Francisco, San Francisco, CA USA; 7 Université Côte d’Azur, INSERM U1065, C3M, Nice, France; 8 CNRS UMR 9002-CNRS-UM - Institute of Human Genetics, Montpellier, France; 9 Shanghai Institute of Immunology, Department of Microbiology and Immunology, Shanghai Jiao Tong University School of Medicine, Shanghai, PR China

**Keywords:** Cancer

## Abstract

The unfolded protein response (UPR) is a stress-adaptation pathway and therapeutic target in cancer, yet its pro-survival versus pro-death outcome is difficult to predict because the three ER sensors, PERK, IRE1α, and ATF6, are highly interconnected. Transcriptomic analyses identified sensor-specific gene signatures associated with patient survival across malignancies, and indicated that low IRE1α activity (low XBP1 signature or higher expression of RIDD targets) correlates with improved outcome. We developed SNUPR (single nuclei analysis of the unfolded protein response), an accessible flow cytometry approach that profiles all three branches in nuclear suspensions. SNUPR reveals marked heterogeneity of UPR activation across cancer cell lines that cannot be inferred from sensor expression. This heterogeneity is derived from differences in the strength and duration of PERK-mediated translational inhibition, which gates downstream translation-dependent IRE1α and ATF6 transcriptional programs. Finally, in multiple myeloma, we show that bortezomib-tolerant cells depend on IRE1α activity for survival, linking UPR state to proteasome-inhibitor resistance and positioning SNUPR to guide branch-selective targeting.

## Synopsis

SNUPR enables flow-cytometric profiling of UPR branch activation in isolated nuclei and reveals how translational control shapes ER-stress responses and bortezomib resistance in multiple myeloma.

- SNUPR allows simultaneous profiling of PERK, IRE-1 and ATF6 activation at single-cell resolution.
- Marked cell type- and stressor-dependent UPR heterogeneity was detected across cancer cell lines, which was not predicted by ER-stress sensor abundance.
- Inhibition of protein synthesis via PERK control the activation levels of the IRE-1/XBP1s and ATF6 pathway.
- IRE-1 activation and associated transcriptional signatures predict outcomes in patients with multiple myeloma treated with Bortezomib.
- IRE-1 activity, but not PERK or ATF6, is essential for the acquisition of bortezomib resistance in multiple myeloma cell lines.

## Abstract (web summary)

SNUPR enables flow-cytometric profiling of UPR branch activation in isolated nuclei and reveals how translational control shapes ER-stress responses and bortezomib resistance in multiple myeloma.

## Graphical abstract

**Figure:**
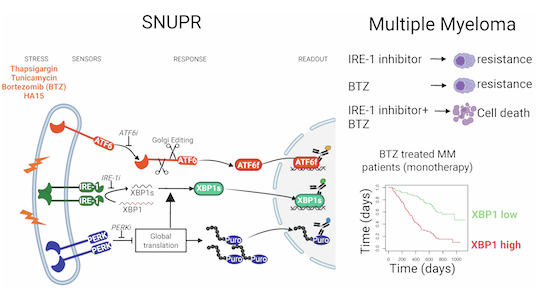

**Figure:**
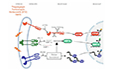

The paper explained **Problem**

The unfolded protein response (UPR) helps cancer cells adapt to stress and survive chemotherapy. However, because the three UPR branches (PERK, IRE1α, and ATF6) are highly interconnected, it remains difficult to predict how UPR activation influences treatment response and drug resistance.

**Results**

We developed SNUPR, a method that simultaneously measures activation of the three UPR branches at single-cell resolution. SNUPR revealed strong heterogeneity of UPR activation between cancer cell types and showed that PERK-mediated translation inhibition controls downstream IRE1α and ATF6 responses. In multiple myeloma, resistant cells depended on IRE1α activity to survive bortezomib treatment, and high IRE1α/XBP1 signatures were associated with poor clinical outcome.

**Impact**

Our findings identify IRE1α signaling as a potential therapeutic vulnerability in bortezomib-resistant multiple myeloma. SNUPR may help stratify patients and guide the development of branch-selective UPR-targeted therapies in cancer.

## Introduction

The tumor microenvironment contains biochemical stressors that can disrupt protein folding within the endoplasmic reticulum (ER) and affect cell viability. Accumulation of misfolded and unfolded proteins in the ER triggers a cellular response known as the unfolded protein response (UPR) (Chen and Cubillos-Ruiz, 2021).

The UPR comprises distinct signaling branches, each activated by the dissociation in the ER lumen of the HSPA5 chaperone (BiP) from three ER-resident sensors. In addition to the classical model of UPR activation via BiP dissociation, it is now recognized that misfolded proteins can directly interact with the luminal domains of ER stress sensors, such as IRE1α and PERK, leading to their oligomerization and activation independently of BiP release (Gardner and Walter, 2011; Karagöz et al, 2017; Wang et al, 2018). These sensors include activating transcription factor 6 (ATF6), inositol-requiring enzyme 1-alpha (IRE1α, ERN1), and (PKR)-like endoplasmic reticulum kinase (PERK, EIF2AK3). Each sensor triggers a unique cellular response and leads to the activation of specific transcription factors, ultimately modulating a range of genes involved in ER homeostasis (Hetz and Papa, 2018). (i) IRE1α splices the mRNA encoding for the transcription factor X-box binding protein-1 (XBP1) involved in the expression of genes regulating ER size and function. (ii) ATF6 exits the ER to reach the Golgi apparatus and undergoes proteolytic release in the cytosol as ATF6f, which in turn acts as a transcription factor that induces the synthesis of *XBP1* and ER chaperone genes like *HSPA5* (BiP) and others. (iii) PERK activation mediates the phosphorylation of eukaryotic initiation factor 2-alpha (eIF2α). While eIF2α phosphorylation leads to a decrease in global protein synthesis, it also favors the translation of specific transcripts like activating transcription factor 4 (ATF4), C/EBP homologous protein (CHOP, DDIT3), or the phosphatase co-factor PPP1R15a (GADD34) mRNAs (Han et al, 2013a; Costa-Mattioli and Walter, 2020).

As a key regulator of proteostasis and cell death, the UPR has gained a lot of interest as a promising target in anti-tumoral therapy. Depending on the duration and degree of the ER stress, the UPR can provide either survival signals or trigger cell death through apoptosis. Tumor progression requires a high level of protein synthesis and exposes cells to multiple extrinsic and intrinsic stressors that can lead to chronic UPR activation and malignant progression (Chen and Cubillos-Ruiz, 2021; Cherubini and Zito, 2022). On the other hand, non-cancerous cells can also show activation of UPR pathways, but in general rely less on constant high levels of translation. This difference offers an advantage for potential targeted therapies, like proteasome inhibitors, by modulating the UPR to target cancer cells (Bianchi et al, 2009). Sustained pharmacological induction or repression of the UPR could exert beneficial anti-tumoral effects. Henceforth, the interest to combine standard therapies with drugs directed towards unresolved ER stress or UPR modulation to restrain tumor growth; some of which have already been shown to be effective in pre-clinical tumor models (Mbofung et al, 2017; Lin et al, 2021; Hurst et al, 2019; Shi et al, 2019).

Our understanding of the interplay between UPR effectors, how it influences the balance between cell survival and cell death; and our capacity to predict ER-stress responses in physiological contexts remains limited due to technical limitations. We developed “Single Nuclei Analysis of the Unfolded Protein Response” (SNUPR), a method that allows the simultaneous measurement of ATF6f, IRE-1/XBP1s and PERK pathways activation, as well as their interplay on a single-cell and time-resolved basis.

Using SNUPR, we were able to highlight the heterogeneity of UPR activation in response to standard pharmacological ER stressors on different cancer cell lines. This allowed us to shed light on how the intensity and duration of translation inhibition via the PERK pathway can shape responses from the other UPR branches during ER-stress across various cancer cell lines. Additionally, our analysis allowed us to revisit the importance of the IRE1/XBP-1s axis in the resistance to bortezomib chemotherapy in multiple myeloma (Besse et al, 2021; Borjan et al, 2019). Furthermore, transcriptomic analysis of multiple myeloma cohorts confirmed our hypothesis that the XBP1 gene signature alone can predict the outcome of multiple myeloma patients treated with bortezomib as monotherapy. By revealing the complex interplay and hierarchy between UPR branches via SNUPR, our findings underline how strategic manipulation of the UPR could present a promising therapeutic strategy for treating cancer or to stratify multiple myeloma patients to predict treatment efficacy.

## Results

### Correlation between ER-stress sensors activation and survival prognosis in acute myeloid leukemia and breast cancer patients

**Figure 1:**
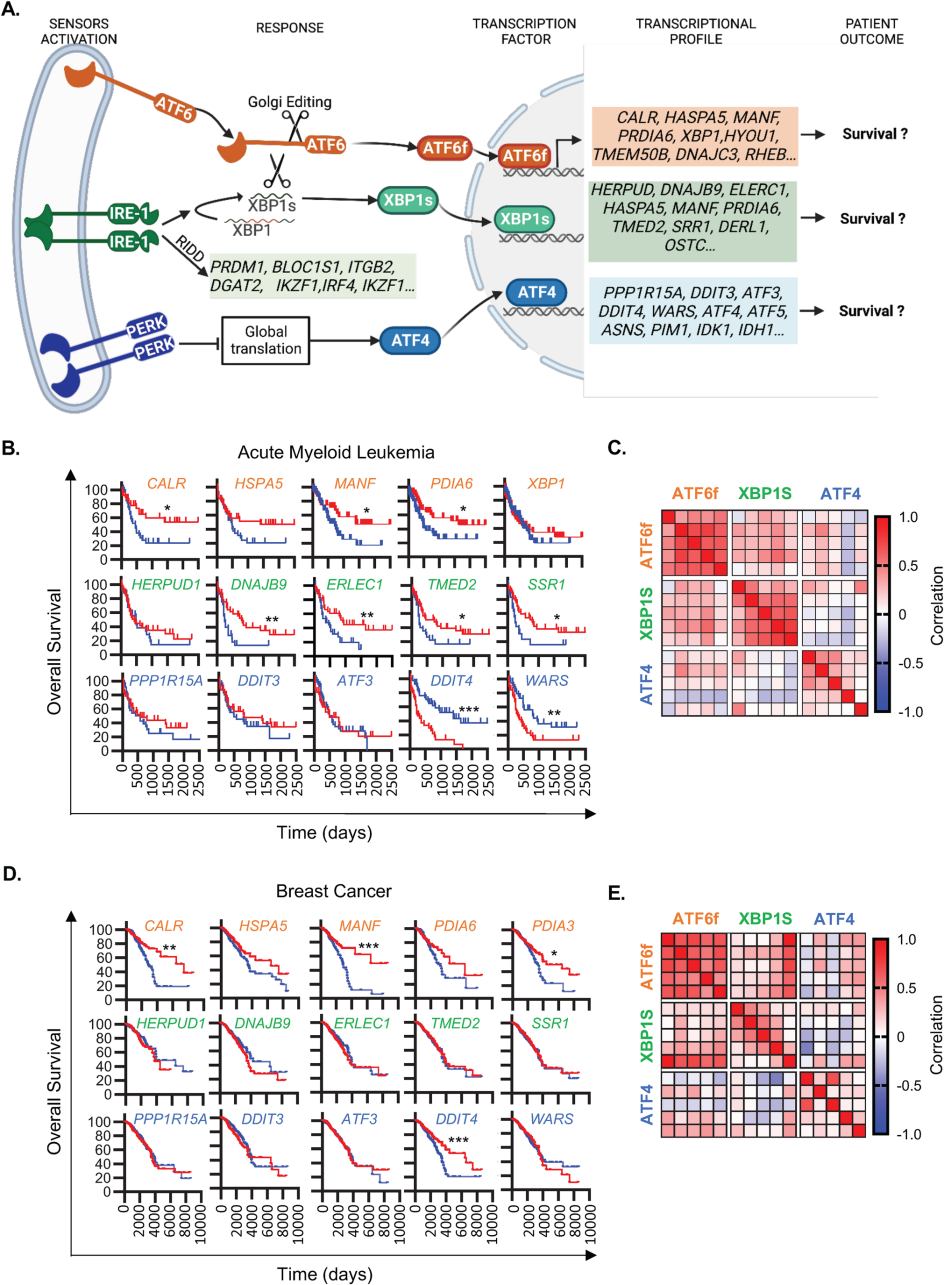
UPR signatures correlate with survival prognosis of myeloid leukemia and breast cancer patients. (**A**) Scheme of the UPR three main branches. (**B**–**E**) Kaplan–Meier survival curves and correlation matrices of genes under the control of ATF6, XBP1s, or PERK corresponding to AML and breast cancer cohorts were generated using the Xena browser (USCC). Kaplan–Meier curves were done based on dichotomized gene expression, specifically for values below quartile 1 (blue) and above quartile 3 (red) of both malignancies. Genes indicated are described to be under the control of ATF6 (orange), IRE-1/XBP1s (green), and PERK/ATF4 (blue). (**B**) Kaplan–Meier curve and (**C**) correlation matrix for the AML cohort. (**D**) Kaplan–Meier curve and (**E**) correlation matrix for the breast cancer cohort. Statistical analysis was performed using the Log-rank test. *P < 0.05, **P < 0.01, and ***P < 0.001.

Tumor growth often induces oxidative stress and glucose deprivation which in turn can cause oxidative damage and glycosylation defects that result in protein misfolding, ER stress and UPR activation (Alam et al, 2022) (Fig. 1A). The particular role of the different UPR branches in cancer is controversial, with both positive and negative outcomes described in the literature (Martinez-Turtos et al, 2022; Lin et al, 2021; Shi et al, 2019; Boyle et al, 2020; Salvagno et al, 2022). To gain insights into whether all or specific UPR branches are associated with prognosis in human cancer, we analyzed acute myeloid leukemia (AML) and breast cancer cohorts, representing a hematological and a solid tumor, respectively, which have distinct microenvironment-associated stresses, but display substantial variability in clinical outcomes and have been previously reported to engage UPR signaling (Philippe et al, 2024; Chen et al, 2014). We evaluated patient survival over time, generating Kaplan–Meier curves on those subgroups of patients defined on mRNA expression levels of UPR branches-associated genes (Fig. 1B,D; Appendix Fig. S1). Only a high expression of certain ATF6-associated genes was correlated with increased patient survival for both types of cancer, with 5 out of 14 target mRNAs for AML (Fig. 1B and Appendix Fig. S1A) and 3 out of 5 target mRNAs for breast cancer (Fig. 1D) presenting a significant difference. We also found that several target genes of XBP1s correlated with longer median survival in AML patients, 7 of which showed statistical significance (Fig. 1D; Appendix Fig. S1B). Overall, patients with cells with higher expression of genes that are transcriptionally dependent on XBP1s and ATF6f showed cross-correlation of expression (Fig. 1C,E), but not ATF4, and had a markedly enhanced survival rate.

Besides unconventional splicing of *XBP1* mRNA, another hallmark of IRE1α activation is the cleavage of different RNAs through a process termed RIDD (Regulated IRE1-Dependent Decay), leading to degradation of mRNAs coding for genes such as *ITGB2* and *IKZF1* (Appendix Fig. S2) (Eletto et al, 2016; Gómora-García et al, 2021; Hollien et al, 2009; Moore and Hollien, 2015; Quwaider et al, 2022; Maurel et al, 2014). In contrast to what we observed with XBP1 targets, we noted a variable and non-significant trend in survival rates when correlating survival with the expression level of RIDD targets (Appendix Fig. S2) in AML. These results imply that the canonical IRE1α activity, through *XBP1* splicing, is most likely contributing to patient survival in AML (Eletto et al, 2016; Gómora-García et al, 2021; Hollien et al, 2009; Moore and Hollien, 2015; Quwaider et al, 2022; Maurel et al, 2014).

In contrast, most of the PERK/ATF4 target genes, such as *PPP1R15A* (GADD34) and *DDIT3* (CHOP), showed no correlation or anti-correlation with survival prognosis (Fig. 1B,D; Appendix Fig. S1). Nonetheless, certain non-exclusive ATF4 targets like *DDIT4, WARS*, *PIM*, and *PSAT1* (Dalet et al, 2017), were associated with lower survival rates in AML patients with high expression (Fig. 1B; Appendix Fig. S1C).

Altogether, the association of IRE-1α/XBP1s and ATF6 signatures, but not ATF4 with survival suggests that in the presence of ER stress, UPR sensors may not be all activated simultaneously. Moreover, our result suggests that a cancer-specific association between IRE1α and ATF6 activation and survival rates. Hence, a potential dichotomy and heterogeneity in ER stress sensors activation may influence disease progression and therapeutic response.

### Profiling of UPR signaling branches via SNUPR

**Figure 2:**
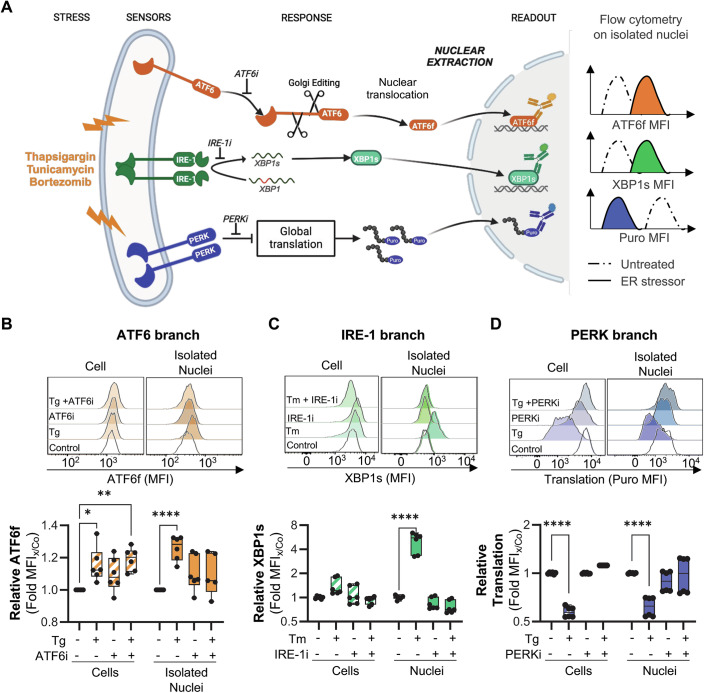
SNUPR, a method to profile sensor activation during ER stress. (**A**) Scheme of the SNUPR method. Following nuclei extraction, the activation of UPR branches is profiled by flow cytometry measurements of XBP1s and ATF6f translocation as well as puromycin levels as a readout for protein synthesis. (**B**) THP1 were treated with 400 nM of thapsigargin for 4 h in the presence (or absence) of the ATF6 inhibitor Ceapin A7 (6 µM). Afterward, nuclei were extracted, and ATF6f levels were analyzed on both permeabilized nuclei and cells. (**C**) THP1 were treated with 100 ng/mL of tunicamycin for 6 h in the presence (or absence) of the IRE1α inhibitor 4µ8c (10 µM). Afterward, nuclei were extracted, and XBP1s levels were analyzed on both permeabilized nuclei and cells. (**D**) HeLa cells were treated with 400 nM of thapsigargin for 30 min in the presence (or absence) of the PERK inhibitor GSK2656157 (100 µM) and treated with puromycin for 15 min. Afterward, nuclei were extracted, and puromycin levels were analyzed on both permeabilized nuclei and cells. For the (**B**–**D**), each data point represents a replicate of three independent experiments (*n* = 3), and in box plots the center line indicates the median, box limits indicate the 25th and 75th percentiles, and whiskers indicate the minimum and maximum values. Statistical analysis was performed using Mann–Whitney test. *P < 0.05, **P < 0.01, ***P < 0.001 and ****P ≤ 0.0001. Source data are available online for this figure.

The heterogeneity and hierarchical activation of IRE1α, ATF6, and PERK in response to ER stress is not well understood (Adwal et al, 2020; Obeng et al, 2006; Huang et al, 2020). Additionally, the use of transcriptional approaches to investigate this phenomenon can be misleading due to the central role of post-transcriptional and protein synthesis regulation in ER stress responses. We developed SNUPR (Single Nuclei UPR Profiling), a flow cytometry-based method that allows to delineate the activation of all three UPR branches, ATF6, IRE-1, and PERK with single-cell resolution. SNUPR uses multi-parametric flow cytometry of single nuclear suspensions to measure XBP1s and ATF6f nuclear translocation as well as intracellular puromycin incorporation (Schmidt et al, 2009; Dalet et al, 2017; Argüello et al, 2018) to simultaneously assess transcription factor translocation with overall inhibition of mRNA translation as an indirect measure of PERK activation (Fig. 2).

First, we isolated nuclei from plasmacytoid dendritic cell line (CAL-1) and monocytic AML cell line (THP-1) cells and used microscopy to confirm the enrichment of nuclei post-extraction, further validating their purity through immunoblot and flow cytometry assays targeting nuclear, plasma membrane, and other organelles (Appendix Fig. S3A–D). There was minimal presence of plasma membrane, mitochondrial, lysosomal, and endoplasmic reticulum contaminants in nuclear suspensions, indicating that our protocol allowed efficient nuclei isolation with minimal contamination from other intracellular compartments, permitting accurate quantification of transcription factors' translocation.

Next, we employed fluorescent labeled antibodies to monitor the translocation of XBP1s and ATF6f transcription factors by flow cytometry (Fig. 2A). We extracted nuclei from ER stress-induced THP-1 cells, treated with or without thapsigargin (Tg) or tunicamycin (Tm), in the presence or absence of specific inhibitors of IRE1α (4µ8c) (Cross et al, 2012), SP1/2 that mediate ATF6 cleavage (Ceapin A7) (Gallagher et al, 2016) or PERK (GSK2656157) (Axten et al, 2013). By comparing the staining of the transcription factors between cells and nuclei, we obtained an enhanced signal-to-noise ratio for both XBP1s and ATF6f post-nuclear extraction (Fig. 2B-C). The inhibition of UPR signaling pathways resulted in a decrease in nuclear staining of the corresponding transcription. These results demonstrate, on one hand the specificity of the measurements, and on the other, the higher signal-to-noise ratios obtained when analyzing nuclear extractions; further corroborating the effectiveness of SNUPR in monitoring IRE1α activation and ATF6 cleavage.

Our efforts to find suitable antibodies for detecting ATF4, as a direct readout of PERK activation by flow cytometry were however unsuccessful, and we turned toward protein synthesis inhibition measurement instead (Han et al, 2013b) (Fig. 2D). Puromycin is a tRNA-aminoacyl analog and measuring intracellular puromycin incorporation into peptides, we monitored global translation inhibition mediated by PERK-dependent eIF2α phosphorylation (Dalet et al, 2017; Schmidt et al, 2009). We observed a very strong and significant correlation between nuclear and cellular levels of puromycinilated peptides (David et al, 2012) (Appendix Fig. S3E). A marked decrease in puromycin signal was observed in nuclei from cells treated with the ER stressors Tg and Harringtonine (Fig. 2D; Appendix Fig. S3E). Upon PERK inhibition (GSK2656157, PERKi), the puromycin signal remained unaltered after Tg treatment, supporting the validity and specificity of our approach as readout for PERK activation (Fig. 2D). Consequently, SNUPR, by monitoring XBP1s and ATF6f translocation together with puromycin incorporation on isolated nuclei, allows the simultaneous measurement of all three UPR signaling branches activation with high accuracy.

### Deciphering cell type- and stressor-dependent UPR heterogeneity using SNUPR profiling

**Figure 3:**
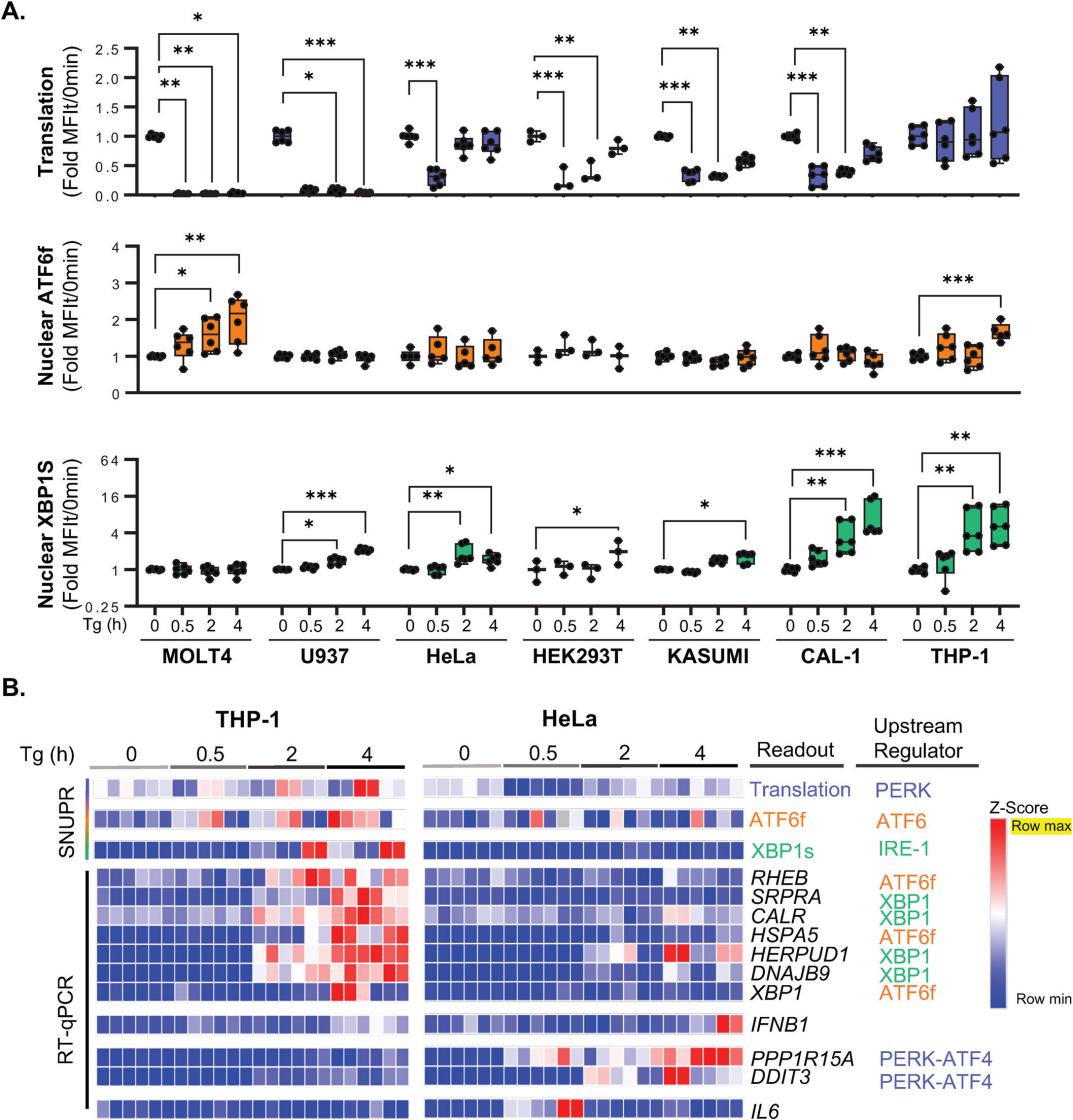
Induction of acute ER stress induces different UPR profiles in cell lines. (**A**) 7 different types of cancer cell lines were treated with thapsigargin (Tg) for 30 min, 2 h, or 4 h to induce acute ER stress prior to nuclei extraction and SNUPR profiling of UPR activation. Shown are box plots of MFI fold changes (MFIt/t0 min) values representing translation level, ATF6f, and XBP1s translocation. Each data point represents a replicate of three independent experiments (*n *= 3). In box plots, the center line indicates the median, box limits indicate the 25th and 75th percentiles, and whiskers indicate the minimum and maximum values. (**B**) Heatmap representation of SNUPR measurement as well as the expression level of ATF4-, ATF6-, and XBP1s-target mRNAs measured by RT-qPCR. Each point corresponds to one replicate of three independent experiments (*n* = 3). Statistical analysis was performed using the Kruskal–Wallis test for each cell line. *P < 0.05, **P < 0.01, and ***P < 0.001. Source data are available online for this figure.

Given the clinical relevance of a potential association between UPR heterogeneity and patient response to treatment and survival, we treated seven human cell lines of diverse origin with thapsigargin (Tg) and tunicamycin (Tm) and applied SNUPR and qPCR analysis to monitor UPR activation over time (0–4 h) (Fig. 3; Appendix Fig. S4). SNUPR revealed divergent UPR activation patterns in response to the two stressors, but also great variations among the cell lines tested with the same stressor (Fig. 3). Solely based on the duration and intensity of the translation inhibition driven by the two compounds, we identified three distinct response patterns (Fig. 3A). Group 1 (MOLT-4 and U937 cells) showed complete translation arrest within 30 min to 4 h of Tg treatment. Group 2 (HeLa, HEK293T, KASUMI, and CAL-1) underwent up to 70% translation inhibition after 30 min, recovering to initial levels within the subsequent 3 h. Lastly, Group 3 (THP-1 monocytic AML line) presented little to no reduction of translation during Tg treatment, mirroring the previously reported behavior of murine dendritic cells (Mendes et al, 2021).

Upon examining IRE1α and ATF6 activation patterns, a significant increase in XBP1s translocation was noted within 4 h of Tg treatment across most cell types, but a significant increase in ATF6f was only detected in two of the seven cell lines (MOLT-4 and THP-1) (Fig. 3A).

**Figure EV1:**
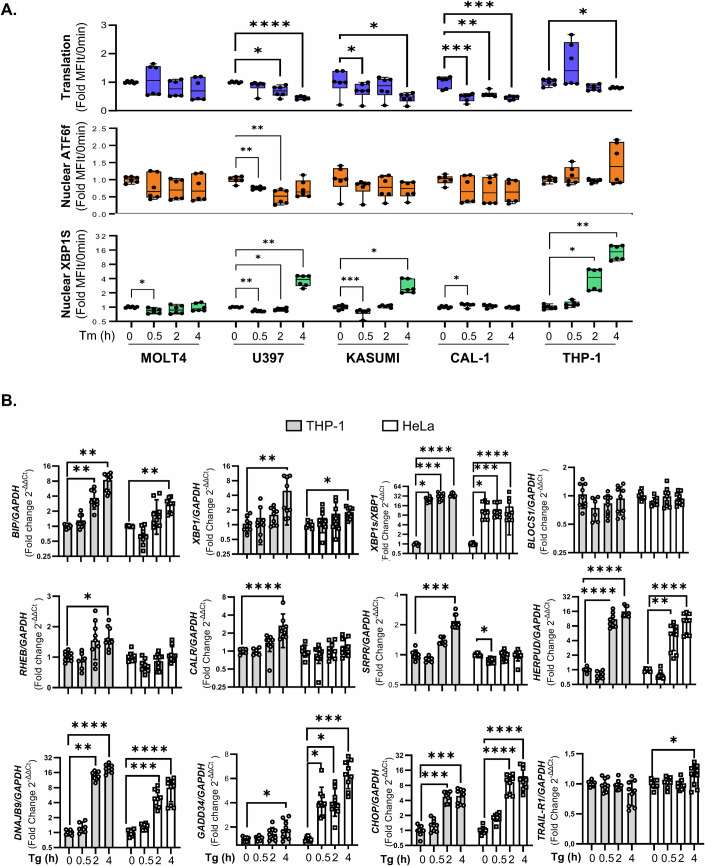
Induction of acute ER stress with Tunicamycin induces different UPR profiles in cell lines. (**A**) Distinct cancer cell lines were treated with 100 ng/ml of tunicamycin (Tm) for 30 min, 2 h, and 4 h prior to nuclei extraction and further SNUPR profiling of UPR activation by measuring translation (puromycin incorporation) and translocation of ATF6f and XBP1s by flow cytometry. In box plots, the center line indicates the median, box limits indicate the 25th and 75th percentiles, and whiskers indicate the minimum and maximum values. Statistical analysis was performed using the Kruskal–Wallis test for each cell line. *P < 0.05, **P < 0.01, ***P < 0.001 and ****P < 0.0001. (**B**) qPCR quantification of relative mRNA levels of UPR-associated transcripts on HeLa and THP-1 cells treated for 30 min, 2 h, and 4 h with thapsigargin (Tg, 400 nM). Statistical analysis was performed using the Kruskal–Wallis test for each cell line. *P < 0.05, **P < 0.01, ***P < 0.001 and ****P < 0.0001. Source data are available online for this figure.

To determine if the different patterns of UPR were solely dependent on the cell line, we decided to use another ER-stress inducer. When Tunicamycin (Tm) was used as the stressor, we observed a different pattern of response (Fig. EV1A). While most cells increased XBP1s expression after 4 h, no significant changes were found in ATF6f, except for the U937 cell line. As for translation inhibition, all cell lines analyzed displayed a moderate translation decrease within 4 h of Tm treatment. Notably, THP-1 cells displayed increased translation within the first 30 min of treatment, before returning to basal levels (Fig. EV1A). To validate SNUPR observations, we assessed the induction of mRNA levels of target genes of XBP1s, ATF6f, and ATF4 in HeLa and THP-1 cells, since these cells displayed contrasting UPR induction patterns (Figs. 3B and  EV1B). The RT-qPCR results mirrored SNUPR results, with THP-1 cells experiencing a significant increase in ATF6f and XBP1s specific transcripts, such as *XBP1, HSPA5, RHEB*, or *CALR*, under Tg-induced stress; while HeLa cells primarily induced ATF4-dependent transcription of *DDIT3* (*CHOP*) and *PPP1R15A* (*GADD34*) (Figs. 3B and  EV1B).

We next wondered whether the level of expression of individual ER stress sensors could reflect the pattern of activation of the different cell lines. However, levels of IRE1α or PERK measured by immunoblot (Appendix Fig. S4A) did not show any significant correlation with the capacity of the cells to block translation or translocate XBP1s upon ER-stress (Appendix Fig. S4B). This highlights the challenge of predicting functional cellular responses based solely on steady-state phenotypic markers, and supports the advantage of using functional readouts such as the ones measured in SNUPR to follow UPR activation dynamically and dissect the interplay among the three different individual UPR branches.

Interestingly, we observed that the absence and presence of *PPP1R15A* and *DDIT3*, in THP-1 cells and HeLa cells, respectively, coincided with the degree of measurable translation arrest in response to the stressors (Fig. 3B), where THP-1 cells did not block protein synthesis, HeLa cells did. Additionally, cells whose protein synthesis remains most active during stress induce more nuclear translocation of XBP1s, while the opposite trend is observed with ATF6f (Appendix Fig. S4C). Moreover, despite the rapid induction of *XBP1* mRNA splicing 2 h post-Tg treatment in both HeLa and THP-1 cells (Fig. EV1B), significant increases in nuclear XBP1s levels were only observed in THP-1 cells that did not block protein synthesis. Altogether, these results suggest that the magnitude of IRE-1/XBP1s response is inversely proportional to the degree of translation inhibition experienced by stressed cells (Appendix Fig. S4D). Specifically, we hypothesized that the translation of *XBP1*s mRNAs might be hindered by PERK/P-eIF2α-mediated translation inhibition, thereby delaying XBP1s synthesis and downstream transcription of its target genes. In contrast, ATF6f appeared to be relatively less dependent on active translation (Appendix Fig. S4C), as its early mechanism of activation relies on the proteolytic cleavage of pre-existing ATF6 rather than its de novo synthesis (Haze et al, 1999; Ye et al, 2000). In conclusion, SNUPR enabled us to uncover a heterogeneous dynamic of UPR activation among distinct cell types and in response to different stressors, hinting at a potential dependence of UPR responses on intensity and duration of PERK-p-eIF2α-mediated translation inhibition.

### Impact of translation arrest on activation of IRE1α/XBP1s and ATF6 pathways

**Figure 4:**
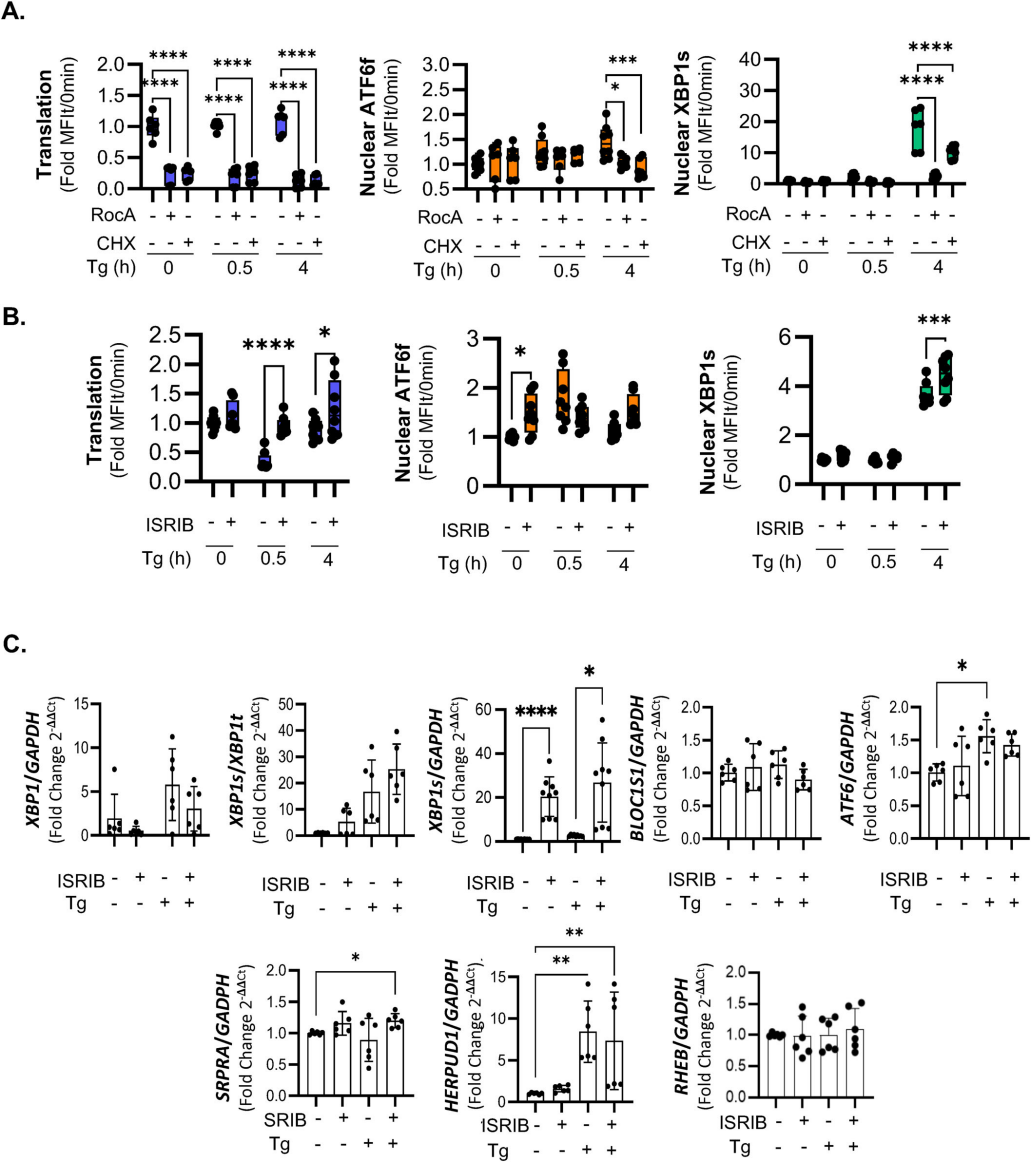
Translation arrest delays the activation of the IRE1/XBP1s and ATF6 pathways. THP-1 and HeLa cells were treated or not for 30 min or 4 h with thapsigargin (Tg, 400 nM) in the presence or absence of different translation inhibitors prior to SNUPR profiling by flow cytometry. (**A**) Translation levels and translocation of ATF6f and XBP1s measured on THP-1 cells after treatment with Tg in combination with Rocaglamide (RocA, 100 nM) or cycloheximide (CHX, 5 µM). (**B**) Translation levels and translocation of ATF6f and XBP1s measured on HeLa cells after treatment with Tg combined with ISRIB (1 µg/mL). Each point corresponds to one replicate of three independent experiments (*n* = 3). Statistical analysis was performed using a two-way ANOVA test. *P < 0.05, **P < 0.01, and ***P < 0.001. For (**A**, **B**), in box plots, the center line indicates the median, box limits indicate the 25th and 75th percentiles, and whiskers indicate the minimum and maximum values. (**C**) HeLa cells were treated 30 min or 4 h with thapsigargin (Tg, 400 nM) in the presence or absence of ISRIB (1 µg/mL) prior to nuclei and RNA isolation for SNUPR profiling and transcriptional analysis of UPR-associated transcripts. (**A**) RT-qPCR analysis of XBP1 splicing as well as relative mRNA levels of XBP1s, XBP1tot, ATF6a, and BLOC1S1 on HeLa cells treated with Tg in combination with ISRIB. Statistical analysis was performed using a two-way ANOVA test. *P < 0.05, **P < 0.01, and ***P < 0.001. Source data are available online for this figure.

To further explore the effect of PERK-mediated translation arrest on nuclear XBP1s and ATF6f, we took advantage of the absence or presence of transient translation inhibition in THP-1 and HeLa cells, respectively. To test our hypothesis, we forced translation inhibition in THP-1 (Fig. 4A) and blocked PERK-dependent translation inhibition in HeLa cells upon ER stress and measured the level of activation of the IRE-1/XBP1s pathway. Although THP-1 and HeLa cells display contrasting UPR dynamics, both cell types express high levels of XBP1s after 4 h of stimulation (Figs. 3 and  4). Co-treatment of THP-1 cells with Tg and the translation inhibitors rocaglamide (RocA) or cycloheximide (CHX) efficiently suppressed translation and reduced nuclear translocation of XBP1s and ATF6f after 4 h of UPR (Fig. 4A). As expected, HeLa cells underwent translation arrest very rapidly within 30 min of Tg exposure (Fig. 4B), although full recovery was observed within 4 h. Pre-treatment of HeLa cells with the ISR inhibitor (ISRIB), a compound known to bypass the inhibitory effect of PERK-dependent eIF2α phosphorylation on translation (Sidrauski et al, 2015), circumvented this transient inhibition (Fig. 4B) and led to significantly increased nuclear XBP1s levels at 4 h post-treatment. ATF6f showed a similar trend of increased nuclear levels at 4 h, but these were not statistically significant. To further investigate, the downstream effects of altering translation levels we measured XBP1s-dependent genes expression, such as *RHEB*, *HERPUD1*, or *SRPRA*, on Tg- and ISRIB-treated HeLa cells and detected no significant differences in mRNA expression levels (Fig. 4C). Translation blockade in HeLa cells upon thapsigargin lasts only 30 min. Higher levels of XBP1s upon thapsigargin and ISRIB treatment suggest that this short inhibition of translation and delay in XBP1s protein expression observed in HeLa cells can be circumvented by recovering translation and is too short to impact the transcription of XBP1s target genes durably.

### SNUPR highlights a cell-type-dependent UPR profile in PBMCs

**Figure 5:**
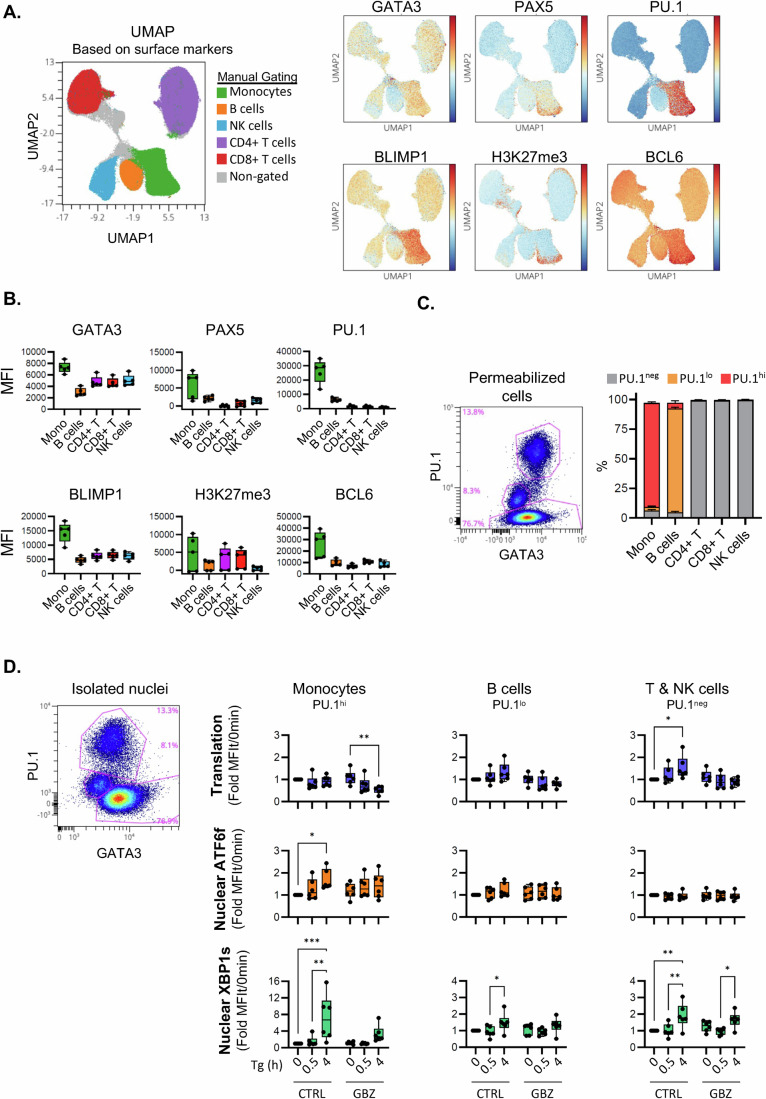
Lineage-associated transcription factor staining and SNUPR profiling on PBMC. Human peripheral blood mononuclear cells (PBMC) from six healthy donors were stained with surface markers (CD3, CD4, CD8, CD19, CD16, HLA-DR), permeabilized, and stained intracellularly for nuclear markers (GATA3, PAX5, PU.1, BLIMP1, H3K27me3, BCL6) to identify cell subsets by FACS. (**A**) UMAP for PBMC was generated by using surface markers only. UMAPs colored either by manually gated immune populations based on surface markers (left); or by nuclear marker expression levels (right panel). (**B**) Quantification of MFI for different nuclear markers on the main immune populations. Each measurement was repeated three times independently (*n* = 3). (**C**) Three gates were defined based on PU.1 and GATA3 expression levels for permeabilized cells. Stacked bars show the distribution of cells in these gates for each immune cell subset. (**D**) SNUPR profiling of PBMC. PBMCs were treated with thapsigargin (Tg, 400 nM) for 30 min or 4 h in the presence or absence of guanabenz (GBZ, 50 µM) prior to nuclear isolation. Nuclei identity was defined based on PU.1 and GATA3 expression (left). Translation levels and nuclear translocation of ATF6f and XBP1s were measured on isolated nuclei by FACS (right). MFI values were normalized to control treated samples. In (**B**, **D**), in box plots, the center line indicates the median, box limits indicate the 25th and 75th percentiles, and whiskers indicate the minimum and maximum values. Each point corresponds to one replicate of three independent experiments (*n* = 3). Statistical analysis was performed using a two-way ANOVA test. *P < 0.05, **P < 0.01, and ***P < 0.001. Source data are available online for this figure.

Given that SNUPR uncovered cell-type and stressor-dependent heterogeneity of UPR pathways activation in cultured cell lines, we next assessed whether this method could resolve UPR branch activation in primary samples. For this, we expanded the capabilities of SNUPR by incorporating intracellular staining of lineage-specific transcription factors as well as global levels of epigenetic marks to enable the identification of distinct cell populations following nuclei isolation. PBMC from healthy donors were stained using a combination of surface and nuclear markers (Fig. 5A,B). Among the nuclear markers examined, we found that PU.1 and GATA3 were sufficient to unequivocally identify monocytes, B cells, and a third cluster of T/NK cells (Fig. 5C). We then analyzed isolated nuclei from PBMC treated with Tg in the presence or absence of guanabenz (GBZ), an inhibitor of eIF2α dephosphorylation that prevents recovery of translation (Tsaytler et al, 2011) (Fig. 5D). SNUPR revealed differential activation of UPR pathways across distinct cellular subsets in response to Tg. In addition, ATF6f was primarily observed in nuclei from monocytes and B cells, whereas XBP1s was predominantly found in monocytes and to a lesser extent, in B and T/NK cell nuclei (Fig. 5D). Furthermore, and concordant with our observations in cell lines, primary monocytes displayed increased XBP1s levels after 4 h of Tg treatment, which were significantly reduced after prolongation of translation inhibition with GBZ (Fig. 5D), indicating that PERK-dependent translation inhibition can modulate IRE-1/XBP1s signaling in primary immune cells.

**Figure EV2:**
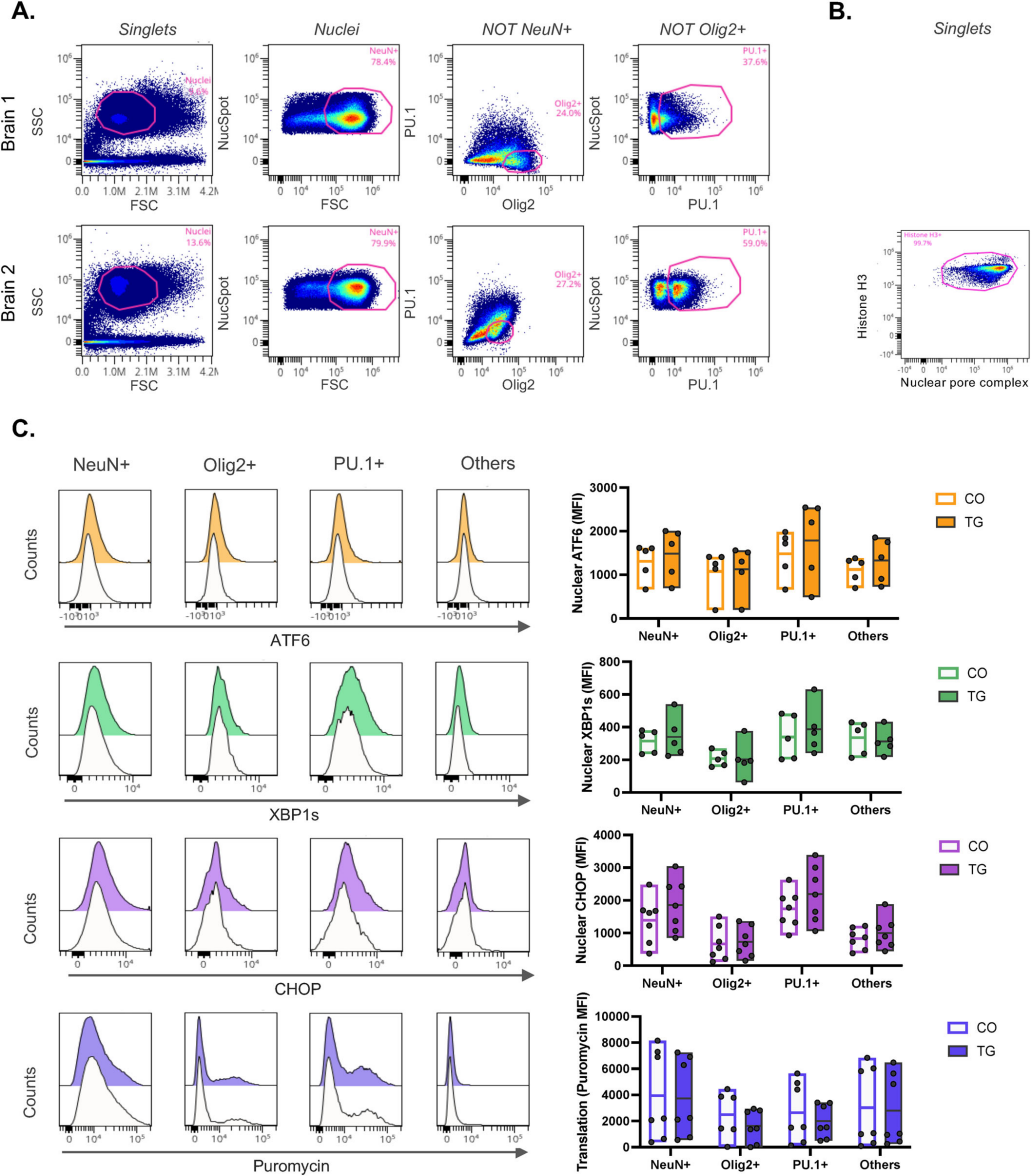
Cell-type-specific nuclear marker staining enables SNUPR profiling of mouse brain cell subsets. Nuclei isolated from mouse brain slices were stained intracellularly for neural cells–specific nuclear markers and analyzed by flow cytometry. Neuronal, oligodendrocyte, and microglial/MDMC nuclei were identified based on NeuN, Olig2, and PU.1 expression, respectively. (**A**) Representative gating strategy for singlet nuclei selection using FSC/SSC and NucSpot staining, followed by identification of NeuN⁺, Olig2⁺, and PU.1⁺ nuclear populations. The data shown are representative of 2 out of 5 independent brain specimens. (**B**) Validation of nuclei identification by co-staining with independent nuclear markers, including total histone H3 and nuclear pore complex proteins. (**C**) SNUPR profiling of cell-type-specific stress responses in brain tissue. Brain slices were treated with thapsigargin (Tg; 400 nM, 2 h) followed by a 40 min puromycin pulse prior to nuclei isolation. Nuclear translocation of ATF6f, XBP1s, and CHOP, as well as translational activity assessed by puromycin incorporation, were quantified by flow cytometry. Box plots show median fluorescence intensity (MFI) values for neuronal (NeuN⁺), oligodendrocyte (Olig2⁺), microglial/MDMC (PU.1⁺), and Other nuclear populations from three independent experiments with technical triplicates (XBP1s, *n* = 2). Source data are available online for this figure.

We next assessed whether SNUPR could be applied to more complex tissues by analyzing nuclei isolated from the mouse brain. Using lineage-associated transcription factors, we identified major neural cell populations, including NeuN+ neurons, Olig2+ oligodendrocyte cells, and PU.1+ microglia/immune-associated cells (Figs. EV2A and EV2B). After 2 h of Tg treatment of brain slices followed by puromycin incubation, nuclei were isolated, fixed, and stained with anti-NeuN, Olig2, PU.1, anti-nuclear antibodies, and UPR markers. To increase further the versatility of SNUPR, we introduce in the assay the monitoring of nuclear CHOP (DDIT3), a pro-apoptotic transcription factor commonly associated with the UPR and PERK-ATF4 pathway. Upon Tg treatment, SNUPR revealed cell-type-specific differences in UPR branch activation, with distinct patterns of nuclear XBP1s, ATF6f across brain cell populations, Olig2 cells were relatively unreactive to Tg, while CHOP and XBP1s were increased in NeuN+ neurons. Protein synthesis is more downregulated in Tg-treated Olig2 cells and microglia than in other neural cells (Fig. EV2C).

Together, these results demonstrate that SNUPR enables cell-type resolved profiling of the three branches of the UPR at the single-nuclei level in both, heterogeneous primary cells and tissues. This differential stress-response dynamics would be masked by other biochemical bulk-based approaches, thus highlighting the application of SNUPR for the study of UPR signaling in complex systems.

### Role of IRE-1/XBP1s signalling in Bortezomib resistance in multiple myeloma cells

Upon chemotherapies, resistant cancer cells can induce or rely on specific transcriptional programs (Neri et al, 2024). We sought to further explore how heterogeneity in UPR activation might impact the chemotherapy response in patients with multiple myeloma. Specifically, we focused on the role of endoplasmic reticulum (ER) stress response in mediating the efficacy of Bortezomib (BTZ) (Velcade, previously PS-341). BTZ is a proteasome inhibitor that is currently included in the first line of treatment against multiple myeloma (MM) and has been reported to induce cytotoxic ER stress (Carlsten et al, 2019).

**Figure EV3:**
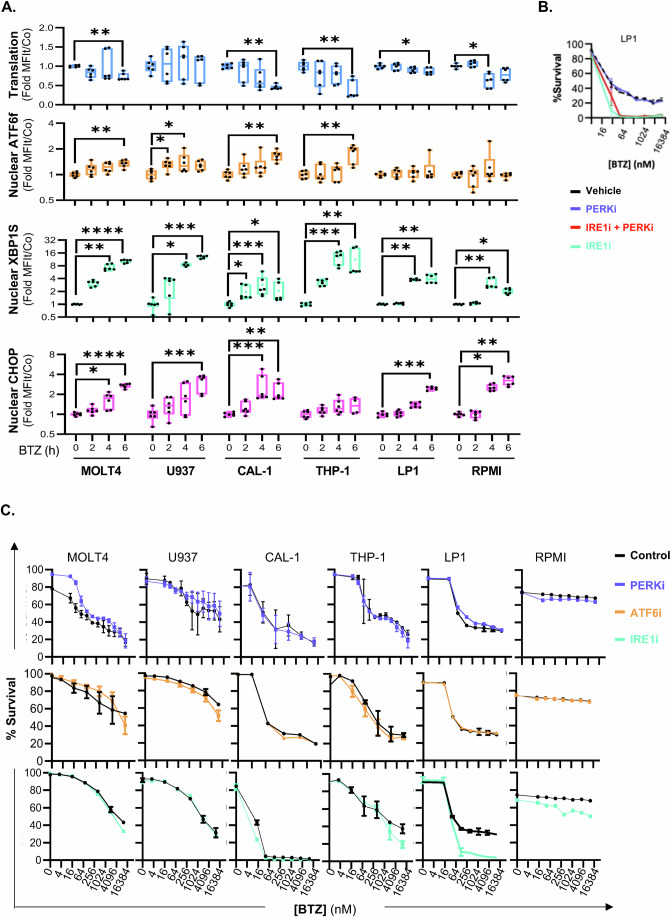
Bortezomib induces UPR activation in leukemic and myeloma cells. Leukemia cell lines and two multiple myeloma (MM) cell lines, LP1 and RPMI, were treated with the proteasome inhibitor bortezomib (BTZ, 100 nM) for 2, 4, and 6 h prior to nuclei isolation and SNUPR profiling. (**A**) SNUPR profiles of translation levels and ATF6f and XBP1s nuclear translocation of cancer cells treated with BTZ for 2 h, 4 h or 6 h. in box plots, the center line indicates the median, box limits indicate the 25th and 75th percentiles, and whiskers indicate the minimum and maximum values. Statistical analysis was performed using the Kruskal–Wallis test for each cell line. *P < 0.05, **P < 0.01, and ***P < 0.001. (**B**) Cell survival analysis of LP1 cells treated 6 h with BTZ in the presence or absence of IRE1α inhibitor 4u8c (10 µM). PERK inhibitor GSK2656157 (100 nM) or a combination of both was measured by flow cytometry. (**C**) Survival analysis of leukemia and multiple myeloma cells treated with a gradient concentration of BTZ in combination with a PERK inhibitor (GSK2656157, 100 nM, blue). ATF6 inhibitor (Ceapin A7, 6 µM orange) or IRE1α inhibitor (4u8, green) for 24 h. All analyses were performed by flow cytometry measurements of cell viability dye signals. Source data are available online for this figure.

We used SNUPR to profile the UPR response to BTZ treatment across several leukemia and MM cell lines over time (0-6 h). BTZ treatment resulted in decreased protein synthesis in most cell lines, with the notable exception of U937 and to a lesser extent LP1 cells (Fig. EV3A). This contrasted with the results observed upon Tg treatment (Fig. 3). Although variable in intensity, XBP1s translocation was consistent throughout cell lines; ATF6 translocation, however, was only detected on the leukemia cell lines but not in RPMI nor LP1 MM cell lines (Fig. EV3A). CHOP induction by BTZ was induced in most cell lines, except for THP1, where only a moderate induction trend without statistical significance was noted. Taken together, these observations corroborate a BTZ-mediated activation of the UPR.

**Figure 6:**
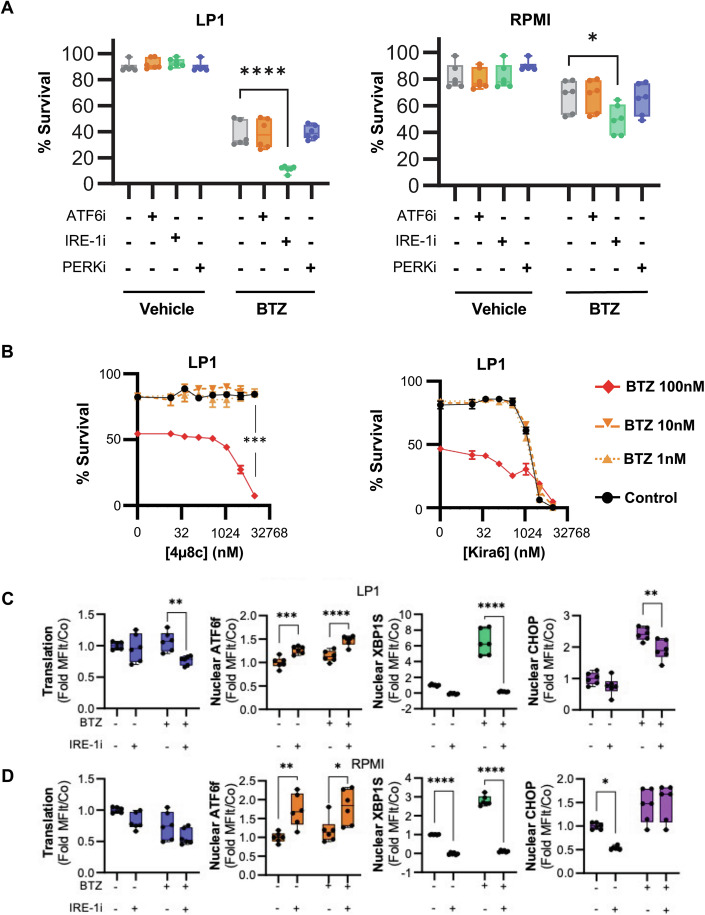
IRE1α contributes to bortezomib resistance in multiple myeloma cell lines. Cell survival analysis was performed on MM cells to assess potential synergic effects between BTZ and UPR inhibitors. (**A**) Flow cytometry analysis of survival of LP1 and RPMI MM cell lines after treatment with BTZ in combination with the ATF6 inhibitor (Ceapin A7, 6uM), IRE1α inhibitor (4u8c, 10 µM), or PERK inhibitor (GSK2656157, 100 nM) for 24 h. (**B**) Cell survival analysis by flow cytometry of LP1 cells treated for 24 h with different concentrations of BTZ (1 nM, 10 nM, 100 nM) in the presence of different concentrations of IRE1α inhibitors 4µ8c (left) and Kira6 (right). (**C**, **D**) SNUPR profiling of (**C**) LP1 and (**D**) RPMI cell lines treated with BTZ 100 nM for 4 h in the presence or absence of IRE1α inhibitor 4u8c (10 μM). (**A**, **C**, **D**) In box plots, the center line indicates the median, box limits indicate the 25th and 75th percentiles, and whiskers indicate the minimum and maximum values. Statistical analysis was performed using a two-way ANOVA test. *P < 0.05, **P < 0.01, and ***P < 0.001. Source data are available online for this figure.

To assess whether activation of any of the specific UPR branches played an essential role in BTZ toxicity, we quantified BTZ-induced cytotoxicity after 24 h of treatment in the presence of specific pharmacological inhibitors for each of the three ER stress sensors. Inhibition of PERK, IRE1α, and ATF6 pathways did not rescue BTZ-induced cell death (Fig. EV3B,C). Moreover, in LP1 MM cells, a substantial fraction of cells (~20–40% of the population) remained viable even at the highest BTZ concentrations tested (Figs. 6A and  EV3B,C). Strikingly, co-treatment of BTZ with the IRE1α inhibitor selectively reduced the survival of these BTZ-tolerant cells, indicating that their persistence depends on IRE1α activity (Figs. 6A and  EV3B,C). Inhibition of IRE1α with the RNAse inhibitor 4µ8c alone did not show any toxicity in absence of BTZ, while higher doses of the IRE1α kinase inhibitor Kira6 decreased the overall survival of MM cells in all conditions (Fig. 6B). Given the differences of specificity of the two compounds and knowing that Kira6 also inhibits the p38 and ERK MAP kinase (Ghosh et al, 2014; Tang et al, 2022), we suspect an IRE1-independent off-target effect in Kira6 cytotoxicity at these higher concentrations. Overall, these results suggest that activating the UPR is not essential for BTZ to mediate cytotoxicity, but in contrast, in multiple myeloma cells, IRE1α activity contributes to resistance to Bortezomib.

To further characterize the UPR mechanism of resistance to BTZ, we performed SNUPR and transcript analysis on LP1 and RPMI cell lines treated with BTZ in the presence or absence of 4µ8C (IRE-1i) (Fig. 6C,D; Appendix Fig. S5A). Our results confirmed that IRE1α inhibition decreases basal level of nuclear XBP1s, together with *XBP1* mRNA splicing and expression of XBP1s-regulated genes such as *HERPUD1* or *DNAJB9* (Appendix Fig. S5A). In contrast, ATF6f translocation was increased after IRE1α inhibition, but transcription of *ATF6a* mRNA and of its target *HSPA5* remained stable across all conditions (Fig. 6C,D; Appendix Fig. S5A), suggesting that the modest effect of IRE1i on ATF6f translocation has only limited effect on downstream ATF6-dependent transcription and confirming that IRE-1/XBP1s is the only branch of the UPR that contributes to increase resistance to BTZ of MM cells.

### Pharmacological UPR modulation modestly contributes to overturn resistance to BTZ in multiple myeloma cell lines

Upon our findings of the significant impact of translation inhibition on the IRE-1/XBP1s axis and the involvement of this pathway in resisting BTZ treatment, we harnessed these results by investigating whether UPR-inducing drugs could synergize with BTZ. A reinforced UPR could further drive PERK activation and eIF2α phosphorylation levels to reduce XBP1s translation and thus undermine BTZ resistance by dwarfing the consequences of IRE1α signaling (Fig. 4).

**Figure 7:**
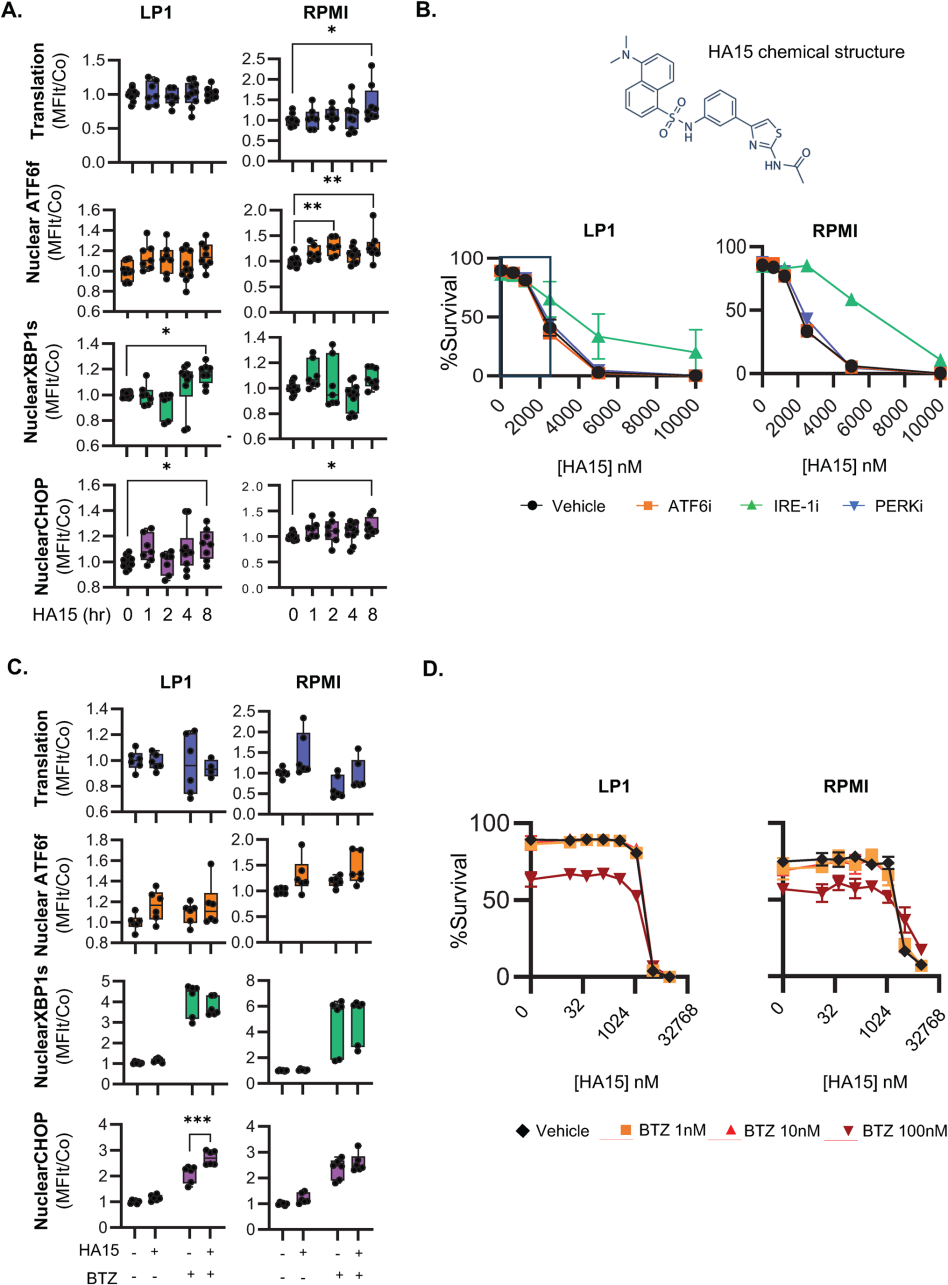
BIP inhibition by HA15 induces cell death in BTZ-resistant and non-resistant multiple myeloma lines. (**A**) SNUPR analysis of LP1 and RPMI cells treated or not with HA15 (5 µM) for the indicated times. Statistical analysis was performed using the Kruskal–Wallis test for each cell line. *P < 0.05, **P < 0.01, and ***P < 0.001. (**B**) Flow cytometry analysis of survival of LP1 and RPMI cells treated with or without ATF6, IRE1α, or PERK inhibitors in combination with increasing concentrations of HA15 for 24 h (0, 625 nM, 1250 nM, 2500, 5000, and 10000 nM). (**C**) SNUPR profiling of LP1 and RPMI cells treated 4 h with BTZ in presence or absence of HA15. Statistical analysis was performed using a two-way ANOVA test. (**D**) Flow cytometry analysis of survival of LP1 and RPMI cells treated with different concentrations of BTZ (1 nM, 10 nM, and 100 nM) in combination with increasing concentrations of HA15 for 24 h. Statistical analysis was performed using a two-way ANOVA test. *P < 0.05, **P < 0.01, and ***P < 0.001. (**A**, **C**) In box plots, the center line indicates the median, box limits indicate the 25th and 75th percentiles, and whiskers indicate the minimum and maximum values. Source data are available online for this figure.

We tested HA15, a specific inhibitor of the ATP-dependent chaperone BiP/HSPA5, which was reported to exert its activity by inducing a lethal ER stress, particularly in melanoma cells (Cerezo et al, 2016; Cerezo and Rocchi, 2017). We performed SNUPR on LP1 and RPMI cell lines treated with HA-15 over time (Fig. 7A). Eight hours of HA15 treatment triggered modest XBP1s translocation in both cell lines, as well as some expected CHOP synthesis and translocation (Cerezo and Rocchi, 2017). Despite CHOP activation, HA15 had no impact on translation levels in LP1 and even slightly elevated them after 8 h in RPMI cells. We nevertheless tested the toxicity of HA-15 in MM cells, in the presence of the different UPR inhibitors (Fig. 7B). We found that HA15 efficiently killed both cell lines at concentrations above 5 µM, but surprisingly, inhibition of the IRE1α pathway, and not of the other UPR branches, had a protective effect and reduced the efficacy of the HSPA5 inhibiting compound. Thus, contrasting with BTZ, HA15 seems to rely on IRE1α activation to kill MM cells, and inhibition of this specific UPR branch leads to enhanced survival. These contrasting results called for further assessment of the combinatorial effect of exposing MM cells to HA15 together with BTZ. Co-treatment with both compounds had a modest enhancing effect on the activation of the UPR monitored with SNUPR (Fig. 7C). Levels of translation were not reduced and were accompanied by an elevation of CHOP nuclear levels and equivalent translocation of ATF6f and XBP1s (Fig. 7C). When examining cytotoxicity, no synergy in the killing of MM cells could be observed by gradually increasing drug quantities (Fig. 7D), while HA15 killed efficiently BTZ-resistant MM cells at micromolar concentrations. Interestingly, although the two drugs induced IRE1α activation, it had opposite consequences for the cells: IRE1α activity mediating survival in BTZ, while promoting cell death in response to HA15. Taken together, these findings suggest that the molecular mechanisms by BIP inhibition and proteasome inhibitors induce cytotoxicity is different in MM and involves opposite roles of IRE1α (Figs. 6 and  7).

### UPR gene signatures and their clinical relevance in multiple myeloma

**Figure 8:**
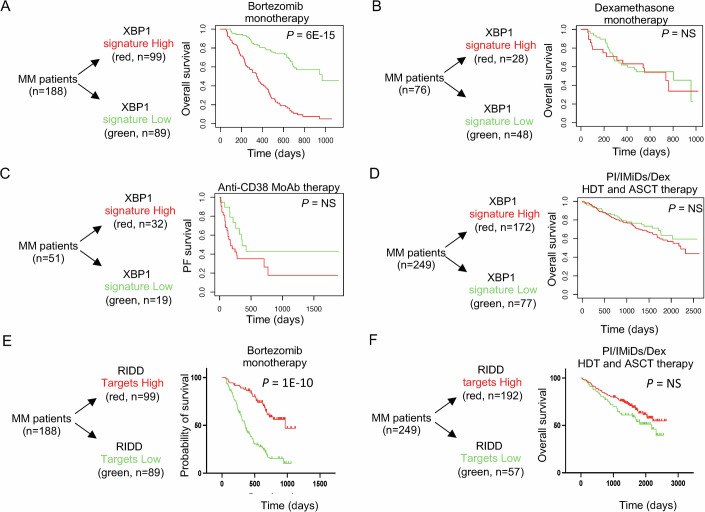
XBP1-dependent and RIDD target gene signature is associated with outcome in multiple myeloma patients treated with bortezomib, but not in patients receiving other therapies. (**A**–**D**) Multiple myeloma (MM) patients were stratified according to the expression of an XBP1 target gene signature, and overall survival was analyzed using Kaplan–Meier estimates. The prognostic value of the XBP1 signature was evaluated in cohorts treated with (**A**) bortezomib (BTZ) monotherapy, (**B**) dexamethasone monotherapy, (**C**) anti-CD38 monoclonal antibody therapy, and (**D**) combination therapy with proteasome inhibitors (PI), immunomodulatory drugs (IMiDs), and dexamethasone followed by high-dose chemotherapy (HDT) and autologous stem cell transplantation (ASCT). (**E**) MM patients stratified by the mRNA expression of RIDD targets in patients treated with Bortezomib or (**F**) with combination therapy with proteasome inhibitors (PI), immunomodulatory drugs (IMiDs), and dexamethasone followed by high-dose chemotherapy (HDT) and autologous stem cell transplantation (ASCT). For (**A**–**F**), statistical analysis was performed using the Log-rank test.

We have shown that the IRE-1/XBP1s pathway is associated with resistance to BTZ treatment in human MM cell lines in vitro. To test the clinical relevance of these findings, we investigated whether the presence of XBP1 transcriptional signatures correlated with resistance to BTZ and/or survival in treated MM patients. For this analysis, we focused on the Mulligan cohort of relapsed MM patients who were treated exclusively with BTZ (Mulligan et al, 2007). Patients were stratified based on the expression levels of gene signature consisting in a set of XBP1 target genes prior to treatment, and survival probability was monitored over time (Fig. 8). Notably, high mRNA expression levels of most genes of the IRE-1α/XBP1s pathway significantly correlated with a poor prognosis (Fig. 8A). To determine whether the XBP1 gene signature is specifically predictive of poor prognosis following BTZ treatment, or whether it serves as a marker of adverse outcomes independently of the treatment, we conducted an analysis using three different MM patient cohorts of patients treated with Dexamethasone, with anti-CD38 as monotherapy, or receiving a combination of immunomodulatory drugs (IMiDs), dexamethasone (Dex), and high-dose melphalan followed by autologous stem cell transplantation (Fig. 8B–D). Strikingly, the XBP1 gene signature had only a strong predictive value in patients treated with Bortezomib, but not in patients treated with other treatments.

**Figure EV4:**
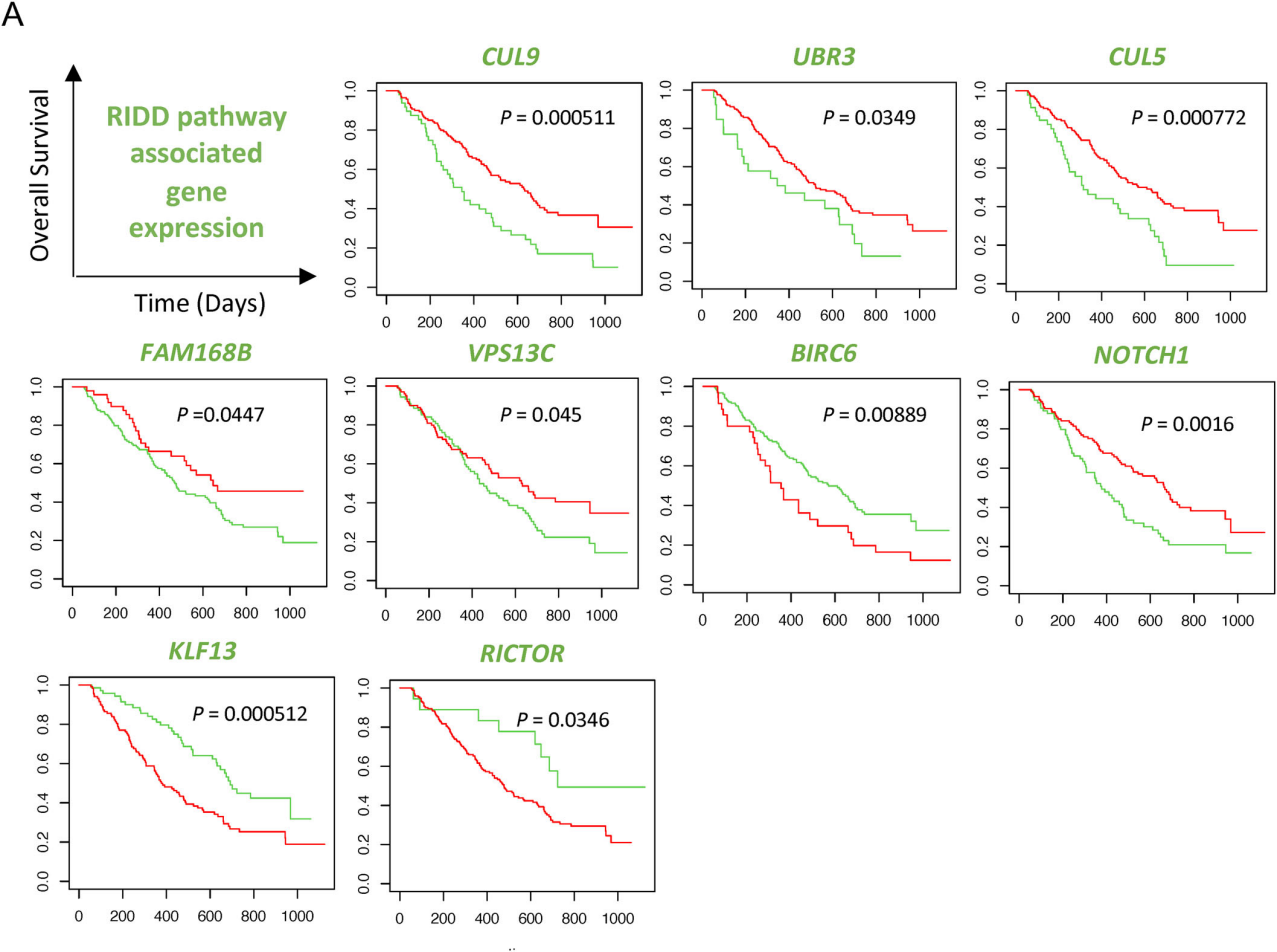
RIDD gene targets expression associated with a favorable outcome in multiple myeloma patients treated with bortezomib. (**A**) Multiple myeloma (MM) patients from the Mulligan cohort treated with bortezomib (BTZ) monotherapy were stratified according to the expression level of a curated set of IRE1α-dependent RIDD target genes. Kaplan–Meier survival curves for individual RIDD-associated genes showing significant prognostic value in the Mulligan BTZ monotherapy cohort. For each gene, patients were stratified based on low expression levels (green curves) and high (red curves).

We then explored whether RIDD activity also contributes to survival outcome in BTZ-treated MM. We generated a curated RIDD target gene set and evaluated its prognostic value in the Mulligan cohort. From a list of 35 gene targets of the RIDD pathway, 9 confirmed RIDD targets (Appendix Table S1) were expressed and significantly associated with survival, with the majority (6/9) correlating with better outcome when highly expressed. Because RIDD activity leads to degradation of its target transcripts, high expression of RIDD target genes reflects reduced RIDD activity and thus lower IRE1α activity. Accordingly, a high RIDD score (high expression of RIDD targets) was associated with improved survival in BTZ-treated patients (Figs. 8E and EV4A). However, and similar to what we observed with XBP1s target gene signature, the RIDD score did not significantly stratify survival in an independent cohort of newly diagnosed MM patients treated with high-dose chemotherapy and autologous stem cell transplantation (Fig. 8F).

Together, these analyses reinforce the potential relevance of the IRE-1/XBP1 pathway in BTZ resistance and the potential therapeutic value of its modulation.

## Discussion

Under ER stress, cells activate the unfolded protein response (UPR) to restore proteostasis or trigger apoptosis, and tumor cells are particularly dependent on this pathway to survive hostile microenvironments and chemotherapy. SNUPR combines nuclear extraction, flow cytometry, and translation measurements to quantify branch-specific UPR activation at single-nucleus resolution, providing higher contrast than permeabilized whole-cell assays and revealing marked cell type- and context-dependent heterogeneity that is hidden in bulk analyses. By incorporating lineage-associated transcription factors, SNUPR can resolve UPR wiring in primary PBMCs and brain tissue.

Using SNUPR with classical ER stressors, thapsigargin and tunicamycin were found to elicit distinct UPR branch dynamics and kinetics across closely related cell types, underscoring that these agents are not interchangeable despite both causing misfolded protein accumulation in the ER lumen. The data further support a functional hierarchy between IRE1/XBP1s and PERK, in which PERK-mediated translation arrest limits XBP1s protein accumulation despite efficient IRE1-dependent mRNA splicing. The three canonical branches of the unfolded protein response (UPR), IRE1, PERK, and ATF6, do not function in isolation but instead engage in intricate crosstalk that shapes the overall cellular response to endoplasmic reticulum (ER) stress. IRE1α-XBP1s activity has been shown to promote PERK expression during chronic ER stress, establishing a reciprocal regulatory loop between these two branches (Ong et al, 2024). In addition, mutual inhibition and feedback loops involving factors such as p58IPK and GADD34 help fine-tune the sensitivity and timing of each pathway’s activation, ensuring an integrated and context-dependent UPR outcome (Márton et al, 2022; Hwang and Qi, 2018). This dynamic interplay is essential for determining cell fate decisions, as positive feedback between IRE1α and PERK can reinforce the commitment to apoptosis under unresolved stress, while regulatory crosstalk supports adaptive responses when stress is transient or moderate. Also, in line with this integrated view of UPR regulation, we observed conditions in which modest ATF6 nuclear levels were accompanied by a pronounced decrease in XBP1s without a corresponding induction of ATF6 target genes. This suggests that simultaneous measurement of XBP1s and ATF6 within the same cell provides a more accurate prediction of transcriptional outcomes than measurement of ATF6 alone, reflecting the overlapping and cooperative regulation of target genes by these two transcription factors. Moreover, GADD34/PP1c-driven dephosphorylation of p-eIF2α controls translation recovery and thereby the timing and amplitude of XBP1s and ATF6 activation. Given the availability of pharmacological modulators of the integrated stress response, targeting PERK, eIF2α, or GADD34 offers an indirect means to tune XBP1s signaling and UPR branch balance for therapeutic purposes.

In multiple myeloma (MM), Bortezomib (BTZ) is a cornerstone therapy that induces apoptosis at least in part through ER-stress signaling, yet resistance and relapse remain frequent and are only partially explained by mutations in the proteasome subunit PSMB5 (Ri et al, 2010). SNUPR and transcriptomic analyses identify an IRE1/XBP1s-driven gene signature associated with poor outcome in BTZ-treated patients, whereas PERK/ATF4-dominated profiles are less predictive, indicating that overactivation rather than loss of XBP1s function can underlie resistance. Consistent with this, nuclear XBP1s protein activity is a better correlate of BTZ responsiveness than bulk sXBP1 mRNA levels, highlighting the added value of single-nucleus, branch-resolved measurements over conventional bulk assays. Combining BTZ with an IRE1α inhibitor (4µ8C) efficiently eliminates proteasome-inhibition-resistant MM cells, reinforcing the notion that excessive IRE1/XBP1s signaling is a therapeutically actionable vulnerability.

A previous study reported that BTZ-treated MM patients with a bad prognosis exhibit lower levels of spliced *XBP1* mRNA, measured through bulk qPCR (Borjan et al, 2019). In contrast, our analysis and data show that BTZ-treated MM with a bad prognosis (*P* < 0.001) is observed in patients with increased expression of XBP1 transcriptional targets, and also in patients with low mRNA levels of IRE1-RIDD targets (*P* < 0.001). These are in line with our experiments demonstrating the requirement of IRE1 activity for the survival of BTZ-treated MM cells. These differences may come from methodological differences, as bulk mRNA assays capture total XBP1s transcript abundance but do not reflect nuclear protein localization or transcriptional activity, whereas SNUPR directly measures XBP1s in the nucleus, and the signatures are based on expression of multiple XBP1s targets. Our results therefore suggest that nuclear XBP1s activity is a reliable predictor of BTZ responsiveness. Furthermore, this cited study focused solely on XBP1s, while SNUPR profiles simultaneously all the 3 UPR branches, allowing a more integrative analysis of pathway crosstalk.

Stratification of BTZ-treated patients using an XBP1s-dependent gene signature delineates a subgroup with high survival rates comparable to those achieved with standard combination regimens, suggesting that such biomarkers could guide the use of BTZ monotherapy or reduced-intensity combinations in line with personalized-medicine goals. Comparison of SNUPR profiles induced by BTZ and by the BiP/HSPA5 inhibitor HA15 further shows that different ER-stress inducers engage distinct UPR dynamics and rely on opposite sides of the IRE1α axis for cytotoxicity, implying that the efficacy and mechanisms of UPR-targeting drugs are highly context- and tumor-type specific.

Together, these findings position the IRE1/XBP1s pathway as a central determinant of MM responses to chemotherapy and illustrate how SNUPR can dissect UPR heterogeneity and pathway crosstalk to inform rational design of combinatorial treatments and prediction of patient outcome.

## Methods

**Table:** Reagents and tools table

Reagent/resource	Reference or source	Identifier
**Antibodies**		
Anti-human ATF6 A350 (2358 C)	R&D Biosystems	Cat#IC71527U
Anti-human XBP-1S PE (Clone Q3-695)	BD Bioscience	Cat#15802109
Anti-puromycilated peptides A647 (Clone R4743L-E8)	GammaOmics	Cat#GO005AF647050
Anti-human CHOP A488 (B-3)	Santa Cruz	Cat#sc-7351
Anti-human anti-nuclear pore (MAb414)	Biolegend	Cat#682203
Anti-human Calnexin (AF18)	Thermo Scientific	Cat#MA3027
ProLong™ Gold Antifade Mountant with DNA Stain DAP	invitrogen	Cat# P36931
Anti-human SDHA	Abcam	Cat#ab14715
Anti-human PERK	Cell Signaling	Cat# 3192S
Anti-human GRASP55	Proteintech	Cat#10598-1-AP
Anti-human βActin (AC-15)	MERCK	Cat# A5441
Anti-human EIF2Bα	Invitrogen	Cat# PA5-28992
Anti-human HDAC6 (H-300)	Santa Cruz	Cat#SC-11420
Anti-human HDAC1 (10E2)	Cell Signaling	Cat#5356S
Anti-mouse CD11b	BD Bioscience	Cat#562287
Anti-mouse CD3 BV421 (Clone: 145-2C11)	Biolegend	Cat#BLE100335
Anti-mouse Ly6C BV711 (Clone: HK1.4)	Biolegend	Cat#128037
Anti-mouse NK1.1 BV510 (Clone PK136)	Biolegend	Cat#108373
Anti-mouse CD4 APC-eFluor 780 (Clone: RM4-5)	eBioscience	Cat#47-0042-82
Anti-mouse MHC II BUV805 (Clone M5/114.15.2)	BD Bioscience	Cat#748844
Anti-human IRE-1	Cell Signaling	Cat# 3294S
Anti-human ATF6	R&D Biosystems	Cat#IC71527U
Anti-NeuN Alexa Fluor 488 (Clone: A60)	Sigma	MAB377X
Anti-Olig2 Alexa Fluor 594 (Clone: EPR2673)	Abcam	Ab300729
Anti-PU.1 PE-Cy7 (Clone : 7C6B05)	Biolegend	658013
NucSpot 750/780	Biotium	41038
LIVE/DEAD™ Fixable Aqua Dead Cell	Invitrogen	Cat#L34957
**Biological samples**		
Murine blood C57BL/6J	The Jackson Laboratory	Stock 000664
**Chemicals, peptides, and recombinant Proteins**		
ß-mercaptoethanol	VWR	Cat#0482-100 ML
Cycloheximide (CHX)	Merck	Cat#01810-1 G
Dimethyl sulfoxide (DMSO)	Merck	Cat#D8418-100ML
Harringtonine	Abcam	Cat#ab141941
4µ8c	Sigma-Aldrich	Cat#SML0949
Ceapin A7	Sigma-Aldrich	Cat#SML2330
GSK2656157	Sigma-Aldrich	Cat#5046510001
Bortezomib	Sigma-Aldrich	Cat#5043140001
Rocaglamide	Sigma-Aldrich	Cat#SML0656
Thapsigargin	Sigma-Aldrich	Cat#T7458
Tunicamycin	Sigma-Aldrich	Cat#SML1287
Cycloheximide	Sigma-Aldrich	Cat#108-91-8
Puromycin	Merck	Cat#P7255
**Critical commercial assays**		
Brilliant Stain Buffer Plus	BD Bioscience	Cat#566385
Fixation/Permeabilization Solution Kit	BD Bioscience	Cat#554714
Foxp3/Transcription Factor Staining Buffer Set	eBioscience	Cat#00-5323-00
LIVE/DEAD™ Fixable Aqua Dead Cell Stain (BV510)	Thermo Fisher	Cat#L34957
Zombie UV™ Fixable Viability kit	Biolegend	Cat#423107
**Experimental models: cell lines**		
THP-1	ATCC	TIB-202
MOLT-4	ATCC	CRL-1582
CAL-1	Provided by Maeda et al, 2005	CVCL_5G46
HeLa	ATCC	CRM-CCL-2
HEK293T	ATCC	CRL-3216
U937	ATCC	CRL-1593.2
Kasumi-1	ATCC	CRL-2724
LP1	Provided by J. Moreaux	CVCL_0012
RPMI 8226	Provided by J. Moreaux	CCL-155
**Deposited data**		
**Experimental models: organisms/strains**		
Mouse: C57BL/6J	The Jackson Laboratory	Stock 000664
FlowJo	Treestar	V10.8.1
7500Software	Applied Biosystem	V2.3
FiJi-ImaJ	System Software	Win64
Prism9	GraphPad	V9.0
Xenabrowser	UC Santa Cruz	https://xenabrowser.net/
**ODN primers**		
Target	Forward (5‘ to 3’)	Reverse (5‘ to 3’)
GAPDH	CATGGCACCGTCAAGGCTG	GACGTACTCAGCGCCAGCAT
XBP1tot	CCCTCCAGAACATCT CCCCAT	ACATGACTGGGTCCAAGTTGT
XBP1S	GCTGAGTCCGCAGCAGGTG	CTGGGTCCAAGTTGTCCAGAATGC
XBP1u	ACATGACTGGGTCCAAGTTGT	CTGGGTCCAAGTTGTCCAGAATGC
RHEB	ACCGGTCTGTGGGGAAATCC	CTTGCCCGGCTGTGTCTACAA
HSPA5	GAACGTCTGATTGGCGATGCCG	GCTGCACAGACGGGTCATTC
PPP1R15A (GADD34)	TCTGGTAGAAGCTGGCCTGG	CCTCCACTGTCTTCAGCCTCC
DDIT3 (CHOP)	AAGATGAGCGGGTGGCAGC	GGTGCTGCTTTCAGGTGTGG
SRPR	GGCTTGTGCTCTGGTGCTTC	GTGAGTGCCTCATGGGTGAAGG
BLOC1S1	GAACACCAGGCCAAGCAGAATG	TGGGCCACACCCACATTGAG
IFNβ	GGAATCCAAGCAAGTTGTAGCTC	ATGACCAACAAGTGTCTCCTCC
CALR	AAGGAGCAGTTTCTGGACGGAG	GAACTTGCCGGAACTGAGAACG
DNAJB9	GCCATGAAGTACCACCCTGACA	CGCGAGTGAAGAGGAGTCACAG
TRAIL-R2	CAGAAGCTCACAACGACCTGGG	GTTGACACCTGTTGGCTCTGC
HERPUD	ATGCCAGAAATCAACGCCAAGGTG	GGTTCCGAAGAACTTCCCTTTGCC
IL-6	CCTCCAGAACAGATTTGAGA	GATTCTTTGCCTTTTTCTGC
DNAJC3	GGAGAGGATTTGCCACTGCTTTTC	TCGTCGGTTCCTCCTTACCTCC
DGAT2	ATTGCTGGCTCATCGCTGT	GGGAAAGTAGTCTCGAAAGTAGC
PRDM1	AACTTCTTGTGTGGTATTGTCGG	CAGTGCGTTGCTTTAGAC
RICTOR	TCCAAAGACTCGACAGTATGTGC	GGCTAGAAATCGTGC TTCTCTG
IRF4	GCTGATCGACCAGATCGACAG	CGGTTGTAGTCCTGCTTGC
ITGB2	AAGTGACGCTTTACCTGCGAC	AAGCATGGAGTAGGAGAGGTC
IL12A	ATGGCCGTCTGCCTTAGTAGT	AGCTTTGCATTCATGGTCTTG
EIF2AK3	TCCGGTTCCTTGGTGTCATCC	GCTTTCACGGTCTTGGTCCCA
ATF6A	TCGTCGGTTCCTCCTTACCTCC	TGACTCAGGGATGGTGCTGAC

### Nuclear extraction

Prior to nuclei extraction, the cells were stained with Aqua Dead™ from Invitrogen for 20 min on ice. Nuclei were extracted using EZ Prep (Sigma-Aldrich N3408) according to the commercial indication. Briefly, cells were harvested in 15-mL tubes, washed with ice-cold PBS prior to nuclei extraction, and centrifuged at 400 × *g* for 5 min at 4 °C. The supernatant was removed, and the cells were resuspended with 1.5 mL of EZ. Nuclear suspensions were incubated on ice for 5 min, pelleted at 500 × *g* for 5 min at 4 °C. Nuclei were resuspended in 1.5 mL EZ lysis buffer, incubated on ice for 5 min, and pelleted before resuspension in 200 µL of PBS 2% PFA for 20 min on ice. Nuclei were pelleted at 500 × *g* for 5 min at 4 °C and resuspended in the storage solution and conserved at −80 °C until permeabilization steps.

For brain nuclear extraction, we followed and adapted the protocol based on Nott et al (Nott et al, 2021). Briefly, brain slices were mechanically dissociated and washed with ice-cold PBS prior to nuclear extraction by centrifuging at 300 × *g* for 10 min at 4 °C. The supernatant was removed, and pellet was resuspended in 3 mL of EZ buffer. Samples were incubated on ice for 5 min, pelleted at 500 × *g* for 5 min at 4 °C, and then the process was repeated: nuclei were resuspended in 3 mL EZ buffer, incubated on ice for 5 min, and pelleted before resuspension in 2 mL of PBS. Nuclear suspensions were then passed through a 35-μm cell strainer and washed with PBS once before resuspension in Fixation/Permeabilization solution to continue with flow cytometry staining.

### Intracellular and nuclear staining for flow cytometry

Cells or nuclei were washed in cold PBS, then fixed and permeabilized using FOXP3 fixation and permeabilization buffer (Thermofisher eBioscience) following manufacturer instructions. Cells and nuclei were blocked 10 min at 4 °C in blockage solution (Permeabilization buffer 2% FCS) and stained with the following antibodies for 1 h at 4 °C in the dark (Reagents and Tools Table). After incubation, nuclei were washed in FACS buffer and passed through a nylon mesh prior to flow cytometry analysis, on Canto II, LSR II UV BD cytometers and 5 L Cytek Aurora spectral cytometer. The list of antibodies used can be found in the Reagents and Tools Table.

### Chemicals

4µ8c (SML0949), Ceapin A7 (SML2330), Bortezomib (5043140001), GSK2656157 (5046510001), rocaglamide (SML0656), and thapsigargin (T7458, 400 nM), tunicamycin (SML1287, 100 nM), KIRA6, cycloheximide were purchased from Sigma-Aldrich, and harringtonine from ABCAM (ab141941).

### Cell lines and culture

THP1, MOLT4, CAL-1, and B-EBV cell lines were cultured in RPMI1640. HeLa and HEK293T were grown in DMEM. All growth media have been supplemented with 10% FCS (Biosera). In addition, CAL-1 was grown in the presence of 10 mM Hepes, 1 mM of sodium pyruvate, 1× of glutamax, 1× non-essential amino acids provided by Gibco. For THP-1, MOLT4 CAL-1, and B-EBV, the percentage of FBS was decreased to 1%, 16 h prior to thapsigargin (Tg) and tunicamycin (Tm) treatments. Cells were regularly tested for mycoplasma. Adherent cells were first trypsinized, then washed with ice-cold PBS prior to nuclei extraction. Whereas suspension cells were directly harvested and washed in ice-cold PBS prior to viability staining and nuclei extraction.

### Animals and ethics statement

All mouse procedures were conducted in accordance with the European Directive 2010/63/EU and relevant French regulations for animal experimentation. Mice were housed in the specific pathogen-free animal facility of the Centre d’Immunologie de Marseille-Luminy (CIML), with controlled temperature and humidity, a 12 h light/12 h dark cycle, and food and water ad libitum. Mouse brains were obtained as surplus material from animals euthanized for purposes unrelated to this study (i.e., meningeal immunity studies). The animal procedures were reviewed and approved by the local institutional ethics committee and conducted under authorization from the French Ministry of Higher Education and Research. Animals were euthanized by isoflurane anesthesia followed by cervical dislocation, in accordance with institutional guidelines, and brains were rapidly collected for ex vivo slice preparation.

### Mouse brain slices

Brains were collected immediately after euthanasia and transferred into ice-cold [cutting solution: e.g., HBSS. Whole brains were embedded in 4% low-melting agarose and sectioned coronally at 250 µm using a vibratome (Leica VT1000S). Slices were allowed to recover for 1 h at 37 °C in culture medium under carbogenation in 24-well plates (1 mL per well, 1 slice per well). Slices were then treated with thapsigargin (400 nM) for 2 h, followed by a 40 min puromycin pulse before being placed on ice and processed for nuclei isolation as described below.

### Quantitative PCR

Total RNA was extracted from cells using the RNeasy Mini Kit (QIAGEN). cDNA was synthesized using the Superscript II Reverse Transcriptase (Invitrogen), and quantitative PCR was performed with ONEGreen FAST qPCR Premix provided by Ozyme using 10 µM of each specific primer on a 7500 Fast RealPCR system (Applied Biosystems). cDNA concentration in each sample was normalized to GAPDH expression. The primers used for gene amplification are depicted in the Appendix Table S1.

### Immunoblotting

Cells were lysed in Triton buffer (20 mM Tris, pH 7.6, 10 mM NaCl, 1.5 mM MgCl2; 1% Triton) supplemented with Complete Mini Protease Inhibitor Mixture Tablets (Roche), NaF (Ser/Thr and acidic phosphatase inhibitor), Na_3_VO_4_ (Tyr and alkaline phosphatase inhibitor), and MG132 (proteasome inhibitor). Protein quantification was performed using the BCA Protein Assay (Pierce). Around 20 μg of soluble proteins were run in 10% acrylamide gels, and for the immunoblot, the concentration and time of incubation had to be optimized for each individual antibody. The list of antibodies used can be found in the Reagents and Tools Table.

### Immunofluorescence confocal microscopy

For immunofluorescence confocal microscopy, cells were seeded on coverslips, fixed with 3.3% PFA, and permeabilized 5 min with 0.1% Triton X-100 or 0.05% Saponin. Before staining, samples were incubated with blocking buffer (PBS 1X, 5% FCS, 1% Glycine). Antibodies were added to the samples in a wet chamber for 1 h at RT or overnight at 4 °C. Coverslips were washed in PBS three times before secondary staining. Samples were then washed in PBS and pure water prior to glass mounting in ProLong™ Glass Antifade Mountant with nucleic stain (Invitrogen P36980).

### Gene signature generation and MM patient stratification

For prognostic survival analyses, we followed the REMARK recommendations where applicable. Gene expression data from purified MM cells were obtained from a publicly available cohort of newly diagnosed MM patients treated with high-dose melphalan and autologous hematopoietic stem cell transplantation (UAMS-TT2 cohort (accession number GSE24080)). We focused on the expression of genes regulated by XBP1, ATF6, and ATF4. We also used Affymetrix data from relapsed MM patients at relapse subsequently treated with bortezomib or dexamethasone monotherapy (accession number GSE9782) from the study by Mulligan et al (Mulligan et al, 2007). We also used a cohort of MM patients at relapse treated by anti-CD38 MoAb (Constantinides et al, 2024; Ovejero et al, 2022). MM cells were purified using anti-CD138 MACS microbeads (Miltenyi Biotec, Bergisch Gladbach, Germany) and their gene expression profile (GEP) obtained using Affymetrix U133 plus 2.0 microarrays as previously described (Ovejero et al, 2022; de Boussac et al, 2020). Gene expression data were normalized with the MAS5 algorithm and analyses processed with GenomicScape (http://www.genomicscape.com) (Kassambara et al, 2015). All statistical analyses were performed with the statistics software R, and R packages developed by the Bioconductor project (https://www.bioconductor.org). The target genes were selected based on their association with these transcription factors as identified through chromatin immunoprecipitation (ChIP) assays and from QIAGEN IPA databases. The XBP1-Pathway signature was computed with 23 XBP1-target genes associated with a prognostic value in MM patients. The XBP1-Pathway signature is defined by the sum of the beta coefficient derived from the Cox model for each prognostic gene weighted by −1 or +1 according to the MMC gene expression above or below the Maxstat R package defined cutpoint as previously described (Alaterre et al, 2022; Gentleman et al, 2004). The statistical significance of differences in overall survival or progression-free survival between patients’ groups was calculated using the log-rank test, and survival curves were plotted using the Kaplan–Meier method.

### Cytotoxicity assays

A total of 20,000 cells per well were seeded in 96-well plates using complete RPMI medium supplemented with 10% fetal calf serum (FCS). Cells were treated in triplicate with varying concentrations of bortezomib (catalog number 5043140001), 4µ8c (catalog number SML0949), Ceapin A7 (catalog number SML2330), and GSK2656157 (catalog number 5046510001). The treatments were administered to evaluate the combined cytotoxic effects under different drug concentration conditions. Cell viability was assessed at 24 and 48 h post-treatment. A supra-vital viability dye, Zombie Yellow™ (1:200 dilution, catalog number 423103), was employed to stain dead cells. Following staining, the cells were washed thoroughly to remove excess dye and then subjected to flow cytometric analysis.

### Statistical analysis

For each figure, the statistical test was selected based on the experimental design and data structure, and is stated in the corresponding figure legend (e.g., Log-rank for Kaplan–Meier survival analyses; Mann–Whitney for two-group comparisons; Kruskal–Wallis for >2 groups/timepoints within a cell line when distributional assumptions could not be ensured; two-way ANOVA for factorial designs such as treatment × time or treatment × dose; Spearman for correlation analyses). When parametric tests were used, assumptions were assessed in Prism (v9) by testing normality (Shapiro–Wilk) and inspecting distribution/QQ plots; homogeneity of variance was assessed using Brown–Forsythe (or equivalent variance checks). If assumptions were not met, we used nonparametric alternatives (Mann–Whitney and Kruskal–Wallis) and/or applied appropriate corrections (e.g., Welch correction for unequal variances, and multiple-comparison correction for post hoc tests when applicable). Survival analyses (Kaplan–Meier/log-rank; Cox models where relevant) do not assume normality. Variation within each group is shown in the graphs (individual data points and/or box plots displaying median and interquartile range, or error bars as specified in the legend). The number of independent experiments (biological replicates) and technical replicates is provided in the corresponding figure legends where applicable. The most appropriate statistical test was chosen according to each set of data, as indicated in Fig. legends with *P* values *P < 0.05; **P < 0.01; ***P < 0.001; ****P < 0.0001. Exact statistical tests and *P* values for all quantitative analyses are provided in Dataset EV1.

## Supplementary information

Appendix

Peer Review File

Dataset EV1

Source data Fig. 2

Source data Fig. 3

Source data Fig. 4

Source data Fig. 5

Source data Fig. 6

Source data Fig. 7

Figure EV1 Source Data

Figure EV2 Source Data

Figure EV3 Source Data

Appendix Figure Source Data

Expanded View Figures

## Data Availability

This study includes no data deposited in external repositories. The source data of this paper are collected in the following database record: biostudies:S-SCDT-10_1038-S44321-026-00469-7.
