# Supplementary material for: Single-nuclei UPR profiling by flow cytometry reveals bortezomib resistance mechanisms in multiple myeloma
Source: EMBO Mol Med. 2026 Jun 29;18(8):3057–85. doi: 10.1038/s44321-026-00469-7 (PMC13470223; doi:10.1038/s44321-026-00469-7)
Supplement: Supplementary file 1 [file 44321_2026_469_MOESM14_ESM.pdf]

Expanded View Figures

Figure EV1. Induction of acute ER stress with Tunicamycin induces different UPR profiles in cell lines.

(**A**) Distinct cancer cell lines were treated with 100 ng/ml of tunicamycin (Tm) for 30 min, 2 h, and 4 h prior to nuclei extraction and further SNUPR profiling of UPR activation by measuring translation (puromycin incorporation) and translocation of ATF6f and XBP1s by flow cytometry. In box plots, the center line indicates the median, box limits indicate the 25th and 75th percentiles, and whiskers indicate the minimum and maximum values. Statistical analysis was performed using the Kruskal–Wallis test for each cell line. *$P < 0.05$, **$P < 0.01$, ***$P < 0.001$ and ****$P < 0.0001$. (**B**) qPCR quantification of relative mRNA levels of UPR-associated transcripts on HeLa and THP-1 cells treated for 30 min, 2 h, and 4 h with thapsigargin (Tg, 400 nM). Statistical analysis was performed using the Kruskal–Wallis test for each cell line. *$P < 0.05$, **$P < 0.01$, ***$P < 0.001$ and ****$P < 0.0001$. Source data are available online for this figure.

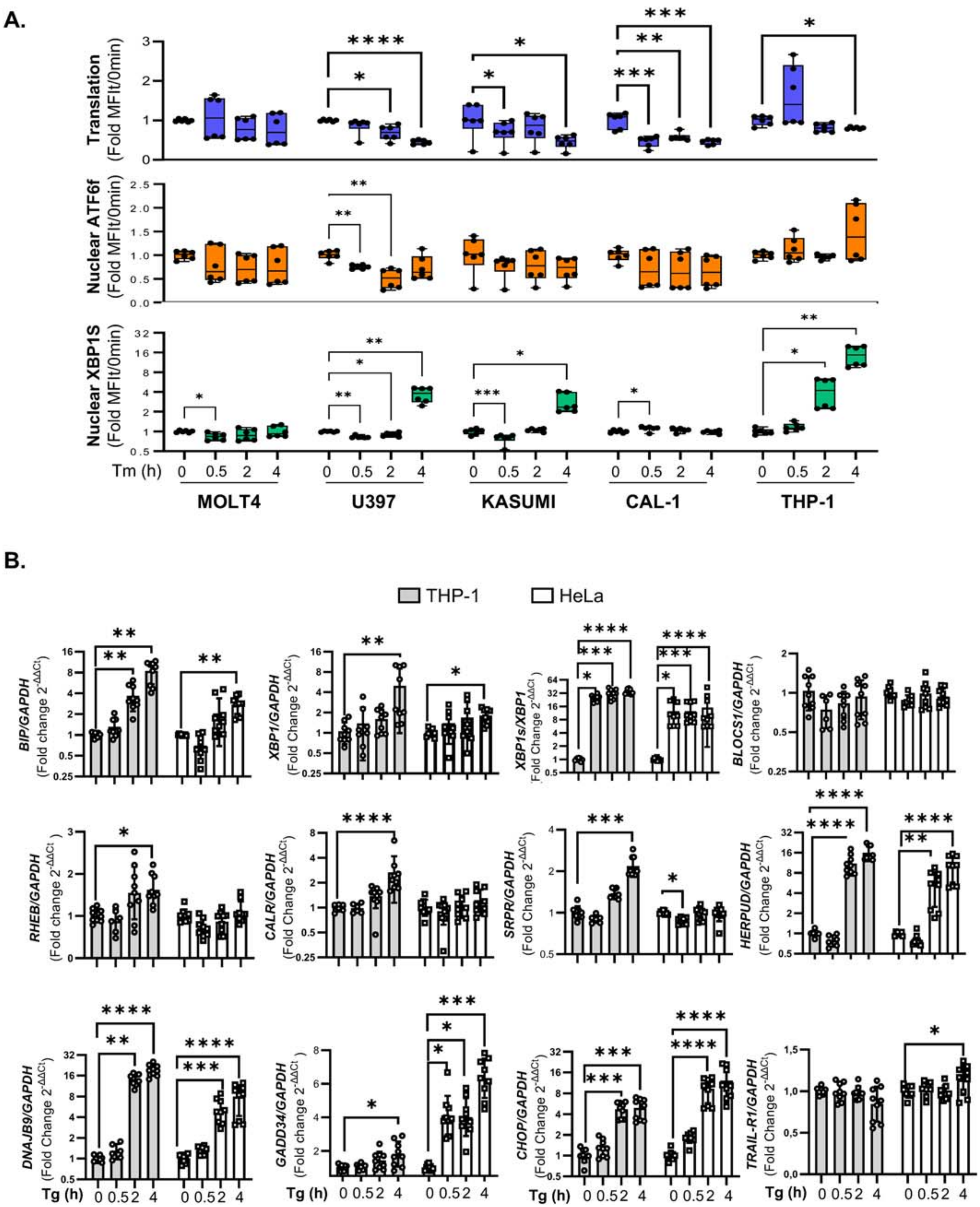
A.
Translation (Fold MFIt/0min)
Nuclear ATF6f (Fold MFIt/0min)
Nuclear XBP1S (Fold MFIt/0min)
Tm (h) 0 0.5 2 4
MOLT4
U397
KASUMI
CAL-1
THP-1
B.
THP-1
HeLa
BIP/GAPDH
XBP1/GAPDH
XBP1s/XBP1
BLOCS1/GAPDH
RHEB/GAPDH
CALR/GAPDH
SRPR/GAPDH
HERPUD/GAPDH
DNAJB9/GAPDH
GADD34/GAPDH
CHOP/GAPDH
TRAIL-R1/GAPDH
Tg (h) 0 0.5 2 4

 © The Author(s)

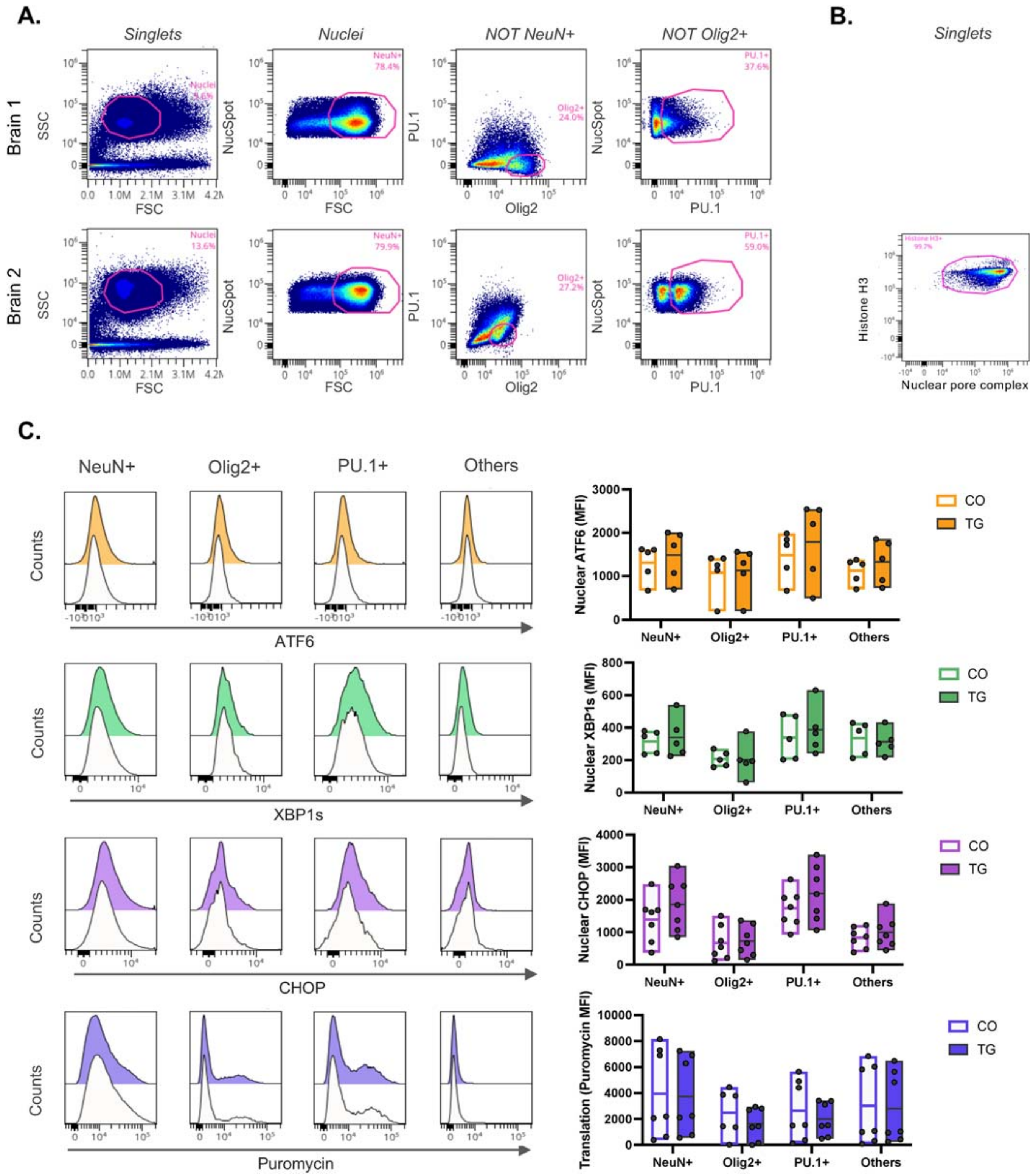
A.
Singlets
Nuclei
NOT NeuN+
NOT Olig2+
Brain 1
Brain 2
SSC
FSC
NucSpot
PU.1
Olig2
Nuclei
NeuN+
Olig2+
PU.1+
B.
Singlets
Histone H3
Nuclear pore complex
C.
NeuN+
Olig2+
PU.1+
Others
Counts
ATF6
XBP1s
CHOP
Puromycin
Nuclear ATF6 (MFI)
Nuclear XBP1s (MFI)
Nuclear CHOP (MFI)
Translation (Puromycin MFI)
CO
TG

Figure EV2. Cell-type-specific nuclear marker staining enables SNUPR profiling of mouse brain cell subsets.

Nuclei isolated from mouse brain slices were stained intracellularly for neural cells–specific nuclear markers and analyzed by flow cytometry. Neuronal, oligodendrocyte, and microglial/MDMC nuclei were identified based on NeuN, Olig2, and PU.1 expression, respectively. (**A**) Representative gating strategy for singlet nuclei selection using FSC/SSC and NucSpot staining, followed by identification of $NeuN^+$, $Olig2^+$, and $PU.1^+$ nuclear populations. The data shown are representative of 2 out of 5 independent brain specimens. (**B**) Validation of nuclei identification by co-staining with independent nuclear markers, including total histone H3 and nuclear pore complex proteins. (**C**) SNUPR profiling of cell-type-specific stress responses in brain tissue. Brain slices were treated with thapsigargin (Tg; 400 nM, 2 h) followed by a 40 min puromycin pulse prior to nuclei isolation. Nuclear translocation of ATF6f, XBP1s, and CHOP, as well as translational activity assessed by puromycin incorporation, were quantified by flow cytometry. Box plots show median fluorescence intensity (MFI) values for neuronal ($NeuN^+$), oligodendrocyte ($Olig2^+$), microglial/MDMC ($PU.1^+$), and Other nuclear populations from three independent experiments with technical triplicates (XBP1s, $n = 2$). Source data are available online for this figure.

 © The Author(s)

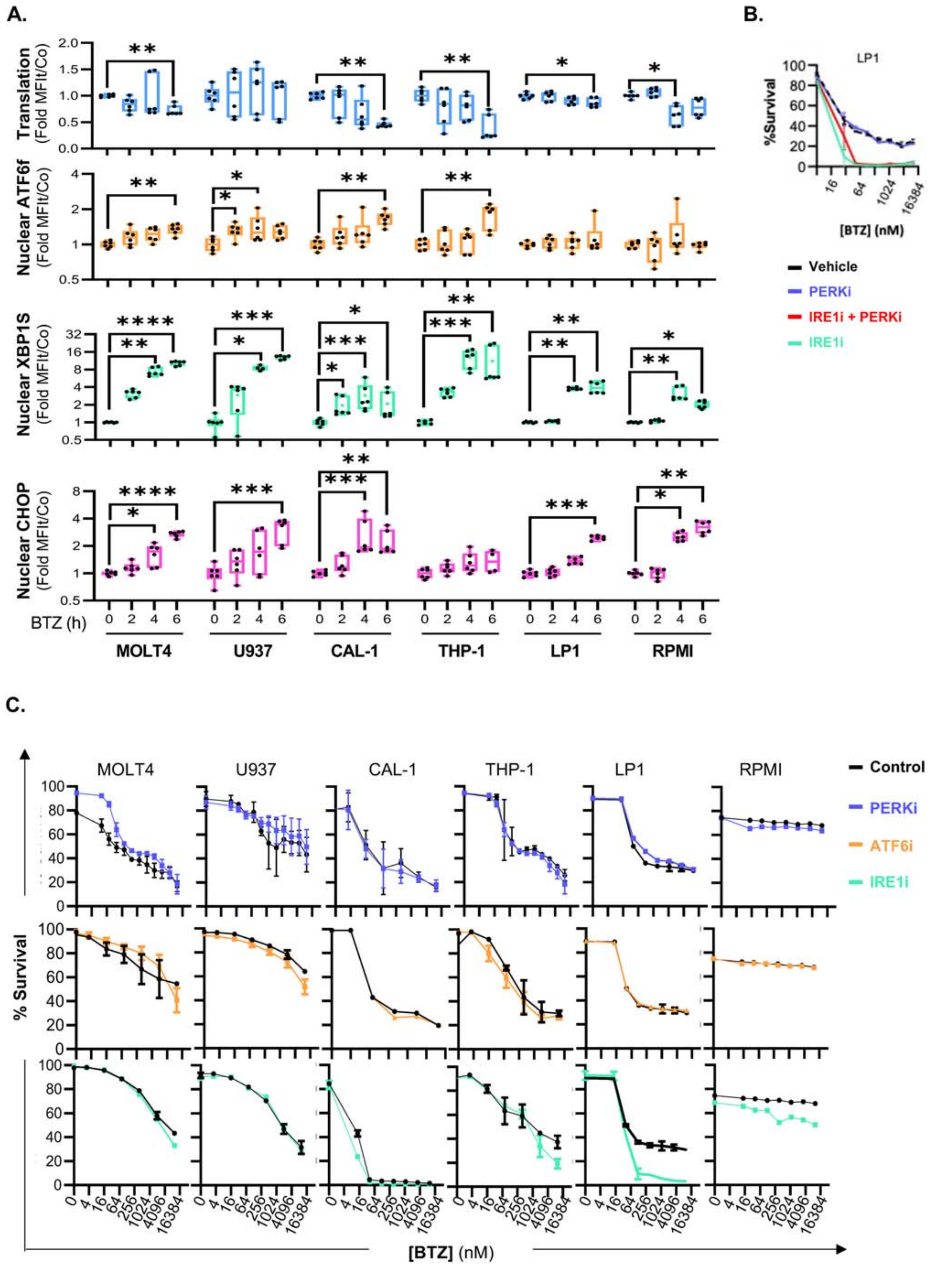
A.
B.
Translation (Fold MFIt/Co)
Nuclear ATF6f (Fold MFIt/Co)
Nuclear XBP1S (Fold MFIt/Co)
Nuclear CHOP (Fold MFIt/Co)
BTZ (h)
MOLT4
U937
CAL-1
THP-1
LP1
RPMI
%Survival
[BTZ] (nM)
Vehicle
PERKi
IRE1i + PERKi
IRE1i
C.
% Survival
Control
PERKi
ATF6i
IRE1i
[BTZ] (nM)

◀ **Figure EV3. Bortezomib induces UPR activation in leukemic and myeloma cells.**

Leukemia cell lines and two multiple myeloma (MM) cell lines, LP1 and RPMI, were treated with the proteasome inhibitor bortezomib (BTZ, 100 nM) for 2, 4, and 6 h prior to nuclei isolation and SNUPR profiling. (**A**) SNUPR profiles of translation levels and ATF6f and XBP1s nuclear translocation of cancer cells treated with BTZ for 2 h, 4 h or 6 h. in box plots, the center line indicates the median, box limits indicate the 25th and 75th percentiles, and whiskers indicate the minimum and maximum values. Statistical analysis was performed using the Kruskal–Wallis test for each cell line. **P* < 0.05, ***P* < 0.01, and ****P* < 0.001. (**B**) Cell survival analysis of LP1 cells treated 6 h with BTZ in the presence or absence of IRE1α inhibitor 4u8c (10 μM). PERK inhibitor GSK2656157 (100 nM) or a combination of both was measured by flow cytometry. (**C**) Survival analysis of leukemia and multiple myeloma cells treated with a gradient concentration of BTZ in combination with a PERK inhibitor (GSK2656157, 100 nM, blue). ATF6 inhibitor (Ceapin A7, 6 μM orange) or IRE1α inhibitor (4u8, green) for 24 h. All analyses were performed by flow cytometry measurements of cell viability dye signals. Source data are available online for this figure.

 © The Author(s)

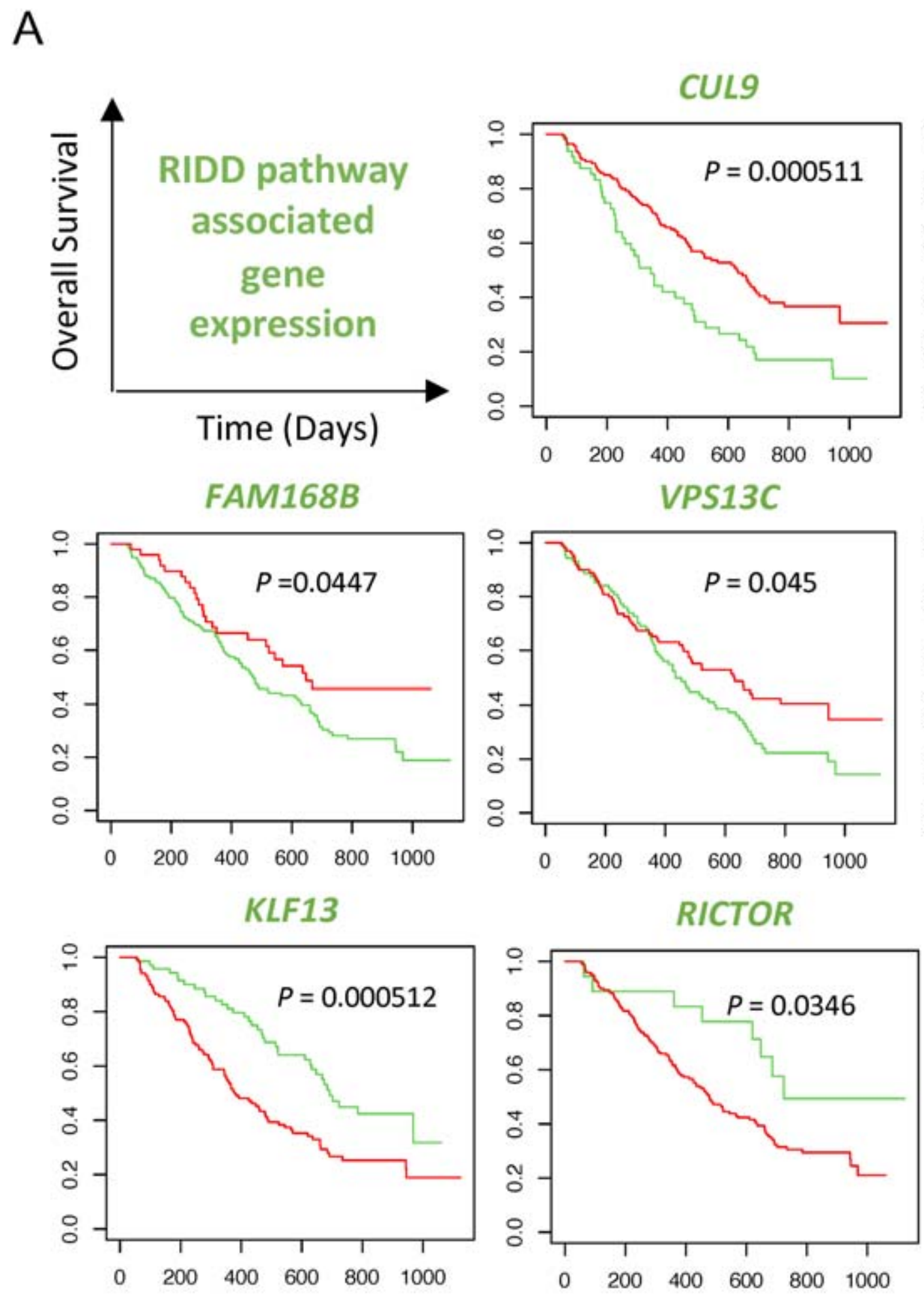

Figure EV4. RIDD gene targets expression associated with a favorable outcome in multiple myeloma patients treated with bortezomib.

(**A**) Multiple myeloma (MM) patients from the Mulligan cohort treated with bortezomib (BTZ) monotherapy were stratified according to the expression level of a curated set of IRE1α-dependent RIDD target genes. Kaplan–Meier survival curves for individual RIDD-associated genes showing significant prognostic value in the Mulligan BTZ monotherapy cohort. For each gene, patients were stratified based on low expression levels (green curves) and high (red curves).
