# Supplementary material for: Single-nuclei UPR profiling by flow cytometry reveals bortezomib resistance mechanisms in multiple myeloma
Source: EMBO Mol Med. 2026 Jun 29;18(8):3057–85. doi: 10.1038/s44321-026-00469-7 (PMC13470223; doi:10.1038/s44321-026-00469-7)
Supplement: Supplementary file 2 [file 44321_2026_469_MOESM1_ESM.pdf]

Table of Contents

APPENDIX FIGURE S1. BRANCH-SPECIFIC UPR SIGNATURES CORRELATE WITH SURVIVAL PROGNOSIS OF PATIENTS WITH ACUTE MYELOID LEUKEMIA. 2

APPENDIX FIGURE S2. RIDD TARGETS EXPRESSION CORRELATE WITH SURVIVAL PROGNOSIS OF AML PATIENTS. 3

APPENDIX FIGURE S3. QUALITY CONTROL OF NUCLEAR EXTRACTION FOR SNUPR PROFILING. 5

APPENDIX FIGURE S4. CORRELATION OF TRANSLATION INHIBITION WITH REDUCED XBP1S EXPRESSION. 6

APPENDIX FIG. S5. IRE1A INHIBITION AFFECTS ER CHAPERON EXPRESSION ON MM CELLS. 7

APPENDIX TABLE S1. RIDD TARGETS WHOSE LEVELS SHOWED STATISTICALLY SIGNIFICANT ASSOCIATION WITH PROGNOSIS IN PATIENTS TREATED WITH BORTEZOMIB....................................8

Appendix Figure S1. Branch-specific UPR signatures correlate with survival prognosis of patients with Acute Myeloid Leukemia.

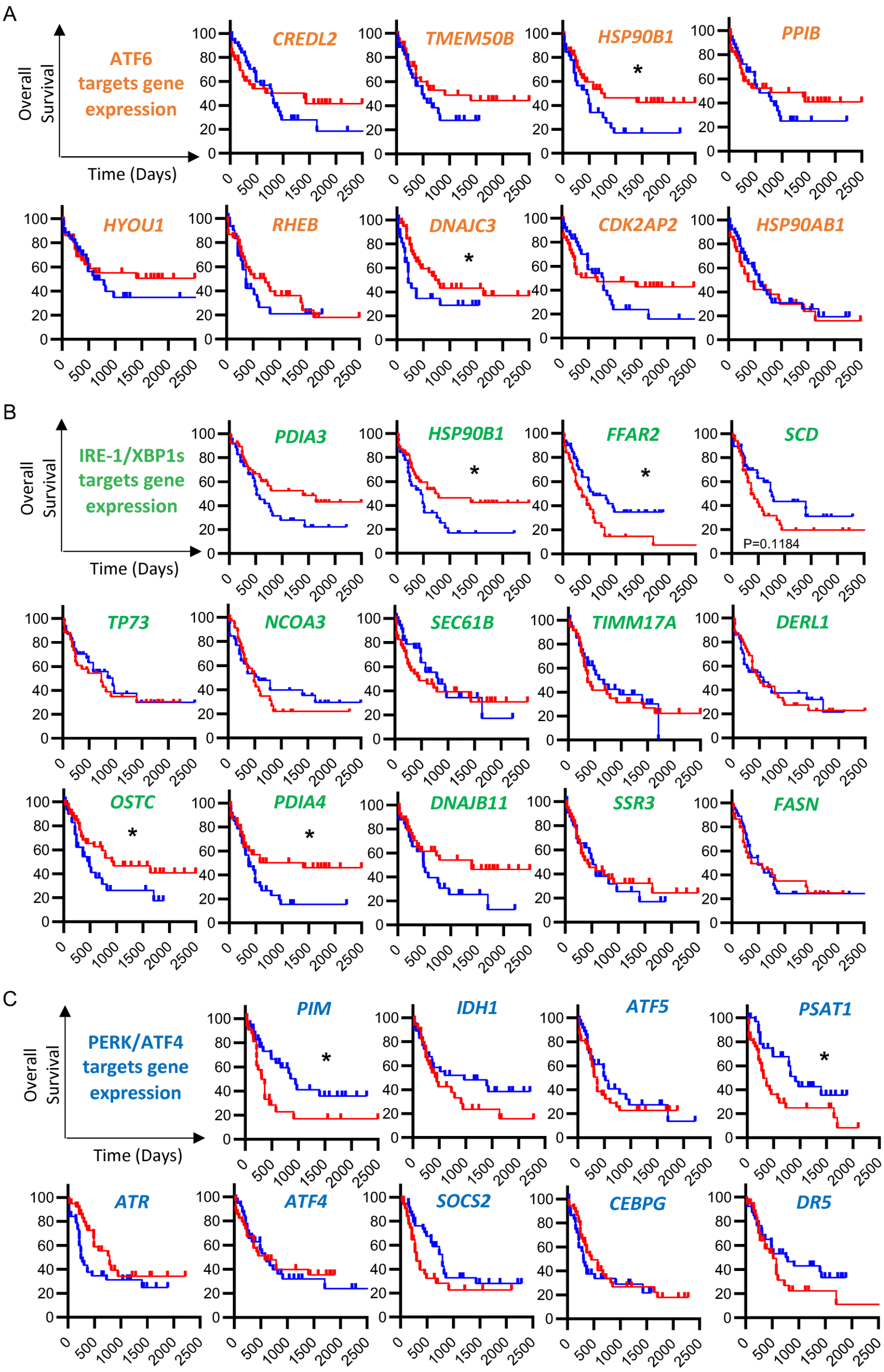

Appendix Figure S1. Branch-specific UPR signatures correlate with survival prognosis of patients with Acute Myeloid Leukemia. (A-C) Kaplan Meier survival curves for genes under the control of ATF6, XBP1s or PERK generated for AML. Kaplan Meier curves were done based on dichotomized gene expression, specifically for values below quartile 1 (blue) and above quartile 3 (red). Genes indicated are described to be under the control of ATF6 (A); IRE-1/XBP1s (B) and PERK/ATF4 (C). Statistical analysis was performed using Log-rank test. *$P < 0.05$.

Appendix Figure S2. RIDD targets expression correlate with survival prognosis of AML patients.

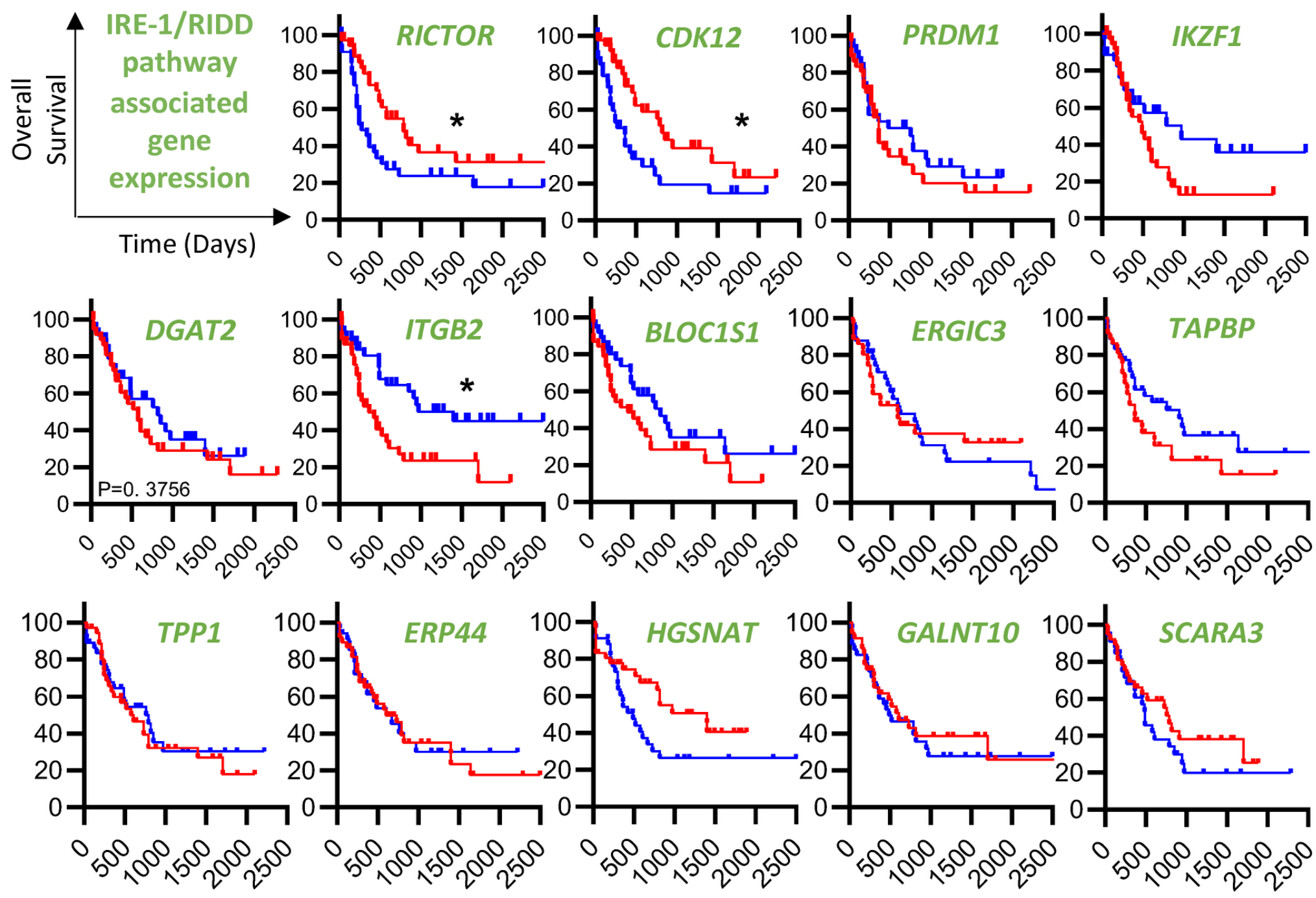

Appendix Figure S2. RIDD targets expression correlate with survival prognosis of AML patients. Kaplan Meier survival curves for mRNAs targeted by RIDD were generated for AML. Kaplan Meier curves were done based on dichotomized gene expression, specifically for values above quartile 1 (blue) and quartile 3 (red). Statistical analysis was performed using Log-rank test. *$P < 0.05$.

Appendix Figure S3. Quality control of nuclear extraction for SNUPR profiling

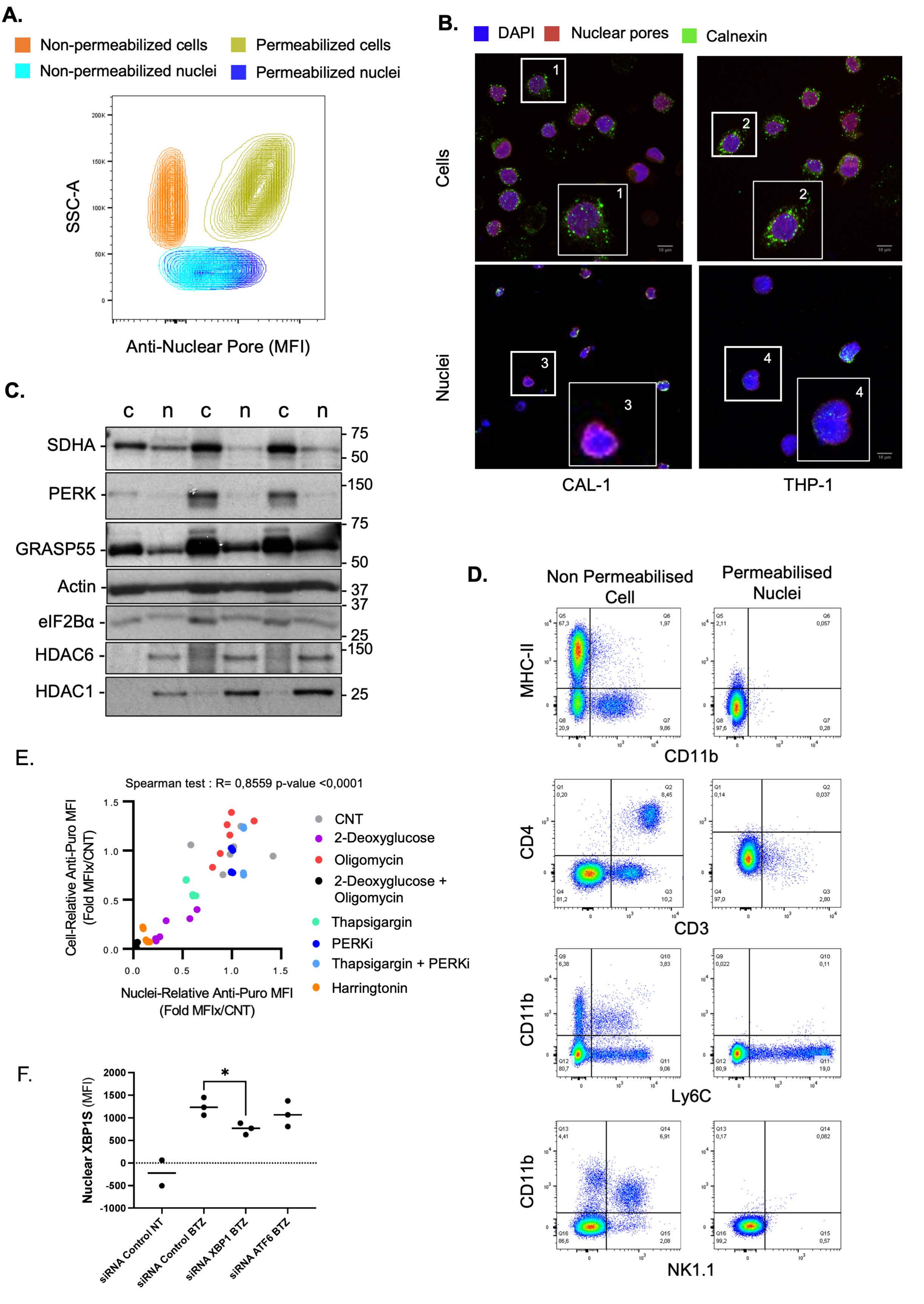

Appendix Figure S3. Quality control of nuclear extraction for SNUPR profiling.

(A) Nuclei or whole CAL-1 cells were permeabilized or not and stained with nucleopore antibody. (**B**) Staining of nuclear pores and calnexin was analysed by microscopy in CAL-1 and THP-1 whole cells and nuclei. (**C**) CAL-1 cells (c) or nuclei (n) suspensions were lysed and analysed by immunoblot for markers of mitochondria (SDHA), endoplasmic reticulum (PERK), Golgi (GRASP55), cytoplasm (eIF2Ba, Actin) and nucleus (HDAC6 and HDAC1). The three sets of c/n samples correspond to 3 independent biological replicates. (**D**) Whole cells and nuclei extract from mouse blood stained with indicated surface markers. (**E**) Correlation of puromycin MFI (normalized to control) of cells versus isolated nuclei in response to different pharmacological compounds. (F) anti-XBP1 FACS staining in LP1 MM cells treated with BTZ or not (NT), and lipofected for 72 hours with siRNA siCo, siXBP1 or siATF6. Spearman test: R= 0,8559 p-value <0,0001. CNT: Control.

Appendix Figure S4. Correlation of translation inhibition with reduced XBP1s expression.

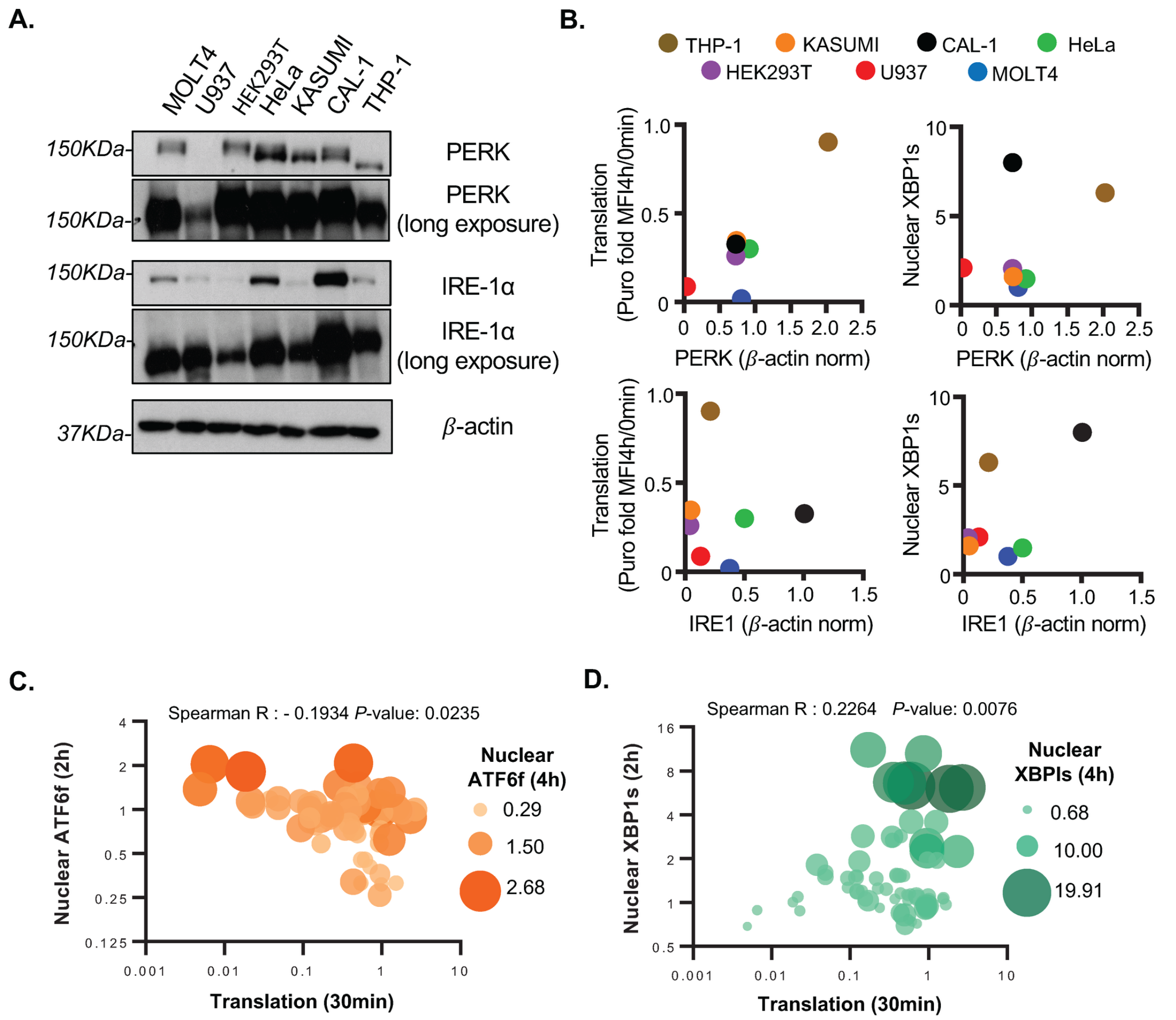

Appendix Figure S4. Correlation of translation inhibition with reduced XBP1s expression. (A) Quantification of IRE-1a and PERK protein levels on different cell lines was assessed by immunoblot. **(B)** Correlation analyses between expression levels of IRE1α and PERK with translation levels or nuclear translocation of XBP1s for each cell line was assessed. Further correlation analyses between early translation levels (30min) and nuclear expression of **(C)** ATF6 and **(D)** XBP1s after 2h of ER stress induction was evaluated using the Spearman test. Spearman R-score and p-values are depicted in the lower panel.

Appendix Figure S5. IRE-1 inhibition affects ER chaperone expression on MM cells.

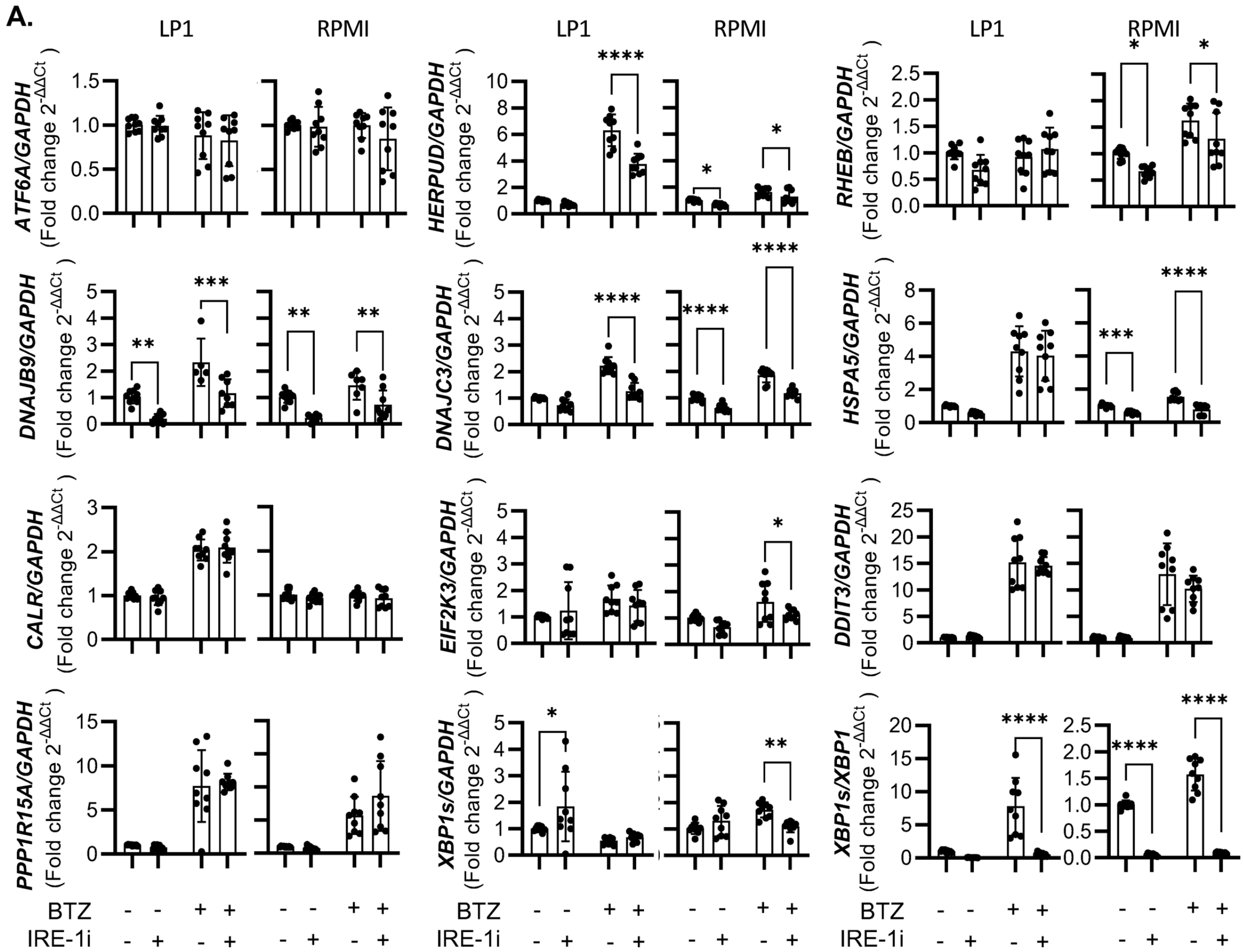

Appendix Fig. S5. IRE1α inhibition affects ER chaperon expression on MM cells. **(A)** RT-qPCR analysis of ER chaperones and other UPR-associated transcript mRNA levels of LP1 and RPMI MM cell lines treated with BTZ (100nM) for 4h in the presence or absence of IRE1α inhibitor 4µ8c (10µM). Statistical analysis was performed using 2way Annova test. *$P < 0.05$, **$P < 0.01$, and ***$P < 0.001$.

Appendix Table S1. RIDD targets whose levels showed statistically significant association with prognosis in patients treated with bortezomib.

Gene Name	Entrez Gene ID	Evidence Status	Primary Evidence	References
KLF13	51621	Validated RIDD Target	Validated by RNA-seq + in vitro cleavage assay in myeloma; Confirmed by Western blot upon ER stress	Quwaider et al. 2022, PMID 35361260
NOTCH1	4851	Validated RIDD Target	Confirmed protein degradation upon ER stress (4h-16h time course); IRE1-dependent cleavage validated	Quwaider et al. 2022, PMID 35361260
RICTOR	253260	Validated RIDD Target	Identified in comprehensive RNA-seq; Confirmed stem-loop structure for IRE1 cleavage	Quwaider et al. 2022, PMID 35361260
FAM168b	100131165	Validated RIDD Target	Identified in RNA-seq with XBP1-like consensus sequence in stem-loop; In vitro cleavage confirmed	Quwaider et al. 2022, PMID 35361260
VPS13C	100464581	Identified RIDD Target	Predicted based on transcriptomic analysis in ER stress context; Lipid transfer protein	Lipid homeostasis studies; ER stress response profiles
BIRC6	57448	Identified RIDD Target	Associated with IRE1-dependent decay and apoptosis regulation	Unfolded protein response literature; BIRC family studies
CUL9	23530	Identified RIDD Target	E3 ubiquitin ligase component identified in ER stress transcriptomics	Broad ER stress response analyses
UBR3	8354	Identified RIDD Target	N-end rule pathway component implicated in RIDD-dependent protein degradation	N-end rule pathway and RIDD mechanism integration studies
CUL5	8065	Identified RIDD Target	E3 ubiquitin ligase complex component involved in ER stress adaptation	ER stress response and proteasomal degradation studies
