# Supplementary material for: Single-nuclei UPR profiling by flow cytometry reveals bortezomib resistance mechanisms in multiple myeloma
Source: EMBO Mol Med. 2026 Jun 29;18(8):3057–85. doi: 10.1038/s44321-026-00469-7 (PMC13470223; doi:10.1038/s44321-026-00469-7)
Supplement: Supplementary file 3 [file 44321_2026_469_MOESM2_ESM.pdf]

EMBO Molecular Medicine

Single-nuclei UPR profiling by FACS reveals bortezomib resistance mechanisms in multiple myeloma

Julien Gigan, Paulina Garcia-Gonzalez, Angelo Pilotti, Lou Galliot, Alexandre Reynaud, Yoan Ghaffar, Felipe Flores-Santibanez, Sara Ghafoori, Eve Seiller, Rosario Lavignolle-Heguy, daniela Barros, Caroline Bret, Sharon Fichaux, Alexis Combes, Alessandro BANI, Rejane RUA, Evelina Gatti, Béatrice Nal Rogier, Stéphane Rocchi, Jérôme Moreaux, Philippe Pierre, and Rafael Argüello

Corresponding author(s): Rafael Argüello (Rafael.arguello@cnrs.fr) , Philippe Pierre (pierre@ciml.univ-mrs.fr)

Review Timeline:

Submission Date:	22nd Apr 25
Editorial Decision:	17th Jun 25
Revision Received:	16th Apr 26
Editorial Decision:	12th May 26
Revision Received:	28th May 26
Accepted:	1st Jun 26

Editor: Jingyi Hou

Transaction Report:

(Note: With the exception of the correction of typographical or spelling errors that could be a source of ambiguity, letters and reports are not edited. Depending on transfer agreements, referee reports obtained elsewhere may or may not be included in this compilation. Referee reports are anonymous unless the Referee chooses to sign their reports.)

17th Jun 2025

Dear Dr. Argüello,

Thank you for the submission of your manuscript to EMBO Molecular Medicine. First of all, I would like to apologise for the slow process. We have now heard back from two of the three reviewers who agreed to evaluate your manuscript. Unfortunately, after a series of reminders, we did not manage to obtain a report from Reviewer #2. In the interest of time, and since the recommendations of the other two reviewers are quite similar, I prefer to make a decision now rather than further delaying the process. If we receive the comments from Reviewer #2, we will send them to you, and you can address the issues raised by Reviewer #2 together with those raised by the other two reviewers. You will see from the comments below that Reviewers #1 and #3 find the manuscript to be of potential interest. However, they raise substantial concerns on your work, which should be convincingly addressed in a major revision of the present manuscript.

I think the reviewers' recommendations are clear and do not require reiteration. Notably, Reviewer #3 suggested that the study would be strengthened by extending the in vitro application in cultured cell lines to the tissue or tumor microenvironment. We think that addressing this point is essential to demonstrate the advantages and utility of the SNUPR method and to enhance the overall clinical relevance of the work, which is an important consideration for publication in EMBO Molecular Medicine. All other issues raised by the reviewers need to be satisfactorily addressed as well.

Overall, it is clear that publication of the manuscript cannot be considered at this stage. We also note that addressing the reviewers concerns in full will be necessary for further considering the manuscript in our journal and this appears to require a lot of additional work and experimentation. Given the scope of the revisions needed, we are unsure whether you will be able or willing to address those and return a revised manuscript within the six months deadline.

That said, given the potential interest of the study, we would be willing to consider a revised manuscript with the understanding that the referee concerns must be fully addressed, and that acceptance of the manuscript would entail a second round of review.

As you may already know, our editorial policy allows in principle a single round of major revision and that, therefore, acceptance or rejection of the manuscript will depend on the completeness of your responses included in the next, final version of the manuscript. For this reason, and to avoid potential frustrations, we would strongly advise against returning an incomplete revision and would also understand your decision if you choose to rather seek rapid publication elsewhere at this stage.

Should you decide to embark in such a revision, revised manuscripts should be submitted within six months of a request for revision; they will otherwise be treated as new submissions, except under exceptional circumstances in which a short extension is obtained from the editor.

I look forward to seeing a revised form of your manuscript soon.

Should you find that the requested revisions are not feasible within the constraints outlined here and choose, therefore, to submit your paper elsewhere, we would welcome a message to this effect.

Sincerely,
Jingyi

Jingyi Hou
Senior Editor
EMBO Molecular Medicine

*****

When submitting your revised manuscript, please carefully review the instructions that follow below. We perform an initial quality control of all revised manuscripts before re-review; failure to include requested items will delay the evaluation of your revision.

We require:

1) A .docx formatted version of the manuscript text (including legends for main figures, EV figures and tables). Please make sure that the changes are highlighted to be clearly visible.

2) Individual production quality figure files as .eps, .tif, .jpg (one file per figure). For guidance, download the 'Figure Guide PDF' (https://www.embopress.org/page/journal/17574684/authorguide#figureformat).

3) A .docx formatted letter INCLUDING the reviewers' reports and your detailed point-by-point responses to their comments. As part of the EMBO Press transparent editorial process, the point-by-point response is part of the Review Process File (RPF), which will be published alongside your paper.

4) A complete author checklist, which you can download from our author guidelines (https://www.embopress.org/page/journal/17574684/authorguide#submissionofrevisions). Please insert information in the checklist that is also reflected in the manuscript. The completed author checklist will also be part of the RPF.

5) Please note that all corresponding authors are required to supply an ORCID ID for their name upon submission of a revised manuscript.

6) It is mandatory to include a 'Data Availability' section after the Materials and Methods. Before submitting your revision, primary datasets produced in this study need to be deposited in an appropriate public database, and the accession numbers and database listed under 'Data Availability'. Please remember to provide a reviewer password if the datasets are not yet public (see https://www.embopress.org/page/journal/17574684/authorguide#dataavailability).

In case you have no data that requires deposition in a public database, please state so in this section. Note that the Data Availability Section is restricted to new primary data that are part of this study.

7) For data quantification: please specify the name of the statistical test used to generate error bars and P values, the number (n) of independent experiments (specify technical or biological replicates) underlying each data point and the test used to calculate p-values in each figure legend. The figure legends should contain a basic description of n, P and the test applied. Graphs must include a description of the bars and the error bars (s.d., s.e.m.).

8) We would also encourage you to include the source data for figure panels that show essential data. Numerical data should be provided as individual .xls or .csv files (including a tab describing the data). For blots or microscopy, uncropped images should be submitted (using a zip archive if multiple images need to be supplied for one panel). Additional information on source data and instruction on how to label the files are available at

.

9) Our journal encourages inclusion of *data citations in the reference list* to directly cite datasets that were re-used and obtained from public databases. Data citations in the article text are distinct from normal bibliographical citations and should directly link to the database records from which the data can be accessed. In the main text, data citations are formatted as follows: "Data ref: Smith et al, 2001" or "Data ref: NCBI Sequence Read Archive PRJNA342805, 2017". In the Reference list, data citations must be labeled with "[DATASET]". A data reference must provide the database name, accession number/identifiers and a resolvable link to the landing page from which the data can be accessed at the end of the reference. Further instructions are available at .

10) We replaced Supplementary Information with Expanded View (EV) Figures and Tables that are collapsible/expandable online. A maximum of 5 EV Figures can be typeset. EV Figures should be cited as 'Figure EV1, Figure EV2" etc... in the text and their respective legends should be included in the main text after the legends of regular figures.

- For the figures that you do NOT wish to display as Expanded View figures, they should be bundled together with their legends in a single PDF file called *Appendix*, which should start with a short Table of Content. Appendix figures should be referred to in the main text as: "Appendix Figure S1, Appendix Figure S2" etc.

- Additional Tables/Datasets should be labeled and referred to as Table EV1, Dataset EV1, etc. Legends have to be provided in a separate tab in case of .xls files. Alternatively, the legend can be supplied as a separate text file (README) and zipped together with the Table/Dataset file.

See detailed instructions here:

.

11) The paper explained: EMBO Molecular Medicine articles are accompanied by a summary of the articles to emphasize the major findings in the paper and their medical implications for the non-specialist reader. Please provide a draft summary of your article highlighting

- the medical issue you are addressing,

- the results obtained and

- their clinical impact.

This may be edited to ensure that readers understand the significance and context of the research. Please refer to any of our published articles for an example.

12) For more information: There is space at the end of each article to list relevant web links for further consultation by our readers. Could you identify some relevant ones and provide such information as well? Some examples are patient associations, relevant databases, OMIM/proteins/genes links, author's websites, etc...

13) Author contributions: the contribution of every author must be detailed in a separate section (before the acknowledgments).

14) A Conflict of Interest statement should be provided in the main text.

15) Every published paper now includes a 'Synopsis' to further enhance discoverability. Synopses are displayed on the journal webpage and are freely accessible to all readers. They include a short stand first (maximum of 300 characters, including space) as well as 2-5 one-sentences bullet points that summarizes the paper. Please write the bullet points to summarize the key NEW findings. They should be designed to be complementary to the abstract - i.e. not repeat the same text. We encourage inclusion of key acronyms and quantitative information (maximum of 30 words / bullet point). Please use the passive voice. Please attach these in a separate file or send them by email, we will incorporate them accordingly.

Please also suggest a visual abstract to illustrate your article as a PNG file 550 px wide x 300-800 px high.

EMBO Molecular Medicine has a "scooping protection" policy, whereby similar findings that are published by others during review or revision are not a criterion for rejection. Should you decide to submit a revised version, I do ask that you get in touch after three months if you have not completed it, to update us on the status.

***** Reviewer's comments *****

Referee #1 (Comments on Novelty/Model System for Author):

n/a

Referee #1 (Remarks for Author):

In this manuscript, Gigan and colleagues propose single nuclei RNAseq analyses to evaluate the role of the Unfolded Protein Response in the mechanisms invovled in bortezomib resistance.

General comments:
From the title it would be expected that single nuclei RNAseq would be shown but rather it is FACS analyses, and in the end, RNAseq analyses are more bulk-based. One can note a general lack of resolution for all the figures.

What's new : The importance of translation in activating the UPR pathways. SNUPR could represent a useful tool for the community to evaluate the activation of UPR branches at the single cell level.

The role of IRE1/XBP1s in MM sensitivity to proteasome inhibitors has already been shown previously (PMID: 32039016).

Specific comments :

Higlights at the beginning of the article read like overstatements

Fig1B-D - P values are missing - Why did the authors choose these two cohorts specifically?

Fig2B - Tg is used for the analysis of the ATF6 and PERK branches, why is Tm used for the IRE1 branch (it is alos activated upon Tg treatment)? How do the authors explain the import of puro-peptides into the nucleus? Did the authors verify the activation of XBP1 when ATF6 and PERK are inhibited, and the same for ATF6f and translation when the other two inhibitors are inhibited? Have the authors checked whether the concentration of puromycin used induces ER stress? Cleaved and uncleaved ATF6 forms should be shown on the same Western blot.

Fig3B - XBP1s is regulated from 2h in Fig. 3A but this does not appear to be the case in Fig3A (in the SNUPR part). Can the authors explain this difference? Furthermore, the Z-score legend is not annotated with numbers.

Fig5A - 4u8c was demonstrated to display strong off-target effects, validation with several other IRE1 inhibitors, and genetical modulation of IRE1/XBP1 would strengthen the conclusions for the main findings. Since a cross talk between PERK and IRE1 signaling has recently been identified (PMID: 38637497), PERKi and IRE1i should be also tested to assess the relationship to the activation of other UPR branches, genetical means of targeting the UPR should also be used throughout the study.

Fig5B (and all cell survival analyses in the manuscript) - the X-axis should start at 0 and could also be log-transformed. P values are also missing.

Fig5C/D : The authors found with their technique that the increase in ATF6f is not consistent with ATF6 transcriptional activity, which would in fact highlight that ATF6f is not a good surrogate for ATF6 activity. Could the authors provide a correlation analysis between ATF6f detection using their technique and qPCR on ATF6?
Fig6 : Interpretation of Figure 6 does not match the data. 6A: HA15 does not triggers nuclear translocation of XBP1s in both cell lines. 6C: translation levels are not "slightly reduced".

Is there any contribution of RIDD to BTZ and/or HA15 regulation of survival? Same question for the contribution of XBP1s.

Fig7 : Further explanation on the choice of the genes for XBP1s signature, and comparison to other published XBP1 signature should be provided. What is the contribution of RIDD to this phenomenon.

Discussion points regarding already known crosstalks between UPR branches should be added.

Referee #3 (Comments on Novelty/Model System for Author):

1. The SNUPR technology, when exclusively used on in vitro cultured cell lines, doesn't seem to have advantage over simple Western blotting analysis based on nuclear-cytoplasmic fractionation. The real power of SNUPR would be to reveal the heterogeneity in UPR activation in cells growing in the tissue/tumor microenvironment, especially in solid tumors where microenvironmental factors like hypoxia have profound effects on the UPR, as well as differential activation/deactivation of the UPR in the different cell types (tumor vs. stroma vs. immune cells, etc.), especially when the sample quantity is limited. However, this study lacks any attempt to examine the application of SNUPR in these settings.
2. Profiling in vitro cultured cell lines with SNUPR revealed a great level of variation and inconsistency across different cell types, treatments, as well as the dynamics of the individual UPR branches, for which the current study struggled to reconcile in many cases. Although some major observations were obtained (e.g. translational arrest determines IRE1-XBP1 activity), most of the suboptimal induction and the possible reasons behind that were not explained or investigated.

Referee #3 (Remarks for Author):

Page 2:
• Typo: "which could not be have been predicted"
• Please be consistent with "IRE-1/XBP1" or "IRE-1/XBP1s". And IRE-1 is not the standard gene/protein symbol. It should be "IRE1alpha", as IRE1beta is not relevant in blood cancer cells.
• "Misfolded proteins": should be "misfolded and unfolded proteins".
• "The UPR comprises distinct signalling branches, each activated by the dissociation in the ER lumen of the HSPA5 chaperone (BiP) from three ER-resident sensors." This is one proposed mechanism and other mechanisms, e.g. direct interaction between the sensors with unfolded/misfolded proteins, cannot be ruled out.
• "Chemotherapies": proteasome inhibitor should be considered as a type of targeted therapy.
Page 3:
• "Overexpressing" should be "higher expression".
• For RIDD activity of IRE1, references 16-21 and Fig. S2 do not include more recently identified RIDD targets, e.g. PPP2R1A and RUVBL1 (PMID: 32409639), BCAM and TGOLN2 (PMID: 34911951), GCLC and SLC7A11 (PMID: 38750057).
• It will be interesting to evaluate the concordance and discordance between XBP1s target gene expression and IRE1 RIDD target gene levels in both types of cancer. It is an equally important and interesting question how the RIDD and XBP1s-splicing activities of IRE1 are coordinated in cancer cells.
Page 4:
• "Overexpressed" mainly referes to "beyond normal range". Above quantile 3 is still within the expression level distribution and should be described as "high expression".
• Fig. S3B: The resolution of the IF images is too low for appropriate evaluation of the staining.
• Fig. S3C: What are the 3 sets of c-n samples from the left to the right side of the blot? Are these replicates? Although the

legend states "Relative quantification of each protein is indicated on the right side of the panel", there are only molecular weights shown on the right side of the panel. Which protein markers correspond to "plasma membrane, mitochondrial, lysosomal, and endoplasmic reticulum contaminants" is not described.

• Antibodies used for flow cytometry (Table 1) analysis of the UPR transcription factors need to be verified with Western blotting, as some of these antibodies are not widely used in the field.

Page 5:

• As the antibodies used for flow cytometry analysis of the UPR transcription factors were not verified using a second approach (e.g. Western blotting), it is not clear whether the lack of induction during Tg treatment (e.g. ATF6f in U937/Hela/HEK293T/KASUMI and XBP1s in MOLT4 cells) was due to expression levels of ATF6f/XBP1s in these cell lines lower than the sensitivity threshold of the antibodies.

Page 6:

• Fig. 4: Although the legend mentioned PBMCs, no data on PBMCs are presented in Fig. 4, only THP-1 in A and HeLa in B.

• Fig. S6A: How to explain that ISRIB induced XBP1s mRNA levels, either with or without Tg treatment, either normalized to total XBP1 (2nd panel on the top) or GAPDH (3rd panel on the top)? However, this dramatic induction of XBP1s mRNA didn't lead to upregulation of the XBP1 target genes (SRPRA, HERPUD1 and RHEB, bottom panels). No discussion was provided.

Page 7:

• Fig. S7A-B: It doesn't seem that GATA3 or BCL6 levels are enriched in T cells, though the authors claim they're intracellular T cell lineage-specific transcription factors.

Page 8:

• "Moreover, in LP1 MM cells, a BTZ resistant subpopulation persisted even at higher doses of BTZ (Figure 5A, supplemental 8B and 8C, bottom)." It is not sure what this "subpopulation" refers to in these figure panels, as they're all whole-population analyses.

Point-by-Point Response to Referee #1

We thank Referee #1 for the thorough review of our manuscript and for the constructive feedback provided. We appreciate the recognition of the novelty of our SNUPR approach and its potential utility for assessing activation of the unfolded protein response (UPR) pathways at the single-cell level. We also value the insightful comments that helped us to clarify aspects of our methodology, refine the presentation of our results, and strengthen the discussion in light of prior studies. In the following point-by-point response, we address each comment raised by the reviewer, highlighting the changes made to the manuscript and indicating, sections that correspond to newly added experiments or analyses.

Comment 1:

From the title it would be expected that single nuclei RNAseq would be shown but rather it is FACS analyses, and in the end, RNAseq analyses are more bulk-based. One can note a general lack of resolution for all the figures. The role of IRE1/XBP1s in MM sensitivity to proteasome inhibitors has already been shown previously (PMID: 32039016).

Response:

We thank the reviewer for this observation. To avoid confusion, we have modified the title to better reflect the core methodology of our work: " Single-Nuclei UPR profiling by flow cytometry reveals bortezomib resistance mechanisms in Multiple Myeloma"

We also acknowledge the relevance of PMID: 32039016. In the revised manuscript, we now clarify that different IRE1α readouts can diverge (bulk sXBP1 mRNA vs downstream transcriptional programs vs nuclear protein localization). In our patient cohort re-analyses, poor outcome under BTZ monotherapy is associated with increased expression of XBP1 transcriptional targets and with reduced expression of IRE1-RIDD targets, consistent with higher inferred IRE1α activity.

We incorporated this clarification in the revised manuscript in the Discussion (paragraph beginning ‘A previous study reported…’) and in the Results: ‘UPR Gene Signatures and Their Clinical Relevance in Multiple Myeloma’ (Figure 8A and Figure 8E, and Fig EV4)

Comment 2:

Highlights at the beginning of the article read like overstatements.

Response:

We appreciate the reviewer’s concern. To address this, we have modified the title for greater accuracy and clarity (see response to previous comment) and removed the “Highlights” section from the beginning of the manuscript, to avoid any perception of overstatement.

Comment 3:

Fig1B-D - P values are missing

Response:

We thank the reviewer for pointing this out. The statistical analysis and P values for Figures 1B-D were already described in the figure legend, but we acknowledge that this was not sufficiently visible. We have now revised the figure legends to show the statistically significant the P values are highlighted with *, **, or *** next to each comparison and ensured that the statistical methods are clearly described in the Methods section.

Comment 4:

Why did the authors choose these two cohorts specifically?

Response:

We selected these two cohorts as they capture the contrast between hematological and solid tumors, exhibit considerable heterogeneity in clinical outcomes, and have both been reported to activate UPR signaling. We assessed the expression of XBP1, ATF6, and ATF4 target genes in breast cancer and acute myeloid leukemia cohorts because these transcription factors represent the three main branches of the unfolded protein response (UPR), which is a critical adaptive mechanism in cancer. Our primary objective was to determine whether the gene signatures of the three UPR branches are always concomitantly activated, or if selective activation of only one branch can occur. This approach is supported by prior evidence showing differential, context-dependent roles of the UPR branches in tumorigenesis.

We have now clarified this rationale in the Results section with the following sentence:

"To gain insights into whether all or specific UPR branches are associated with prognosis in human cancer, we analyzed acute myeloid leukemia (AML) and breast cancer cohorts, representing a hematological and a solid tumor, respectively, which have distinct microenvironment-associated stresses, but display substantial variability in clinical outcomes and have been previously reported to engage UPR signaling[15,16]."

Comment 5:

Fig2B - Tg is used for the analysis of the ATF6 and PERK branches, why is Tm used for the IRE1 branch (it is also activated upon Tg treatment)?

Response:

We thank the reviewer for the question. SNUPR upon tunicamycin (Tm) and thapsigargin (Tg) was performed for all the cell lines. The results for THP-1 cells upon Tg are similar to those for Tm and are shown in Figure 3A. For illustrative purposes, we selected the Tm example for the IRE1 branch to highlight the capacity of SNUPR to detect XBP1s activation (from now Fig. EV1). The comparable Tg results are provided in Figure 3A for reference.

Comment 6:

How do the authors explain the import of puro-peptides into the nucleus?

Response:

The presence of puromycinylated peptides in the nucleus has been observed in multiple studies. While nuclear translation remains a controversial topic, several interpretations exist. Puromycin incorporation can induce release of peptides from ribosomes; many of these peptides are small (<30 kDa) and can freely diffuse through nuclear pores. In our staining protocol, the nucleus represents a substantial portion of the cellular volume and remains permeable after fixation, which may allow retention of these peptides.

In accordance to this, we observed (data not shown) that puromycinylated peptides in the nucleus are of lower molecular weight than those in the cytoplasmic fraction. Independently of the mechanism by which these peptides are present in the nucleus, this phenomenon is consistent with prior work demonstrating nuclear detection of puromycin-labeled nascent chains after short pulses (e.g., **David et al., J Cell Biol 2012**, already cited in the manuscript.

We also explicitly support the validity of nuclear puromycin as a translation proxy by showing the **strong correlation between whole-cell and nuclear puromycin signal** in the revised manuscript (**Appendix Fig. S3E**; Results section ‘Profiling of UPR signalling branches via SNUPR’ and associated text).

Comment 7:

Did the authors verify the activation of XBP1 when ATF6 and PERK are inhibited, and the same for ATF6f and translation when the other two inhibitors are inhibited?

Response:

ATF6 induction was moderate in most of the cell lines studied, and thus we focused primarily on the PERK and IRE1 pathways. In Figure 4, we tested the effects on XBP1 and ATF6 when bypassing PERK-induced translation inhibition using ISRIB, an approach that targets downstream effects on eIF2α.

Comment 8:

Have the authors checked whether the concentration of puromycin used induces ER stress?

Response:

We thank the reviewer for raising this point. In our experiments, we used short 15-minute pulses of puromycin, during which we consistently observed maximal translation measurements. During ER-stress induction kinetics (4-6 hours), puromycin was only added for the final 15 minutes.
Previous publications from our group involving multiple cell types and cell lines have shown that under these

acute and very short incubation before harvesting the cells, puromycin does not induce eIF2α phosphorylation, or XBP1 splicing strongly suggesting no significant ER stress induction at this time. Complete inhibition of protein synthesis by high doses of puromycin for hours or days is cytotoxic. And of course, we do not discard the possibility that if cells were treated for prolonged periods of time with low concentrations of puromycin, ER-stress can be induced. In our study we treat cells with puromycin only for the last 15 minutes before harvesting the cells.

Comment 9:

Cleaved and uncleaved ATF6 forms should be shown on the same Western blot.

Response:

Our study focuses on detecting nuclear translocation of UPR transcription factors by flow cytometry using native-form ATF6 antibodies. With these antibodies validation by Western blot is not compatible. To address the reviewer's request, we used specific inhibitors of the protease cleaving ATF6.
Also during these revisions, we have performed experiments in LP1 MM cells and tested different siRNAs against XBP-1 and ATF6 in LP1 cells. The knock down siRNA against ATF6 showed very low efficiency for ATF6 (at mRNA) and no effect at the protein level.

Comment 10:

Fig3B - XBP1s is regulated from 2h in Fig. 3A but this does not appear to be the case in Fig3A (in the SNUPR part). Can the authors explain this difference?

Response:

The two figures are derived from the same dataset. In THP-1 cells, induction of XBP1s at 2 hours is clear in both figures. Figure 3A presents the data as log-scale fold induction, while Figure 3B shows the same values as a heatmap with a Z-score color scale. The lower induction in HeLa cells is consistently represented in both figures. We have clarified this in the revised figure legend.

Comment 11:

Furthermore, the Z-score legend is not annotated with numbers.

Response:

We have added numeric boundaries to the Z-score color legend in Figure 3B and clarified that the heatmap represents data from three independent experiments, each performed in duplicate.

Comment 12:

Fig5A - 4u8c was demonstrated to display strong off-target effects...

Response:

We agree that 4µ8C can display off-target effects and that our conclusions should rely on orthogonal approaches. Accordingly, we did not base our interpretation on 4µ8C alone. First, we used two chemically distinct IRE1 inhibitors in parallel (4µ8C and KIRA6) and observed consistent attenuation of the XBP1 signal upon IRE1 pathway inhibition (Figure 6B, and Appendix Fig. S3F for siRNA validation). Because KIRA6 can also inhibit other kinases at higher concentrations, we interpret the KIRA6 effects cautiously and emphasize concordant results obtained with 4µ8C and with genetic perturbation.

Second, to provide genetic validation, we performed transient siRNA-mediated knockdown of XBP1 (rather than CRISPR/Cas9 knockout). Complete knockout of XBP1 or IRE1α leads to rapid loss of viability in secretory / ER-stress–dependent hematologic cancer models (including plasmacytoid leukemia lines in our hands, and multiple myeloma models reported previously), which would preclude downstream measurements under bortezomib-induced stress. In addition, siRNA knockdown better mimics the partial pathway inhibition typically achieved with small-molecule therapeutics.

Using siRNA-mediated knockdown of XBP1 (with siCo and siATF6 controls), we validated specificity of the anti-XBP1 SNUPR readout, we observed a reproducible and statistically significant (albeit partial) reduction in the anti–XBP1 nuclear signal upon bortezomib treatment, whereas no decrease was observed with multiple control siRNAs (non-targeting, irrelevant expressed target, and ATF6 siRNA). These results support the specificity of the antibody-based SNUPR XBP1 readout and strengthen our conclusion that the decrease observed with IRE1 inhibition reflects bona fide modulation of the IRE1–XBP1 axis. The new XBP1 knockdown validation data are provided in Appendix Figure S3F within the "Quality control of nuclear extraction for SNUPR profiling and antibodies" section.

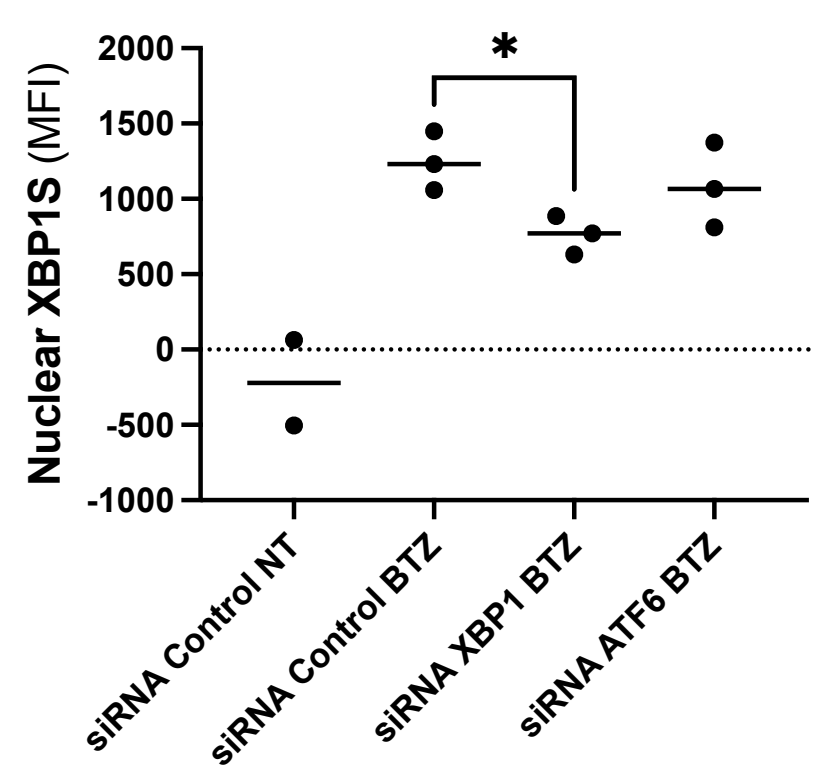

Finally, we note an experimental limitation: combining lipofection with prolonged bortezomib exposure led to substantial cytotoxicity even in siRNA control conditions, reducing the viable fraction and dynamic range for this assay. We therefore cannot extract conclusions from this, but include the full supporting datasets for transparency.

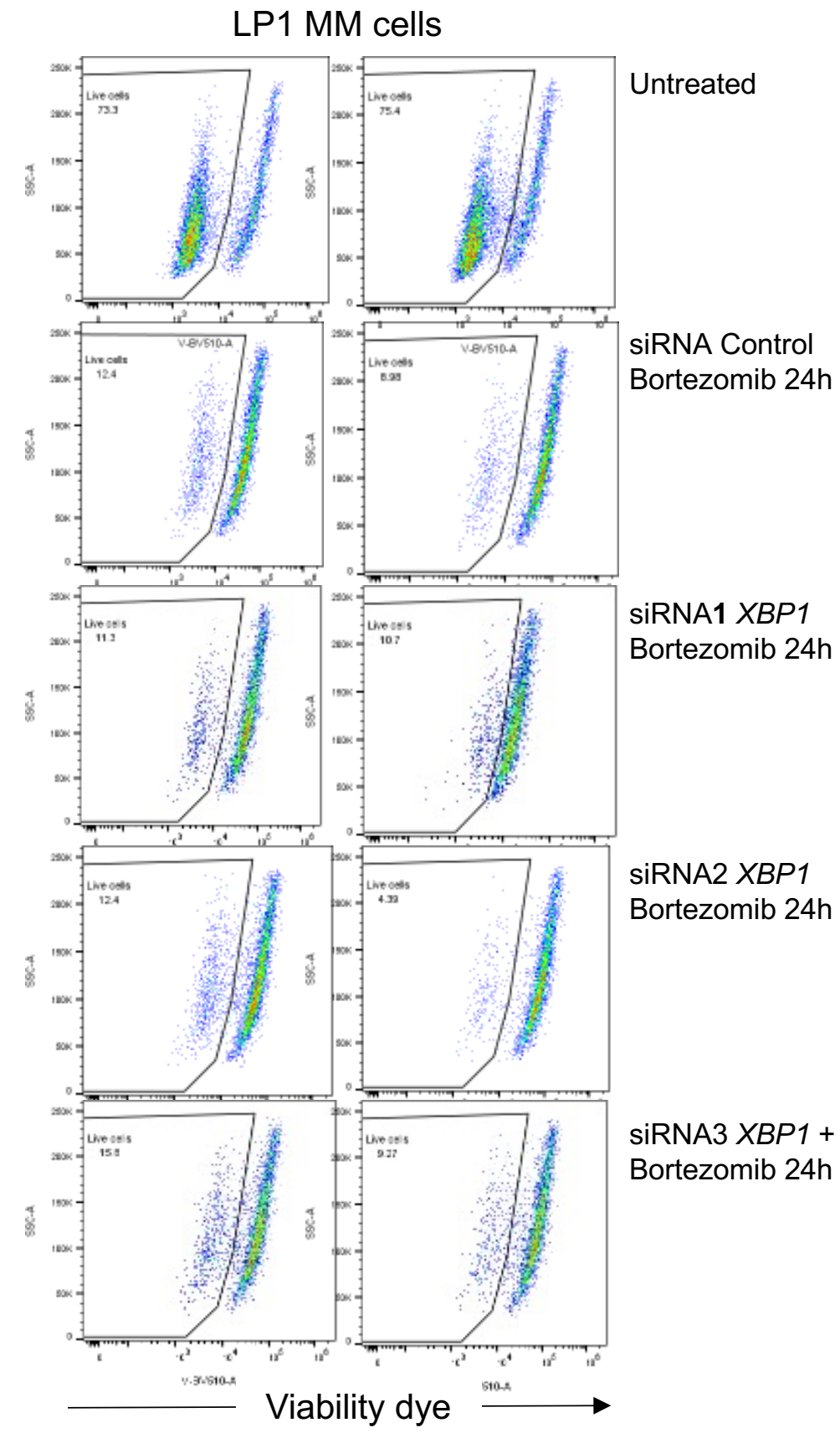

Comment 13:

Since a cross talk between PERK and IRE1 signaling has recently been identified (PMID: 38637497)...

Response:

We acknowledge this important point. While the cited study describes effects observed after prolonged (18 h) stress, our data partially address this through ISRIB (PERK bypass) and IRE1 inhibition experiments (Figures 4 and 5C–D).

Comment 14:

Fig5B (and all cell survival analyses in the manuscript) - the X-axis should start at 0...

Response:

All relevant figures to start the X-axis at 0 and added P values directly to the panels, with detailed statistical information in the legends.

Comment 15:

Fig 5C/D : The authors found with their technique that the increase in ATF6f is not consistent with ATF6 transcriptional activity...

Response:

We agree with the reviewer that ATF6f alone is not a reliable surrogate for ATF6 target gene activation. In Figures 5C/D, we observed a modest ATF6f increase alongside a pronounced XBP1s decrease.
To clarify this, we added in the Discussion: "Our data suggest that simultaneous measurement of XBP1 and ATF6 in the same cells provides a more accurate prediction of transcriptional outcomes than ATF6 measurement alone, due to their overlapping and cooperative target gene regulation."

Comment 16:

Fig6 : Interpretation of Figure 6 does not match the data...

Response:

We corrected the text to state that 8 h of HA15 treatment triggered modest XBP1s translocation and CHOP synthesis/translocation in both cell lines, and revised new Figure 7C text to reflect that translation levels were substantially reduced, not 'slightly reduced'.

Comment 17:

Is there any contribution of RIDD to BTZ and/or HA15 regulation of survival?

Response:

We have now generated an IRE1 RIDD target gene signature and stratified patients by expression of these genes in clinical trial datasets. Our revised analysis indicates that high expression of RIDD targets (reflecting reduced RIDD activity / lower inferred IRE1α output) is associated with improved survival in BTZ monotherapy patients. n contrast to XBP1s target gene expression. This has been added to the Results (new Figure EV4) and discussed accordingly.

This was added to the revised manuscript in the Results: 'UPR Gene Signatures and Their Clinical Relevance in Multiple Myeloma' (Figure 8E and related text), with the target list with statistical significance reported in Appendix Table 1.

Comment 18:

Fig7 : Further explanation on the choice of the genes for XBP1s signature...

Response:

We have expanded the Methods to describe criteria for selecting genes in our XBP1s signature and compared our set with published XBP1 signatures, noting substantial overlap but also inclusion of unique targets relevant to BTZ resistance.

Comment 19:

Discussion points regarding already known crosstalks between UPR branches should be added.

Response:

We have added a new paragraph to the Discussion summarizing recent findings on reciprocal regulation between IRE1 and PERK, and other feedback mechanisms modulating UPR branch activation, citing PMID: 38637497, 36097827, and 30056836.

"Recent advances have revealed that the three canonical branches of the unfolded protein response (UPR), IRE1, PERK, and ATF6, do not function in isolation but instead engage in intricate crosstalk that shapes the overall cellular response to endoplasmic reticulum (ER) stress. IRE1-XBP1s activity has been shown to promote PERK expression during chronic ER stress, establishing a reciprocal regulatory loop between these two branches (PMID: 38637497). Additionally, mutual inhibition and feedback loops involving factors such as p58IPK and GADD34 help fine-tune the sensitivity and timing of each pathway's activation, ensuring an integrated and context-dependent UPR outcome (PMID: 36097827, PMID: 30056836). This dynamic interplay is essential for determining cell fate decisions, as positive feedback between IRE1 and PERK can reinforce the commitment to

apoptosis under unresolved stress, while regulatory crosstalk supports adaptive responses when stress is transient or moderate. Our observations highlighted a hierarchy between the IRE-1/XBP1s and PERK pathways activation…

Point-by-Point Response to Referee #3

Summary Response to Referee #3

We thank Referee #3 for the detailed review of our manuscript and for the constructive feedback provided, which helped us to improve the clarity, depth, and presentation of our study. We appreciate the acknowledgment of the potential of the SNUPR technology to investigate unfolded protein response (UPR) heterogeneity, particularly in complex tissue microenvironments. In the following point-by-point response, we address each comment raised by the reviewer, indicating the changes made to the manuscript and marking in sections that correspond to newly added experiments or analyses.

Comment 1:

The SNUPR technology, when exclusively used on in vitro cultured cell lines, doesn't seem to have advantage over simple Western blotting analysis based on nuclear-cytoplasmic fractionation...

Response:

We thank the reviewer for this comment. We agree that one of the several strengths of SNUPR lies in its application to complex microenvironments and heterogeneous samples. SNUPR has therefore a great advantage over western blots, since it works at single nuclei resolution and gives a clear idea of the homogeneity or not of the UPR intensity and quality in vitro cell culture systems, which are far from being uniform (see also comment 2). We have now performed additional experiments demonstrating the utility of SNUPR ex vivo to analyze mouse brain sections. In this proof of concept experiment, we the capacity of flow cytometry to profile the UPR using SNUPR on distinct cell subsets extracted from the same tissue environment. These results are presented in the revised Figure EV2 and described in the Results section.

Side note: We choose to study brain tissue by SNUPR because of the broad interest of the community of neurodegenerative diseases researchers can use this method.

Comment 2:

Profiling in vitro cultured cell lines with SNUPR revealed a great level of variation and inconsistency...

Response:

While our study uncovered robust trends such as the link between translational arrest and IRE1-XBP1 activity, we acknowledge that further work is needed to fully explain variability between cell lines. Our results also suggest that distinct leukemia and myeloma cell lines require fine-tuned modulation of UPR branches to sustain uncontrolled growth in their respective microenvironments, resulting in an heterogenous pattern uncovered by the SNUPR analysis. We have now extended our analysis focusing on a pathway and gene signatures (RIDD and XBP-1) relevant for predicting multiple myeloma patient outcome upon BTZ treatment. This is described in the expanded Results section and discussed in detail.

Comment 3:

Please be consistent with 'IRE-1/XBP1' or 'IRE-1/XBP1s'...

Response:

We thank the reviewer for this suggestion. For consistency, 'IRE-1/XBP1s' is now used when mentioning both IRE1α and XBP1s, and 'IRE1α' when referring to the sensor alone. All occurrences of 'IRE-1' have been replaced with 'IRE1α' throughout the manuscript.

Comment 4:

'Misfolded proteins': should be 'misfolded and unfolded proteins'.

Response:

The text has been modified accordingly.

Comment 5:

The UPR comprises distinct signaling branches, each activated by the dissociation in the ER lumen of the HSPA5 chaperone (BiP)...

Response:

We expanded the Introduction to note that, in addition to the classical BiP dissociation model, misfolded proteins can directly interact with the luminal domains of ER stress sensors such as IRE1α and PERK, triggering activation independently of BiP release. Now the introduction paragraphs reads:

“The UPR comprises distinct signaling branches, each activated by the dissociation in the ER lumen of the HSPA5 chaperone (BiP) from three ER-resident sensors. In addition to the classical model of UPR activation via BiP dissociation, it is now recognized that misfolded proteins can also directly interact with the luminal domains of ER stress sensors, such as IRE1α and PERK, leading to their oligomerization and activation independently of BiP release (Gardner & Walter, 2011; Karagöz et al, 2017; Wang et al, 2018).”

Comment 6:

'Chemotherapies': proteasome inhibitor should be considered as targeted therapy.

Response:

We have replaced 'chemotherapies' with 'targeted therapies' when referring to proteasome inhibitors.

Comment 7:

For RIDD activity of IRE1, references do not include more recently identified RIDD targets...

Response:

We have generated a large updated RIDD targets gene list to include KLF13, NOTCH1, RICTOR, FAM168b, VPS13C, BIRC6, CUL9, UBR3, CUL5PPP2R1A, RUVBL1, BCAM, TGOLN2, GCLC, and SLC7A11, and reanalyzed survival in multiple myeloma patients stratified MM patients treated with bortezomib or other treatments by expression of these targets. Appendix table 1 show the genes that showed statistically significant

association with patient outcome. As can be seen in Fig 8E and Fig. EV4, RIDD signature and most RIDD targets showed high expression in patients with better prognosis.

Comment 8:

It will be interesting to evaluate the concordance and discordance between XBP1s target gene expression and IRE1 RIDD target gene levels...

Response:

We performed this analysis and found good concordance, suggesting that RIDD and XBP1s-splicing activities can be differentially regulated. This has been added to the Results and discussed in relation to potential implications for UPR modulation in cancer. These results were included in Fig 8E and Fig. EV4, RIDD signature and most RIDD targets showed high expression in patients with better prognosis.

Comment 9:

Fig. S3B: The resolution of the IF images is too low...

Response:

We have improved the resolution and size of the microscopy images in Figure S3B, adding zoomed-in panels of representative cells.

Comment 10:

Fig. S3C: What are the 3 sets of c-n samples...

Response:

We clarified in the legend that the three C/N pairs are independent biological replicates. We also added details of the organelle-specific markers used.

Comment 11:

Antibodies used for flow cytometry... need to be verified with Western blotting.

Response:

Our antibody panel was optimized for flow cytometry, which often requires different clones than those suited for Western blotting. Specificity was confirmed using known pathway modulators and knockdown experiments, as described in Methods.

Comment 12:

Fig. 4: Although the legend mentioned PBMCs, no PBMC data are shown.

Response:

We corrected the legend to reflect that PBMC data are now in Figure 5.

Comment 13:

Fig. S6A: How to explain that ISRIB induced XBP1s mRNA levels...

Response:

We reviewed our raw data and confirmed the time points. The observed pattern may indicate a noncanonical regulatory effect of ISRIB on XBP1s mRNA stability by increasing its translational engagement. This finding is now discussed in the Results section with a note on its potential mechanistic implications.

Comment 14:

GATA3 or BCL6 levels are not enriched in T cells...

Response:

We note that these transcription factors are transiently expressed during activation and differentiation of T cells. The low detection in PBMC nuclei reflects the predominance of resting cells in the samples analyzed.

Comment 15:

A BTZ resistant subpopulation persisted...

Response:

We revised the text for clarity to state that approximately 50% of LP1 cells remained viable at the highest BTZ doses, and that co-treatment with an IRE1α inhibitor eliminated this highly tolerant fraction.

Comment 16:

Add new RIDD targets to Fig. S2.

Response:

We added as described updated RIDD targets Appendix Table 1, Fig EV4; and used them to analyze survival curves in MM patients treated with bortezomib (Fig. 8E, see below).

Figure 8. XBP1 gene signature correlates with unfavorable outcome in Multiple Myeloma patients treated with Bortezomib.

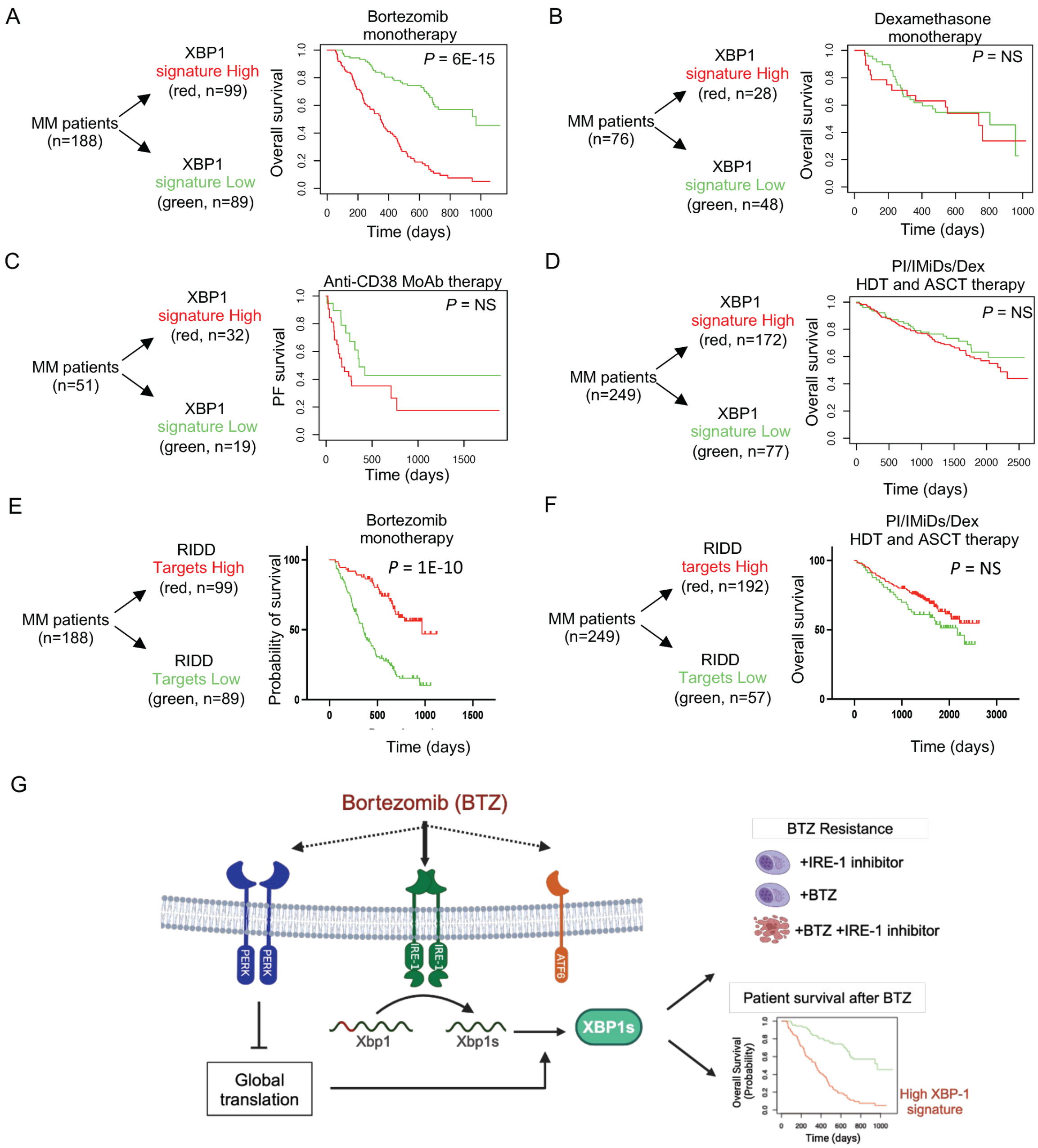

12th May 2026

Dear Dr. Gigan,

Thank you for the submission of your revised manuscript to EMBO Molecular Medicine. We have now received the enclosed report from the two referees who were asked to re-assess the manuscript. As you will see, the referees are now generally supportive, although they have noted a number of -relatively minor- issues. Before we can accept the study for publication, please address the remaining comments raised by the referees.

On a more editorial level:

1. Please remove the track change and submit a clean version.

2. Please add missing callouts for Figure 8G.

3. Remove "Authors' contribution" section from the manuscript file.

4. Please resolve the following author name discrepancy in the submission system (eJP) and manuscript file: Sara Gafoori (manuscript) vs Sara Ghafoori (eJP), Daniela Barros Dos Santos (manuscript) vs Daniela Barros (eJP), Sharon Fischaux (manuscript) vs Sharon Fichaux (eJP), Beatrice Nal (manuscript) vs Béatrice Nal Rogier (eJP) and Jerome Moreaux (manuscript) vs Jérôme Moreaux (eJP).

5. Funding information should be merged with "Acknowledgement". The separate heading "funding" should be removed.

6. The One sentence summary on the title page should be removed.

7. Please provide a completes source data checklist.

8. Appendix:
- Appendix Table 1 is missing.
- Remove the appendix figure legends from the main manuscript text and compile them together with the appendix figures in a separate PDF.
- Label the PDF as "Appendix" and include a table of contents on the first page listing all included items with corresponding page numbers.
- Add the appendix tables to the same file and revise the nomenclature to "Appendix Table S1" and "Appendix Table S2." Please also update all corresponding callouts in the manuscript.
- Appendix Fig. S4: The microscopy images appear pixelated under routine figure analysis. Please provide a higher-resolution version of this figure.

9. Data availability:
- please remove the entire section "resource availability".
-Please use the heading "Data availability" . Since this study does not generate large-scale datasets or new computer code, please only include the following sentence in this section- "This study includes no data deposited in external repositories".

10. "Declaration of interests" should be renamed to "Disclosure and Competing Interests Statement".

11. Please provide a 'Synopsis' to further enhance discoverability. Synopses are displayed on the journal webpage and are freely accessible to all readers. They include a short stand first (maximum of 300 characters, including space) as well as 2-5 one-sentences bullet points that summarizes the paper. Please write the bullet points to summarize the key NEW findings. They should be designed to be complementary to the abstract - i.e. not repeat the same text. We encourage inclusion of key acronyms and quantitative information (maximum of 30 words / bullet point). Please use the passive voice. Please attach these in a separate file or send them by email, we will incorporate them accordingly.

Please provide a visual abstract to illustrate your article as a PNG file 550 px wide x 300 px high.

12. Please provide "The paper explained" : EMBO Molecular Medicine articles are accompanied by a summary of the articles to emphasize the major findings in the paper and their medical implications for the non-specialist reader. Please provide a draft summary of your article highlighting

- the medical issue you are addressing,
- the results obtained and
- their clinical impact.

This may be edited to ensure that readers understand the significance and context of the research. Please refer to any of our published articles for an example.

13. When submitting your revised manuscript, please do not include the Reagents and Tools Table in the manuscript but upload it as a separate file choosing the file type "Reagent Table".

14. The current "Table 1" appears to be missing and may instead correspond to the "Reagents and Tools Table." If so, please remove the "Table 1" callout and refer to it consistently as the "Reagents and Tools Table."

15. Please address the following issues in figure legends:
- Please define the annotated p values ****/***/**/* as well as provide the exact p-values for the same in the legend of figure 7A, C; EV1 Bas appropriate.
-Please note that the exact p values are not provided in the legends of figures 1B, D; 2B-D; 3A, 4A-C; 5D, 6A-D; EV1 A, EV3 A, S1 A-C; S2, S3F, S5.
-Please indicate the statistical test used for data analysis in the legends of figures 7A, C; 8A-F; EV1 B"
-Please note that the box plots need to be defined in terms of minima, maxima, centre, bounds of box and whiskers, and percentile in the legends of figures 2B-D; 3A, 4A, B; 5B, D; 6A, C, D; 7A, C; EV1 A, EV3 A
-Please note that the box plots need to be defined in terms of minima, maxima, bounds of box and whiskers, and percentile in the legends of figures EV2 C
-Please note that information related to n is missing in the legends of figures 2B-D; 4A-C; 5B-D; 6A-D; 7A-D; EV1 A, B; EV3 A-C; S3F
-Please note that the error bars are not defined in the legends of figures 4C, 5C, 6B, 7B, D, EV1 B, EV3 B, C

16. Please correct the order and headings of the sections in the manuscript text file to: Abstract / The Paper Explained /Introduction / Results / Discussion / Methods / Data Availability / Acknowledgements / Disclosure and Competing Interests Statement / References / Figure Legends / Tables / ExpandedView Figure Legends

I look forward to seeing a revised form of your manuscript as soon as possible.

Kind regards,
Jingyi

Jingyi Hou
Senior Editor
EMBO Molecular Medicine

*** Instructions to submit your revised manuscript ***

*** PLEASE NOTE *** As part of the EMBO Publications transparent editorial process initiative (see our Editorial at https://www.embopress.org/doi/pdf/10.1002/emmm.201000094), EMBO Molecular Medicine will publish online a Review Process File to accompany accepted manuscripts.

In the event of acceptance, this file will be published in conjunction with your paper and will include the anonymous referee reports, your point-by-point response and all pertinent correspondence relating to the manuscript. If you do NOT want this file to be published, please inform the editorial office at contact@embomolmed.org.

***** Reviewer's comments *****

Referee #1 (Comments on Novelty/Model System for Author):

n/a

Referee #1 (Remarks for Author):

The authors did a good job at addressing most of the comments raised on the initial version of the manuscript and I appreciated the implementation of a discussion regarding the different UPR branches' cross-talk that has been added.

Considering the interconnection between the different branches of the UPR and the difficulty to untangle the different signaling cascades upon a general activation of endoplasmic reticulum stress I would advise to tone down the following sentence: "SNUPR (Single Nuclei UPR profiling), a flow cytometry-based method that allows to delineate the activation of all three UPR branches, ATF6, IRE-1, and PERK".

Regarding comment 9 (reviewer 1), (i.e., Cleaved and uncleaved ATF6 forms should be shown on the same Western blot), I was unable to see the figure matching the reply to address the reviewer's request (i.e., we used specific inhibitors of the protease cleaving ATF6 -- these proteases are also cleaving other transmembrane transcription factors). Maybe the author forgot to add it.

However, in light of the otherwise very strong evidence, I think the manuscript is, as a whole, very convincing.

Referee #3 (Comments on Novelty/Model System for Author):

The revised manuscript has improved each aspect therefore their scores are now elevated.

Referee #3 (Remarks for Author):

1. Appendix Table 1 is not provided anywhere in the revised submission.
2. EV4: lacking group labels for green- and red-colored lines.
3. The following comments didn't receive responses:

Page 2:
• Typo: "which could not be have been predicted".

Page 3:
• "Overexpressing" should be "higher expression".

Page 4:
• "Overexpressed" mainly referes to "beyond normal range". Above quantile 3 is still within the expression level distribution and should be described as "high expression".

Page 5:
• As the antibodies used for flow cytometry analysis of the UPR transcription factors were not verified using a second approach (e.g. Western blotting), it is not clear whether the lack of induction during Tg treatment (e.g. ATF6f in U937/Hela/HEK293T/KASUMI and XBP1s in MOLT4 cells) was due to expression levels of ATF6f/XBP1s in these cell lines lower than the sensitivity threshold of the antibodies.

Even if the corresponding places were removed in the revised manuscript, those comments still need some type of response to state the reason why they're not relevant anymore.

All editorial requests have been addressed by the authors.

1st Jun 2026

Dear Dr. Argüello,

We are pleased to inform you that your manuscript is accepted for publication and is now being sent to our publisher to be included in the next available issue of EMBO Molecular Medicine.

Your manuscript will be processed for publication by EMBO Press. It will be copy edited and you will receive page proofs prior to publication. Please note that you will be contacted by Springer Nature Author Services to complete licensing and payment information.

You may qualify for financial assistance for your publication charges - either via a Springer Nature fully open access agreement or an EMBO initiative. Check your eligibility: https://link.springer.com/journal/44321/how-to-publish-with-us

Should you be planning a Press Release on your article, please notify the production team via this form in order to coordinate publication and release dates: https://forms.cloud.microsoft/e/eH4A8JVP6g

If you have any questions, please do not hesitate to contact the Editorial Office. Thank you for your contribution to EMBO Molecular Medicine.

Kind regards,
Jingyi

Jingyi Hou
Senior Editor
EMBO Molecular Medicine

>>> Please note that it is EMBO Molecular Medicine policy for the transcript of the editorial process (containing referee reports and your response letter) to be published as an online supplement to each paper. If you do NOT want this, you will need to inform the Editorial Office via email immediately. More information is available here: https://link.springer.com/partners/embo-press/editorial-policies#Peer%20review

EMBO Press Author Checklist

Corresponding Author Name: Rafael J. ARGÜELLO	
Journal Submitted to: EMBO Molecular Medicine	
Manuscript Number: EMM-2025-21836-V2	

USEFUL LINKS FOR COMPLETING THIS FORM
The EMBO Journal - Author Guidelines
EMBO Reports - Author Guidelines
Molecular Systems Biology - Author Guidelines
EMBO Molecular Medicine - Author Guidelines

Reporting Checklist for Life Science Articles (updated January
This checklist is adapted from Materials Design Analysis Reporting (MDAR) Checklist for Authors. MDAR establishes a minimum set of requirements in transparent reporting in the life sciences (see Statement of Task: 10.31222/osf.io/9sm4x). Please follow the journal's guidelines in preparing your
Please note that a copy of this checklist will be published alongside your article.

Abridged guidelines for figures
1. Data
The data shown in figures should satisfy the following conditions:
- the data were obtained and processed according to the field's best practice and are presented to reflect the results of the experiments in an accurate and unbiased manner.
- ideally, figure panels should include only measurements that are directly comparable to each other and obtained with the same assay.
- plots include clearly labeled error bars for independent experiments and sample sizes. Unless justified, error bars should not be shown for technical
- if n<5, the individual data points from each experiment should be plotted. Any statistical test employed should be justified.
- Source Data should be included to report the data underlying figures according to the guidelines set out in the authorship guidelines on Data

2. Captions
Each figure caption should contain the following information, for each panel where they are relevant:
- a specification of the experimental system investigated (eg cell line, species name).
- the assay(s) and method(s) used to carry out the reported observations and measurements.
- an explicit mention of the biological and chemical entity(ies) that are being measured.
- an explicit mention of the biological and chemical entity(ies) that are altered/varied/perturbed in a controlled manner.
- the exact sample size (n) for each experimental group/condition, given as a number, not a range;
- a description of the sample collection allowing the reader to understand whether the samples represent technical or biological replicates (including how many animals, litters, cultures, etc.).
- a statement of how many times the experiment shown was independently replicated in the laboratory.
- definitions of statistical methods and measures:
  - common tests, such as t-test (please specify whether paired vs. unpaired), simple χ^2 tests, Wilcoxon and Mann-Whitney tests, can be unambiguously identified by name only, but more complex techniques should be described in the methods section;
  - are tests one-sided or two-sided?
  - are there adjustments for multiple comparisons?
  - exact statistical test results, e.g., P values = x but not P values < x;
  - definition of 'center values' as median or average;
  - definition of error bars as s.d. or s.e.m.

Please complete ALL of the questions below.
Select "Not Applicable" only when the requested information is not relevant for your study.

Materials

Newly Created Materials	Information included in the manuscript?	In which section is the information available? (Reagents and Tools Table, Materials and Methods, Figures, Data Availability Section)
New materials and reagents need to be available; do any restrictions apply?	Yes	Reagents and Tools Table and Materials and Methods

Antibodies	Information included in the manuscript?	In which section is the information available? (Reagents and Tools Table, Materials and Methods, Figures, Data Availability Section)
For **antibodies** provide the following information:<br>- Commercial antibodies: RRID (if possible) or supplier name, catalogue number and or/clone number<br>- Non-commercial: RRID or citation	Yes	Reagents and Tools Table and Materials and Methods

DNA and RNA sequences	Information included in the manuscript?	In which section is the information available? (Reagents and Tools Table, Materials and Methods, Figures, Data Availability Section)
**Short novel DNA or RNA including primers, probes**: provide the sequences.	Yes	Appendix Table 1

Cell materials	Information included in the manuscript?	In which section is the information available? (Reagents and Tools Table, Materials and Methods, Figures, Data Availability Section)
**Cell lines:** Provide species information, strain. Provide accession number in repository **OR** supplier name, catalog number, clone number, and/**OR** RRID.	Yes	Reagents and Tools Table and Materials and Methods
**Primary cultures:** Provide species, strain, sex of origin, genetic modification status.	Yes	Reagents and Tools Table and Materials and Methods
Report if the cell lines were recently **authenticated** (e.g., by STR profiling) and tested for mycoplasma contamination.	Yes	Reagents and Tools Table (ATCC) and Materials and Methods

Experimental animals	Information included in the manuscript?	In which section is the information available? (Reagents and Tools Table, Materials and Methods, Figures, Data Availability Section)
**Laboratory animals or Model organisms:** Provide species, strain, sex, age, genetic modification status. Provide accession number in repository **OR** supplier name, catalog number, clone number, **OR** RRID.	Yes	Materials and Methods
**Animal observed in or captured from the field:** Provide species, sex, and age where possible.	Not Applicable	
Please detail **housing and husbandry conditions**.	Yes	Materials and Methods

Plants and microbes	Information included in the manuscript?	In which section is the information available? (Reagents and Tools Table, Materials and Methods, Figures, Data Availability Section)
**Plants:** provide species and strain, ecotype and cultivar where relevant, unique accession number if available, and source (including location for collected wild specimens).	Not Applicable	
**Microbes:** provide species and strain, unique accession number if available, and source.	Not Applicable	

Human research participants	Information included in the manuscript?	In which section is the information available? (Reagents and Tools Table, Materials and Methods, Figures, Data Availability Section)
If collected and within the bounds of privacy constraints report on age, sex and gender or ethnicity for all study participants.	Not Applicable	

Core facilities	Information included in the manuscript?	In which section is the information available? (Reagents and Tools Table, Materials and Methods, Figures, Data Availability Section)
If your work benefited from core facilities, was their service mentioned in the acknowledgments section?	Yes	Acknowledgements

Design

Study protocol	Information included in the manuscript?	In which section is the information available? (Reagents and Tools Table, Materials and Methods, Figures, Data Availability Section)
If study protocol has been **pre-registered, provide DOI in the manuscript**. For clinical trials, provide the trial registration number **OR** cite DOI.	Not Applicable	
Report the **clinical trial registration number** (at ClinicalTrials.gov or equivalent), where applicable.	Not Applicable	

Laboratory protocol	Information included in the manuscript?	In which section is the information available? (Reagents and Tools Table, Materials and Methods, Figures, Data Availability Section)
Provide DOI OR other citation details if **external detailed step-by-step protocols** are available.	Yes	Materials and Methods

Experimental study design and statistics	Information included in the manuscript?	In which section is the information available? (Reagents and Tools Table, Materials and Methods, Figures, Data Availability Section)
Include a statement about **sample size** estimate even if no statistical methods were used.	Not Applicable	
Were any steps taken to minimize the effects of subjective bias when allocating animals/samples to treatment (e.g. **randomization procedure**)? If yes, have they been described?	Not Applicable	
Include a statement about **blinding** even if no blinding was done.	Not Applicable	
Describe **inclusion/exclusion criteria** if samples or animals were excluded from the analysis. Were the criteria pre-established?<br><br>If sample or data points were omitted from analysis, report if this was due to attrition or intentional exclusion and provide justification.	Not Applicable	
For every figure, are **statistical tests** justified as appropriate? Do the data meet the assumptions of the tests (e.g., normal distribution)? Describe any methods used to assess it. Is there an estimate of variation within each group of data? Is the variance similar between the groups that are being statistically compared?	Yes	Materials and Methods

Sample definition and in-laboratory replication	Information included in the manuscript?	In which section is the information available? (Reagents and Tools Table, Materials and Methods, Figures, Data Availability Section)
In the figure legends: state number of times the experiment was **replicated** in laboratory.	Yes	Figure legends
In the figure legends: define whether data describe **technical or biological replicates**.	Yes	Figure legends

Ethics

Ethics	Information included in the manuscript?	In which section is the information available? (Reagents and Tools Table, Materials and Methods, Figures, Data Availability Section)
Studies involving **human participants**: State details of **authority granting ethics approval** (IRB or equivalent committee(s), provide reference number for approval.	Not Applicable	
Studies involving **human participants**: Include a statement confirming that **informed consent** was obtained from all subjects and that the experiments conformed to the principles set out in the WMA Declaration of Helsinki and the Department of Health and Human Services Belmont Report.	Not Applicable	
Studies involving **human participants:** For publication of **patient photos**, include a statement confirming that consent to publish was obtained.	Not Applicable	
Studies involving experimental **animals**: State details of **authority granting ethics approval** (IRB or equivalent committee(s), provide reference number for approval. Include a statement of compliance with ethical regulations.	Not Applicable	
Studies involving **specimen and field samples:** State if relevant **permits** obtained, provide details of authority approving study; if none were required, explain why.	Not Applicable	

Dual Use Research of Concern (DURC)	Information included in the manuscript?	In which section is the information available? (Reagents and Tools Table, Materials and Methods, Figures, Data Availability Section)
Could your study fall under dual use research restrictions? Please check biosecurity documents and list of **select agents and toxins** (CDC): https://www.selectagents.gov/sat/list.htm	Not Applicable	
If you used a select agent, is the security level of the lab appropriate and reported in the manuscript?	Yes	Material and Methods
If a study is subject to dual use research of concern regulations, is the name of the **authority granting approval and reference number** for the regulatory approval provided in the manuscript?	Not Applicable	

Reporting

The MDAR framework recommends adoption of discipline-specific guidelines, established and endorsed through community initiatives. Journals have their own policy about requiring specific guidelines and recommendations to complement MDAR.

Adherence to community standards	Information included in the manuscript?	In which section is the information available? (Reagents and Tools Table, Materials and Methods, Figures, Data Availability Section)
State if relevant guidelines or checklists (e.g., **ICMJE, MIBBI, ARRIVE, PRISMA**) have been followed or provided.	Not Applicable	
For **tumor marker prognostic studies,** we recommend that you follow the **REMARK** reporting guidelines (see link list at top right). See author guidelines, under 'Reporting Guidelines'. Please confirm you have followed these guidelines.	Yes	Materials and Methods
For **phase II and III randomized controlled trials**, please refer to the **CONSORT** flow diagram (see link list at top right) and submit the CONSORT checklist (see link list at top right) with your submission. See author guidelines, under 'Reporting Guidelines'. Please confirm you have submitted this list.	Not Applicable	

Data Availability

Data availability	Information included in the manuscript?	In which section is the information available? (Reagents and Tools Table, Materials and Methods, Figures, Data Availability Section)
Have **primary datasets** been deposited according to the journal's guidelines (see 'Data Deposition' section) and the respective accession numbers provided in the Data Availability Section?	Not Applicable	
Were **human clinical and genomic datasets** deposited in a public access-controlled repository in accordance to ethical obligations to the patients and to the applicable consent agreement?	Not Applicable	
Are **computational models** that are central and integral to a study available without restrictions in a machine-readable form? Were the relevant accession numbers or links provided?	Not Applicable	
If publicly available data were reused, provide the respective **data citations in the reference list**.	Yes	Materials and Methods
