# Supplementary material for: Single-nuclei UPR profiling by flow cytometry reveals bortezomib resistance mechanisms in multiple myeloma
Source: EMBO Mol Med. 2026 Jun 29;18(8):3057–85. doi: 10.1038/s44321-026-00469-7 (PMC13470223; doi:10.1038/s44321-026-00469-7)
Supplement: Supplementary file 5 [file 44321_2026_469_MOESM11_ESM.zip › EV2/EV2A/Brain 5_gating strategy.pdf]

Gating for paper

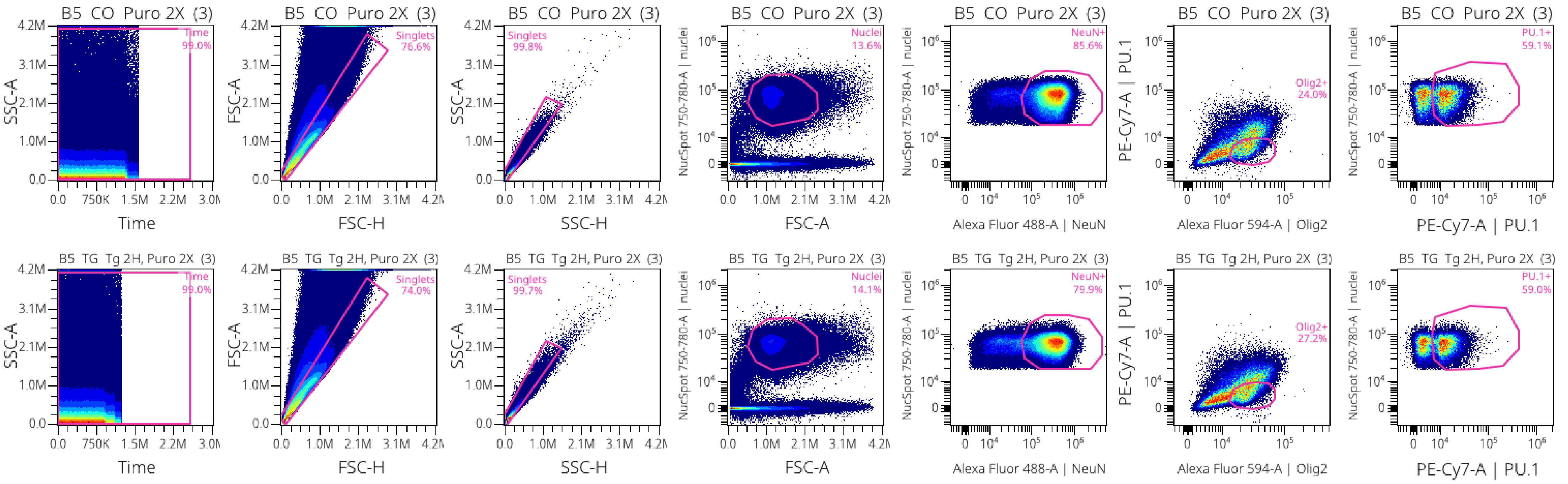

https://app.omiq.ai/workflows/workflow/98011907014907/analysis/figures?taskId=5&parentId=3&taskType=FiguresTask
