# Supplementary material for: Single-nuclei UPR profiling by flow cytometry reveals bortezomib resistance mechanisms in multiple myeloma
Source: EMBO Mol Med. 2026 Jun 29;18(8):3057–85. doi: 10.1038/s44321-026-00469-7 (PMC13470223; doi:10.1038/s44321-026-00469-7)
Supplement: Supplementary file 5 [file 44321_2026_469_MOESM11_ESM.zip › EV2/EV2C/SNUPR prism files/PDF/SNUPR Brain Panel 1 (Figure paper) v2 -per brain-.pdf]

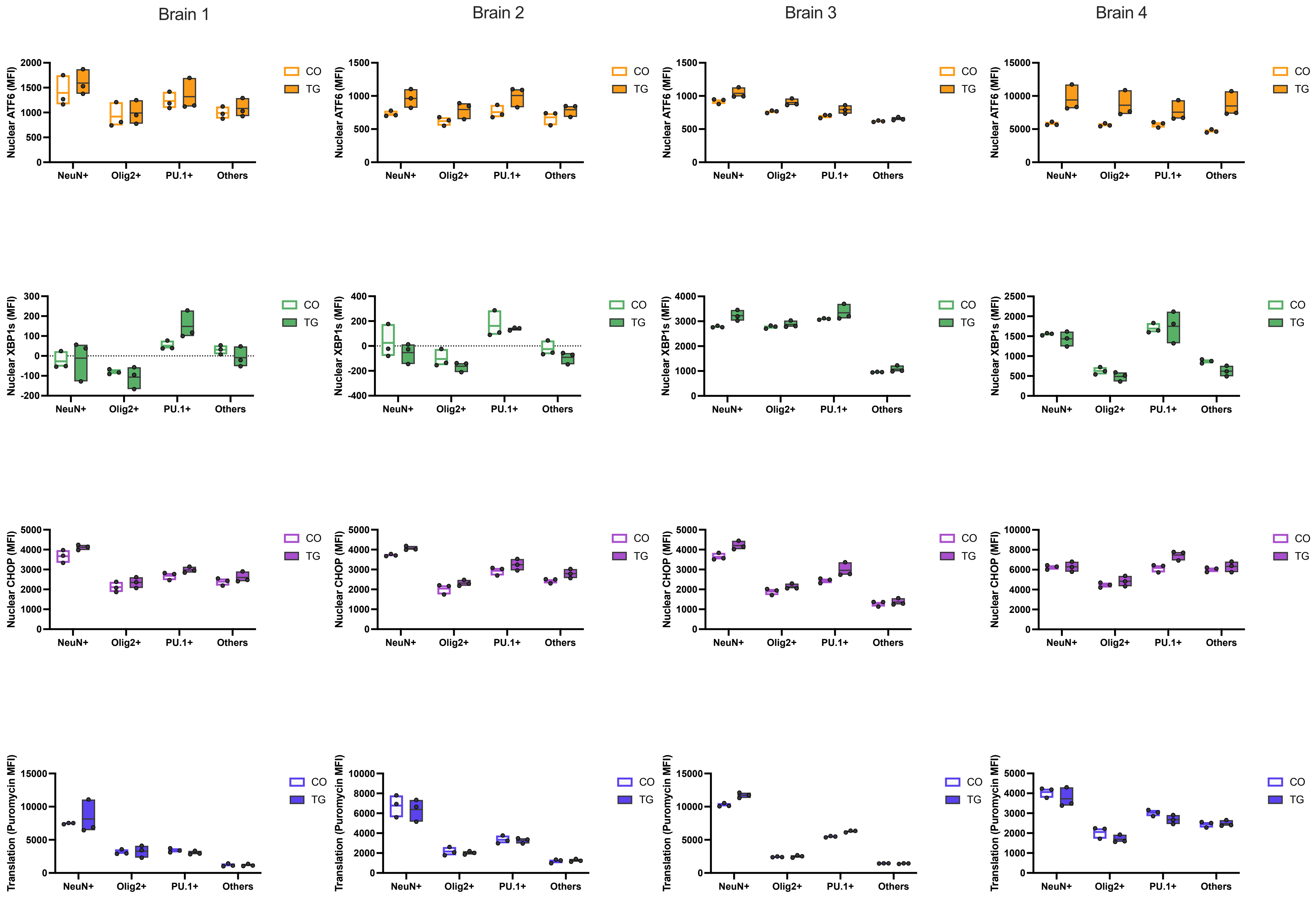

Brain 1
Brain 2
Brain 3
Brain 4
Nuclear ATF6 (MFI)
0
500
1000
1500
2000
NeuN+
Olig2+
PU.1+
Others
CO
TG
Nuclear ATF6 (MFI)
0
500
1000
1500
NeuN+
Olig2+
PU.1+
Others
CO
TG
Nuclear ATF6 (MFI)
0
500
1000
1500
NeuN+
Olig2+
PU.1+
Others
CO
TG
Nuclear ATF6 (MFI)
0
5000
10000
15000
NeuN+
Olig2+
PU.1+
Others
CO
TG
Nuclear XBP1s (MFI)
-200
-100
0
100
200
300
NeuN+
Olig2+
PU.1+
Others
CO
TG
Nuclear XBP1s (MFI)
-400
-200
0
200
400
NeuN+
Olig2+
PU.1+
Others
CO
TG
Nuclear XBP1s (MFI)
0
1000
2000
3000
4000
NeuN+
Olig2+
PU.1+
Others
CO
TG
Nuclear XBP1s (MFI)
0
500
1000
1500
2000
2500
NeuN+
Olig2+
PU.1+
Others
CO
TG
Nuclear CHOP (MFI)
0
1000
2000
3000
4000
5000
NeuN+
Olig2+
PU.1+
Others
CO
TG
Nuclear CHOP (MFI)
0
1000
2000
3000
4000
5000
NeuN+
Olig2+
PU.1+
Others
CO
TG
Nuclear CHOP (MFI)
0
1000
2000
3000
4000
5000
NeuN+
Olig2+
PU.1+
Others
CO
TG
Nuclear CHOP (MFI)
0
2000
4000
6000
8000
10000
NeuN+
Olig2+
PU.1+
Others
CO
TG
Translation (Puromycin MFI)
0
5000
10000
15000
NeuN+
Olig2+
PU.1+
Others
CO
TG
Translation (Puromycin MFI)
0
2000
4000
6000
8000
10000
NeuN+
Olig2+
PU.1+
Others
CO
TG
Translation (Puromycin MFI)
0
5000
10000
15000
NeuN+
Olig2+
PU.1+
Others
CO
TG
Translation (Puromycin MFI)
0
1000
2000
3000
4000
5000
NeuN+
Olig2+
PU.1+
Others
CO
TG
