# Supplementary material for: Single-nuclei UPR profiling by flow cytometry reveals bortezomib resistance mechanisms in multiple myeloma
Source: EMBO Mol Med. 2026 Jun 29;18(8):3057–85. doi: 10.1038/s44321-026-00469-7 (PMC13470223; doi:10.1038/s44321-026-00469-7)
Supplement: Supplementary file 5 [file 44321_2026_469_MOESM11_ESM.zip › EV2/EV2C/SNUPR prism files/PDF/SNUPR Brain panel 1-2 (Figure paper) v3 -normalized data-.pdf]

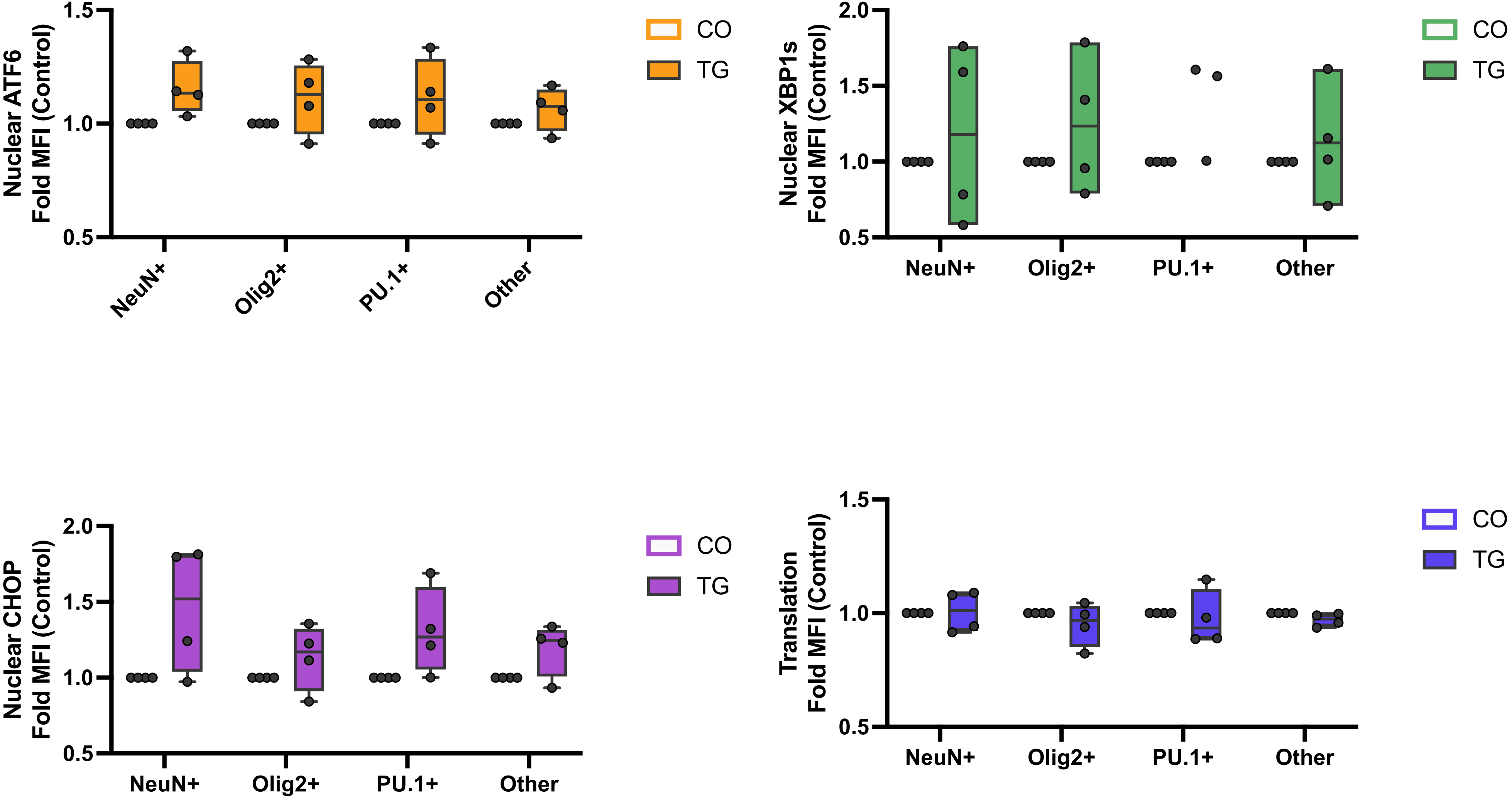
Nuclear ATF6
Fold MFI (Control)
1.5
1.0
0.5
NeuN+
Olig2+
PU.1+
Other
CO
TG
Nuclear XBP1s
Fold MFI (Control)
2.0
1.5
1.0
0.5
NeuN+
Olig2+
PU.1+
Other
CO
TG
Nuclear CHOP
Fold MFI (Control)
2.0
1.5
1.0
0.5
NeuN+
Olig2+
PU.1+
Other
CO
TG
Translation
Fold MFI (Control)
1.5
1.0
0.5
NeuN+
Olig2+
PU.1+
Other
CO
TG
