# Supplementary material for: Single-nuclei UPR profiling by flow cytometry reveals bortezomib resistance mechanisms in multiple myeloma
Source: EMBO Mol Med. 2026 Jun 29;18(8):3057–85. doi: 10.1038/s44321-026-00469-7 (PMC13470223; doi:10.1038/s44321-026-00469-7)
Supplement: Supplementary file 5 [file 44321_2026_469_MOESM11_ESM.zip › EV2/EV2C/SNUPR prism files/PDF/SNUPR Brain Panel 2 (Figure paper) v1 -all brains together-.pdf]

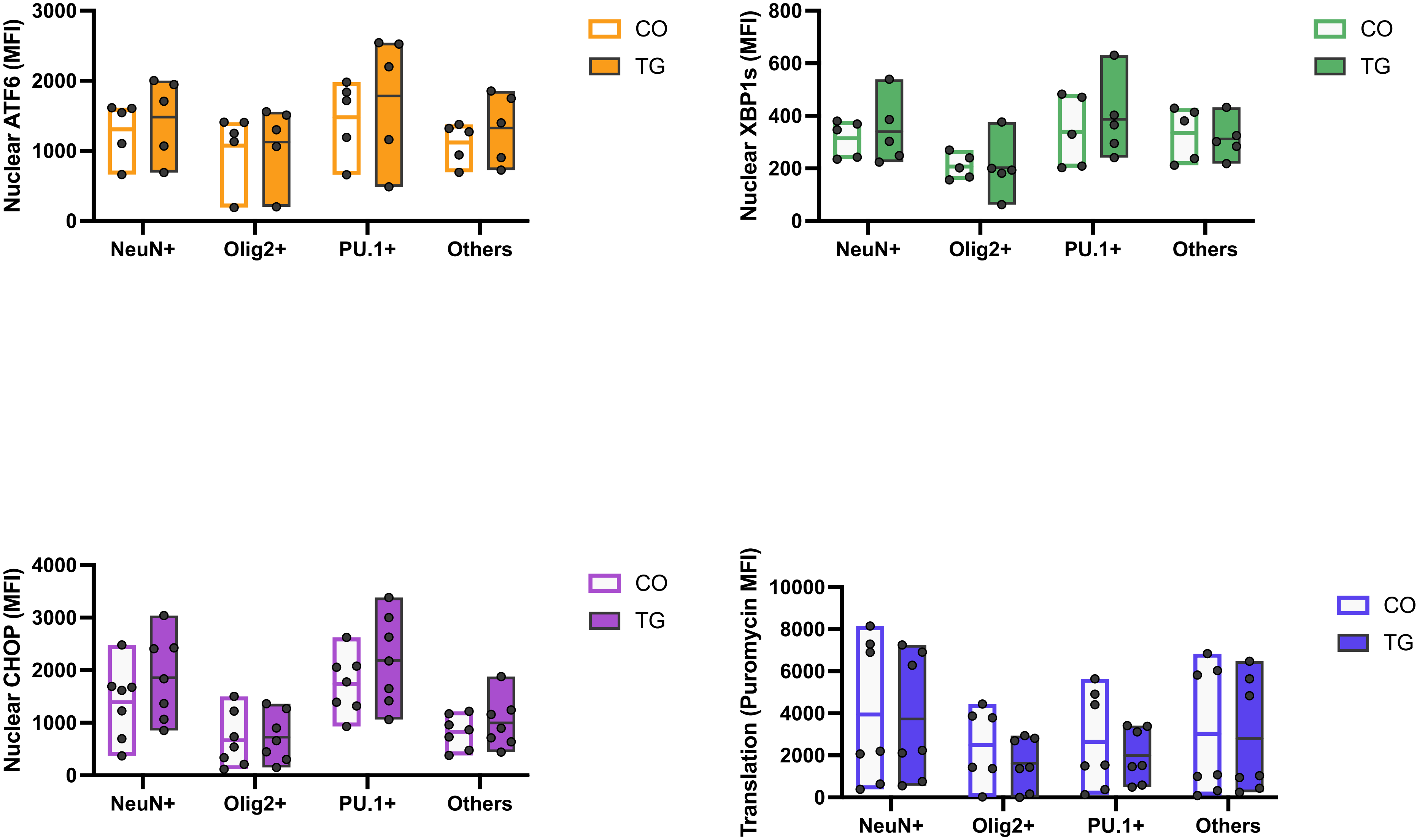

Nuclear ATF6 (MFI)
3000
2000
1000
0
NeuN+
Olig2+
PU.1+
Others
CO
TG
Nuclear XBP1s (MFI)
800
600
400
200
0
NeuN+
Olig2+
PU.1+
Others
CO
TG
Nuclear CHOP (MFI)
4000
3000
2000
1000
0
NeuN+
Olig2+
PU.1+
Others
CO
TG
Translation (Puromycin MFI)
10000
8000
6000
4000
2000
0
NeuN+
Olig2+
PU.1+
Others
CO
TG
