# Supplementary material for: Single-nuclei UPR profiling by flow cytometry reveals bortezomib resistance mechanisms in multiple myeloma
Source: EMBO Mol Med. 2026 Jun 29;18(8):3057–85. doi: 10.1038/s44321-026-00469-7 (PMC13470223; doi:10.1038/s44321-026-00469-7)
Supplement: Supplementary file 6 [file 44321_2026_469_MOESM12_ESM.zip › Extended view EV3/EV3C/08-12-2021-Exp141-toxicityBortezomibCa7/exp141 toxicité bortezomib Cea7.docx]

EXP141 Toxicity of bortezomib avec CeapinA7

# Objectif :

L’objectif est de savoir si la ceapinA7 peu poentialisé l’action du bortezomib via PERK

# Materials

- **Bortezomib (10mM)**
- **GSK 10mM(Cf=100nM)**
- **CeapinA7 10mM**

# Protocol

## Preparation of reagents and solution 20X

Bortezomib : 4µL dans 200µL puis faire dilution en cascade au 1/3

GSK 20X (2µM) : 1µL dans 5mL de PBS

8Tg : 8µL pour 2mL PBS

Contrôle : 8µL DMSO dans 2mL de PBS

## Treatment des cellules

1) Les cellules utilisées sont passées a veille dans du milieu RPMI 10%FCS à 1 million de cellules par mL dans des 96WP (100µL/puit) en présence ou non de 6µM de Ceapin A7
2) Traiter au bortezomib (5µL) en présence ou non de GSK /controle (5µL)
3) Incuber entre 24h à 37°C.
4) Centrifuger les cellules à 300g pendant 5min puit laver une fois avec 200µL de PBS froid.
5) Marquer les cellules à l’aide d’un marqueur live Dead (Zombie Yellow) dilué au 1/400
6) Laver les cellules, puis passer au FACS (LSR561)
