# Supplementary material for: Single-nuclei UPR profiling by flow cytometry reveals bortezomib resistance mechanisms in multiple myeloma
Source: EMBO Mol Med. 2026 Jun 29;18(8):3057–85. doi: 10.1038/s44321-026-00469-7 (PMC13470223; doi:10.1038/s44321-026-00469-7)
Supplement: Supplementary file 11 [file 44321_2026_469_MOESM7_ESM.zip › Figure 5/5A/manual gating strategy.pdf]

Surface gating

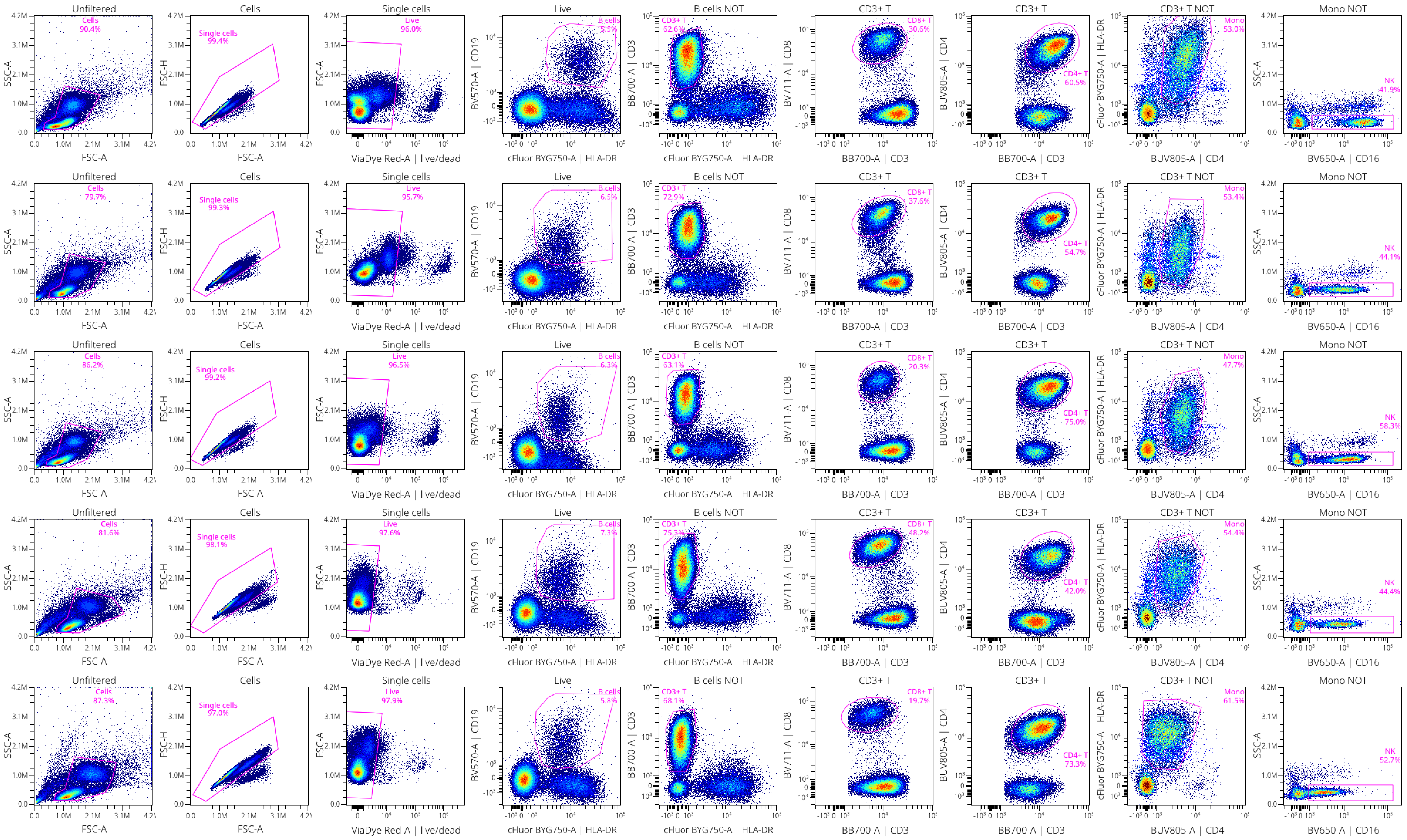

https://app.omiq.ai/workflows/workflow/70913705143034/analysis/figures?taskId=107&parentId=106&taskType=FiguresTask
