# Supplementary material for: Single-nuclei UPR profiling by flow cytometry reveals bortezomib resistance mechanisms in multiple myeloma
Source: EMBO Mol Med. 2026 Jun 29;18(8):3057–85. doi: 10.1038/s44321-026-00469-7 (PMC13470223; doi:10.1038/s44321-026-00469-7)
Supplement: Supplementary file 7 [file 44321_2026_469_MOESM13_ESM.zip › Appendix_Figure_S1.zip!/Appendix Figure S1/Appendix S1B/S1 XBP1s IRE-1 pathway target genes.pdf]

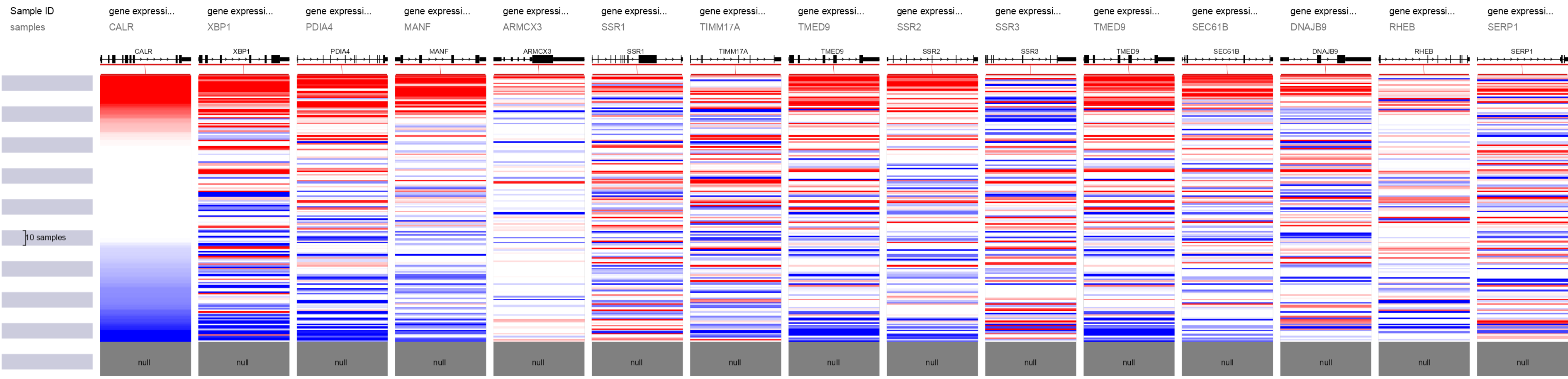

Sample ID
samples
10 samples
gene expressi...
CALR
gene expressi...
XBP1
gene expressi...
PDIA4
gene expressi...
MANF
gene expressi...
ARMCX3
gene expressi...
SSR1
gene expressi...
TIMM17A
gene expressi...
TMED9
gene expressi...
SSR2
gene expressi...
SSR3
gene expressi...
TMED9
gene expressi...
SEC61B
gene expressi...
DNAJB9
gene expressi...
RHEB
gene expressi...
SERP1
null
