# Supplementary material for: Single-nuclei UPR profiling by flow cytometry reveals bortezomib resistance mechanisms in multiple myeloma
Source: EMBO Mol Med. 2026 Jun 29;18(8):3057–85. doi: 10.1038/s44321-026-00469-7 (PMC13470223; doi:10.1038/s44321-026-00469-7)
Supplement: Supplementary file 7 [file 44321_2026_469_MOESM13_ESM.zip › Appendix_Figure_S1.zip!/Appendix Figure S1/Appendix S1C/Appendix S1 AML - ATF4 gen signature Xena.pdf]

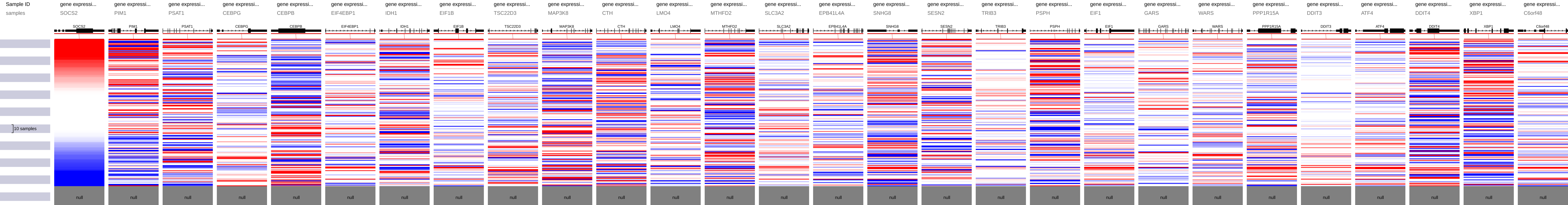
Sample ID
samples
10 samples
gene expressi...
SOCS2
gene expressi...
PIM1
gene expressi...
PSAT1
gene expressi...
CEBPG
gene expressi...
CEBPB
gene expressi...
EIF4EBP1
gene expressi...
IDH1
gene expressi...
EIF1B
gene expressi...
TSC22D3
gene expressi...
MAP3K8
gene expressi...
CTH
gene expressi...
LMO4
gene expressi...
MTHFD2
gene expressi...
SLC3A2
gene expressi...
EPB41L4A
gene expressi...
SNHG8
gene expressi...
SESN2
gene expressi...
TRIB3
gene expressi...
PSPH
gene expressi...
EIF1
gene expressi...
GARS
gene expressi...
WARS
gene expressi...
PPP1R15A
gene expressi...
DDIT3
gene expressi...
ATF4
gene expressi...
DDIT4
gene expressi...
XBP1
gene expressi...
C6orf48
null
