# Supplementary material for: Single-nuclei UPR profiling by flow cytometry reveals bortezomib resistance mechanisms in multiple myeloma
Source: EMBO Mol Med. 2026 Jun 29;18(8):3057–85. doi: 10.1038/s44321-026-00469-7 (PMC13470223; doi:10.1038/s44321-026-00469-7)
Supplement: Supplementary file 7 [file 44321_2026_469_MOESM13_ESM.zip › Appendix_Figure_S3.zip!/Appendix Figure S3/App Fig S3C/App Fig S3C exp113 Nuclear vs Cyto WB/S3C scan B.pdf]

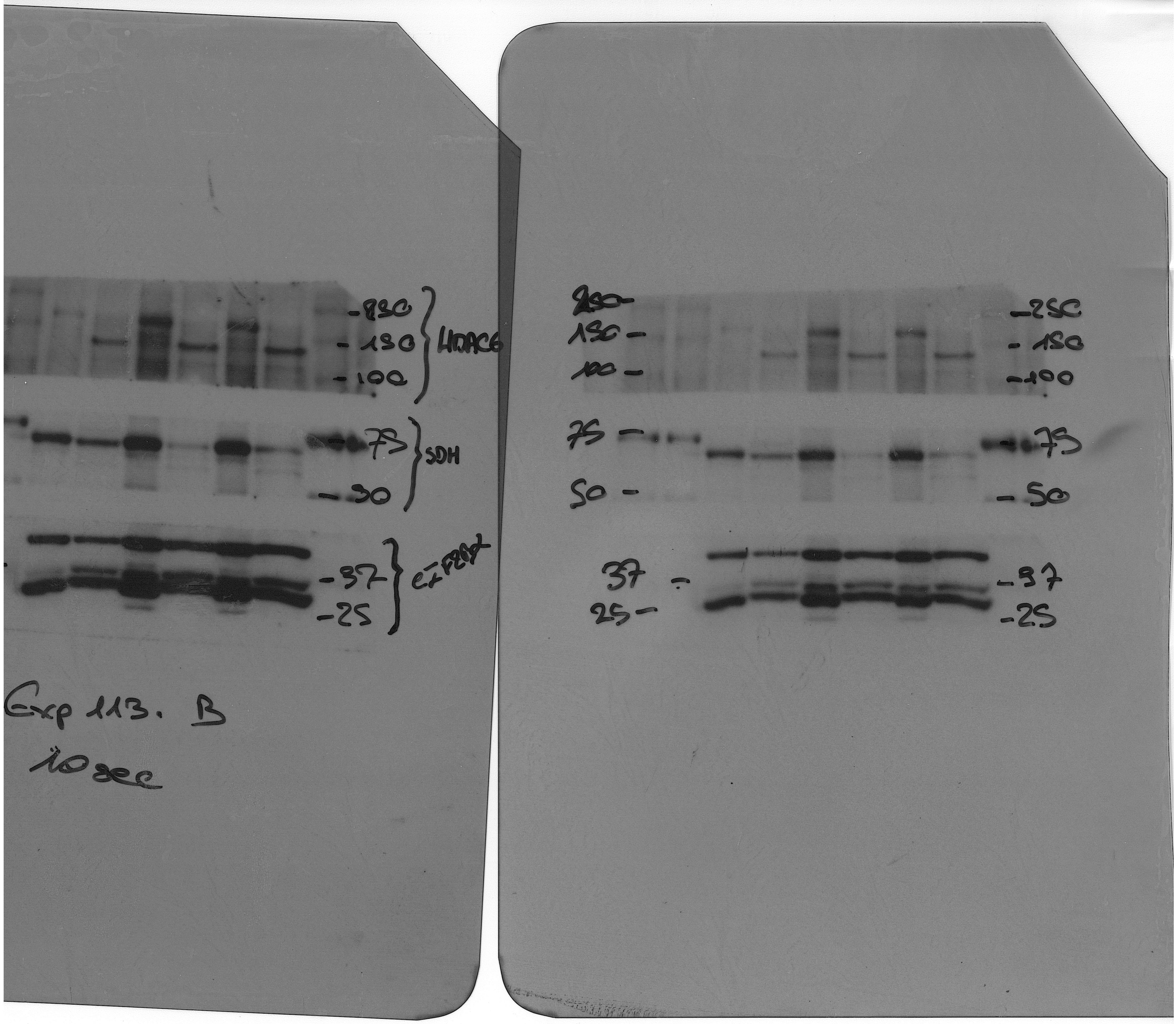

-250
-150
-100
HDAC6
-75
-50
SDH
-37
-25
Exp 113. B
10 sec
250
150
100
75
50
37
25

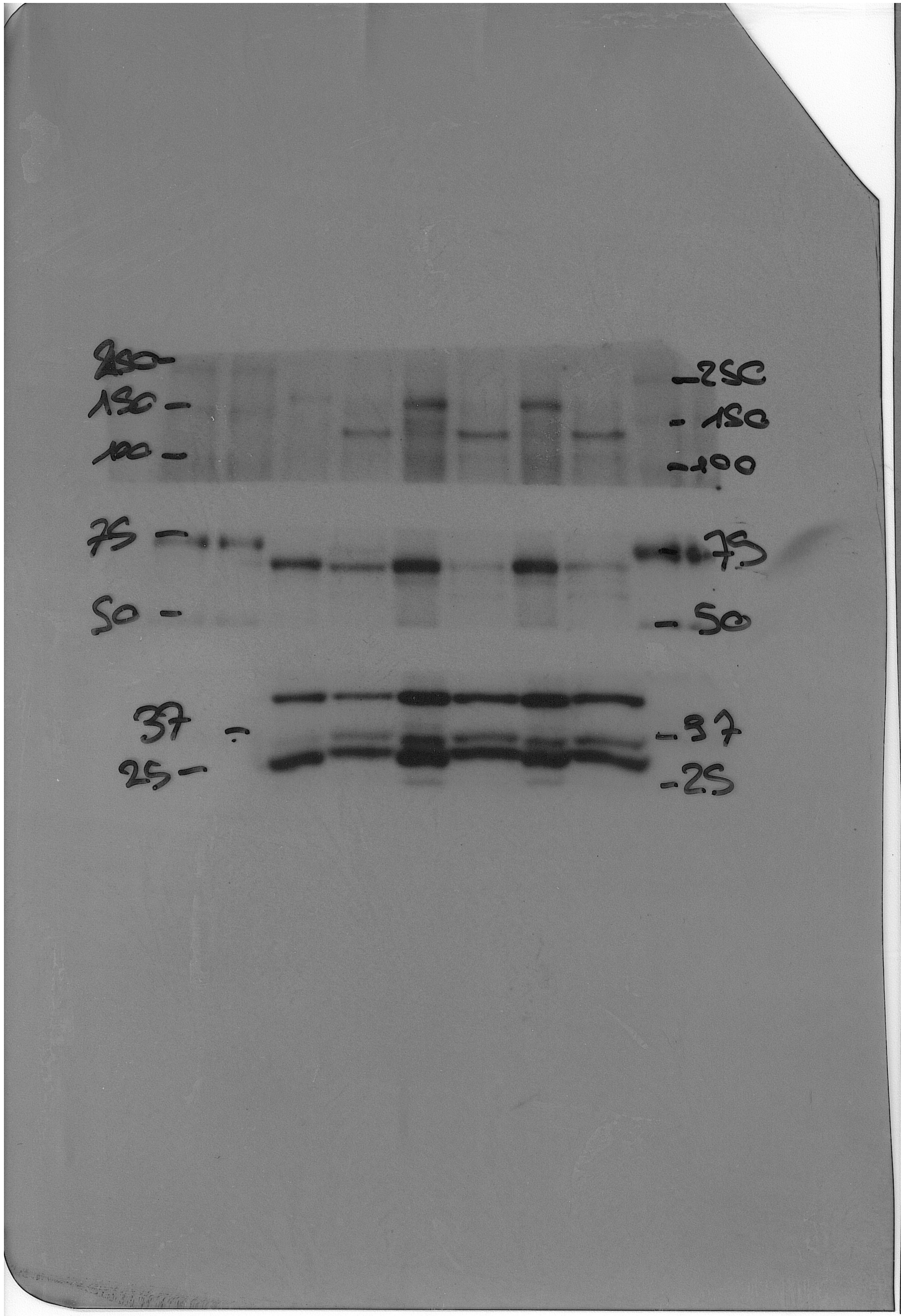
250
150
100
75
50
37
25
250
150
100
75
50
37
25

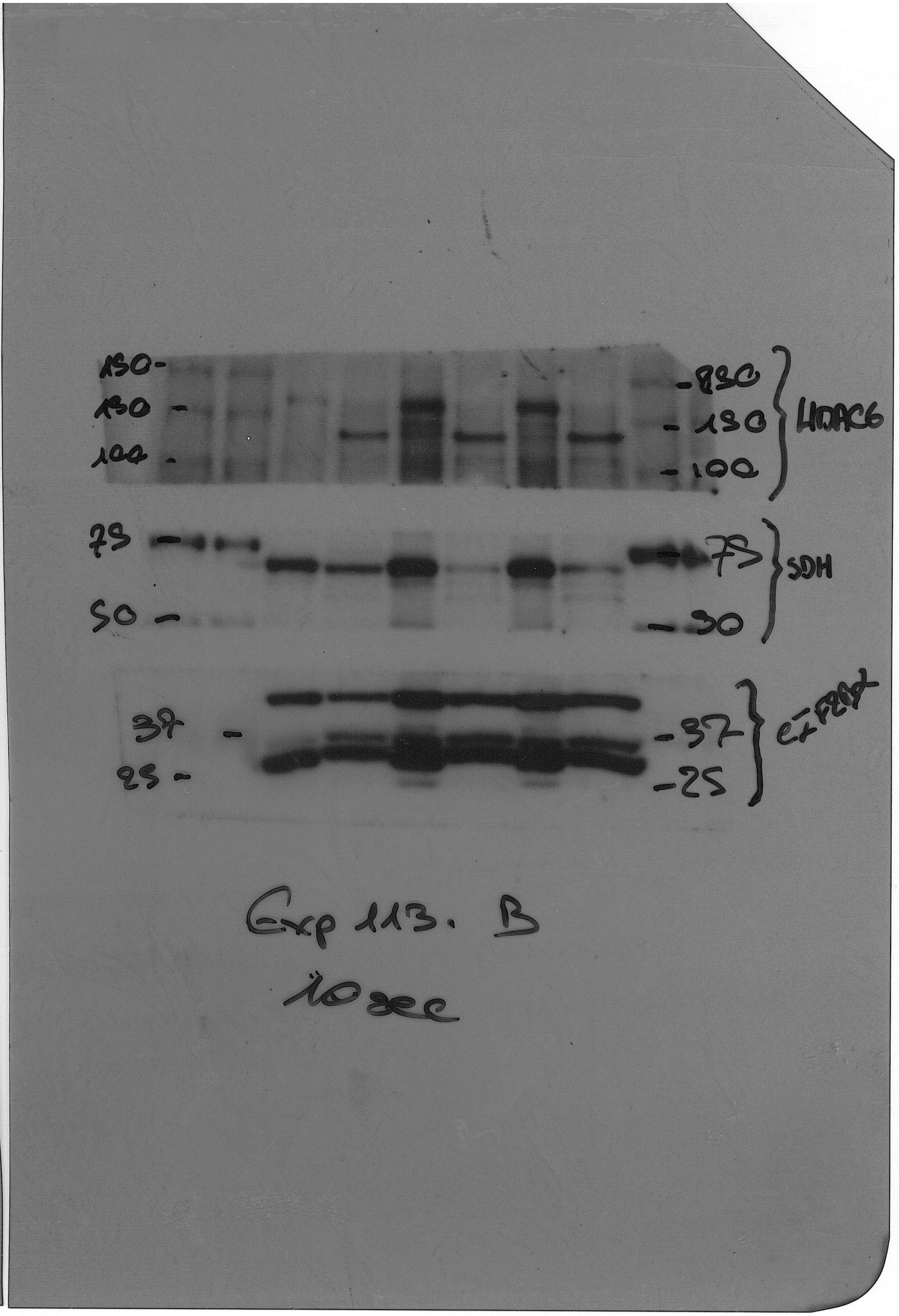
150
100
75
50
37
25
250
150
100
75
30
37
25
HDAC6
SDH
Cl-PARP
Exp 113. B
10 sec
