# Supplementary material for: Single-nuclei UPR profiling by flow cytometry reveals bortezomib resistance mechanisms in multiple myeloma
Source: EMBO Mol Med. 2026 Jun 29;18(8):3057–85. doi: 10.1038/s44321-026-00469-7 (PMC13470223; doi:10.1038/s44321-026-00469-7)
Supplement: Supplementary file 7 [file 44321_2026_469_MOESM13_ESM.zip › Appendix_Figure_S3.zip!/Appendix Figure S3/App Fig S3C/App Fig S3C exp113 Nuclear vs Cyto WB/S3C original scan.pdf]

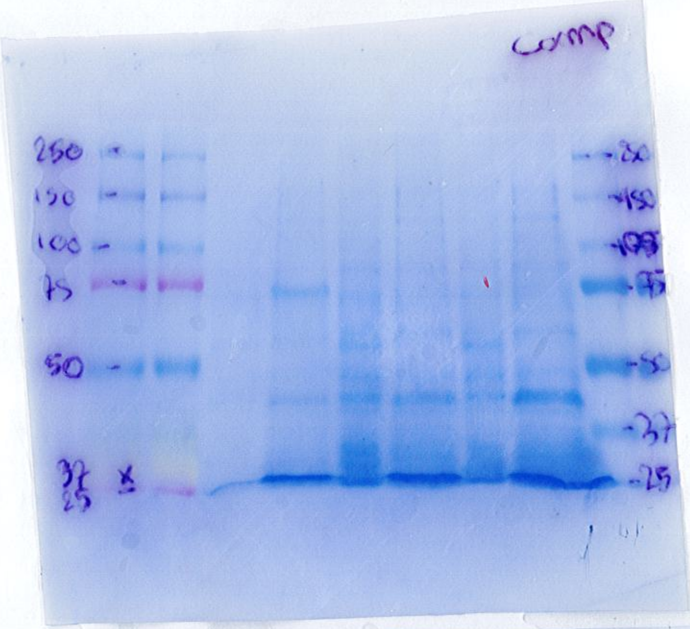
Camp
250
150
100
75
50
37
25

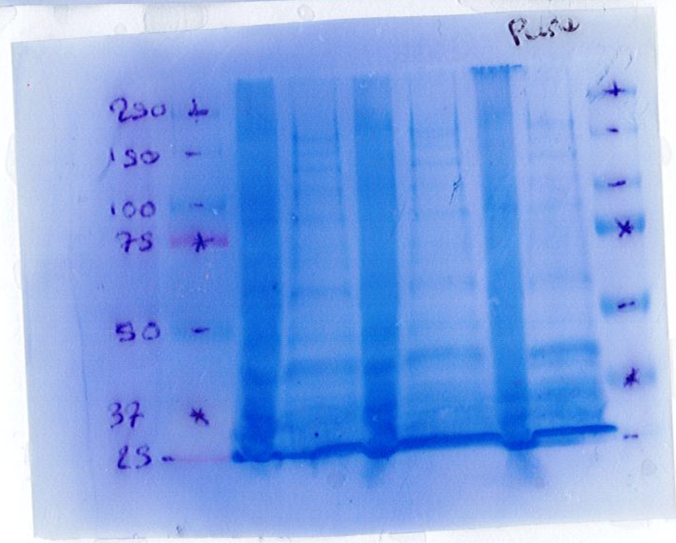
250
150
100
75
50
37
25
